# Alphavirus M1 disrupts super-enhancer-driven oncogenic transcription via non-structural protein NSP2 in osteosarcoma

**DOI:** 10.1038/s41467-026-75013-9

**Published:** 2026-07-01

**Authors:** Jiajun Zhang, Lifeng Yin, Qianqian Han, Yuanyuan Li, Fei Wu, Shanyu Huang, Jiayu Zhang, Yiwei Fu, Guanyu Huang, Yu Xu, Hanxiao Yin, Jiankai Liang, Wenbo Zhu, Yuan Lin, Guangmei Yan, Junqiang Yin, Jingnan Shen, Jing Cai, Weihai Liu

**Affiliations:** 1 Department of Musculoskeletal Oncology, The First Affiliated Hospital of Sun Yat-Sen University, Guangzhou, China; 2 Guangdong Provincial Key Laboratory of Orthopedics and Traumatology, The First Affiliated Hospital of Sun Yat-Sen University, Guangzhou, China; 3 Guangdong Provincial Key Laboratory of Malignant Tumor Epigenetics and Gene Regulation, Sun Yat-Sen Memorial Hospital, Sun Yat-Sen University, Guangzhou, China; 4 Department of Pharmacology, Zhongshan School of Medicine, Sun Yat-Sen University, Guangzhou, China; 5 Biomedical Innovation Center, The Sixth Affiliated Hospital, Sun Yat-Sen University, Guangzhou, China

**Keywords:** Bone cancer, Targeted therapies, Tumour virus infections

## Abstract

Oncolytic virotherapy has shown promise for various cancers, but its application in osteosarcoma (OS) remains underexplored. This study provides evidence that M1, a natural Getah-like alphavirus, exerts oncolytic activity against OS. We demonstrate that OS cells exhibit heightened sensitivity to M1 infection, with its anti-tumor effects not solely dependent on the canonical ER stress-induced apoptosis. Proteomic and ChIP-seq analyses show the DNA-directed RNA polymerase II subunit RPB1 contributes to oncogenic super-enhancer activity and serves as a direct target of M1-mediated regulation. The oncolytic potency correlated positively with the transcriptional dependency on RPB1 within super-enhancer regions. Mechanistically, the viral non-structural protein NSP2 disrupts super-enhancer activity by recruiting the CUL2-RBX1-ELOC complex, which triggers K63-linked ubiquitination and degradation of RPB1. Together, these findings uncover a previously unrecognized mechanism underlying M1 activity in osteosarcoma, support further preclinical evaluation of M1 in this setting, and identify RPB1 as a candidate biomarker for virotherapy response.

## Abstract (web summary)

This study shows that alphavirus M1 can suppress osteosarcoma, a bone cancer, by using viral protein NSP2 to disrupt cancer-driving super-enhancer activity, suggesting a new therapeutic strategy.

## Introduction

Osteosarcoma (OS), the most prevalent primary malignant bone tumor, stands as the second leading cause of cancer-related fatalities among children and adolescents on a global scale^1^. Despite the implementation of combination therapies involving surgical resection and adjuvant/neoadjuvant chemotherapy, 30–50% of OS patients still experience recurrence. The overall 5-year survival rate drops from 60 to 70% to approximately 20% for patients grappling with distal metastasis or local relapse^2,3^. Unfortunately, osteosarcoma exhibits high genetic heterogeneity, including extensive chromosomal rearrangements, amplifications and deletions, which leads to the lack of clear driver genes and effective therapeutic drugs^4^. Additionally, its molecular heterogeneity among different patients and even among different lesions of the same patient severely limits the efficacy of single-target therapies. The progress in improving the outcomes of osteosarcoma patients has remained stagnant over the past three decades, underscoring the urgent demand to explore innovative and effective therapeutic strategies.

In recent years, oncolytic viral therapy has emerged as a promising alternative approach. Oncolytic viruses (OVs) are naturally occurring or genetically engineered viruses designed to selectively infect and destroy tumor cells while sparing healthy cells^5^. These viruses can modulate intracellular processes, including apoptosis, inflammation, angiogenesis, and the cell cycle, ultimately leading to the lysis of cancer cells. Subsequent release of damage-associated molecular patterns (DAMPs) and pathogen-associated molecular patterns (PAMPs) can enhance the host’s anti-tumor immune response^5,6^. There are hundreds of oncolytic viruses derived from more than ten types of viral vectors, including vaccinia virus, coxsackievirus, adenovirus, reovirus, herpes simplex virus, and measles virus, currently in various stages of clinical and preclinical development for cancer therapy^7,8^. The FDA’s approval of Talimogene laherparepvec (IMLYGIC), a genetically engineered herpes simplex virus, for melanoma treatment, has renewed hope for cancer patients^9^. While therapeutic studies using oncolytic viruses to treat various cancers continue to make significant strides, there remains a dearth of research regarding their anti-tumor effects in osteosarcoma.

The M1 virus is a strain of Getah-like alphavirus initially isolated from wild Culex mosquitos in Hainan, China. Its genome comprises an 11.7 kb positive-sense single-stranded RNA, encoding four nonstructural proteins (NSP1-NSP4) and five structural proteins (capsid-E3-E2-6K-E1)^10^. As a novel natural oncolytic virus, alphavirus M1 has demonstrated its capacity to selectively destroy tumor cells through ER stress-mediated apoptosis and autophagy, the release of cytokines, and the activation of natural and acquired immune responses^11^. Furthermore, it exhibits non-pathogenic characteristics in nonhuman primates^12^. One notable advantage of alphavirus M1 is its replication process, which excludes a DNA stage, eliminating the risk of long-term latency or host genomic integration-induced carcinogenic mutations^13^. Numerous ongoing studies have highlighted the clinical translational potential of the M1 virus^14–17^. Recent studies have identified MXRA8 as a key entry receptor directly mediating M1 attachment and infection, providing a mechanistic basis for its tumor selectivity^15^. In addition, LDLR family members have been shown to facilitate the entry of multiple alphaviruses, suggesting that receptor utilization diversity may further contribute to M1’s cell-type–specific infectivity^18^. M1 oncolytic virotherapy is currently in Phase II/III clinical trials in China and Phase I in Japan and has received Orphan Drug Designation from the U.S. Food and Drug Administration for the treatment of liver cancer and malignant glioma.

Here, we report anti-osteosarcoma activity of M1 in cellular models and tumor-suppressive effects in the tested in vivo models, and we further explore the underlying mechanism. When compared to various other-origin tumors, we found that osteosarcoma cells exhibited heightened sensitivity to the M1 virus. Interestingly, we observed that the extent of their ER stress response had a weak correlation with the cytotoxic effects induced by the M1 virus in osteosarcoma. This phenomenon hints that the suppressive effects of M1 observed in osteosarcoma cells may primarily be governed by other as yet undiscovered mechanisms, rather than being predominantly ER stress-based. Through transcriptome sequencing analysis, we discovered that oncogenic transcription in osteosarcoma cells, especially that driven by super-enhancers, was significantly inhibited following M1 virus infection. Super-enhancers are extensive clusters of highly active enhancers on the genome that play a prominent role in promoting the transcription of oncogenes in cancer cells^19,20^. As a result, we postulate that the M1 virus may suppress malignant osteosarcoma cells by targeting the transcriptional promotion driven by oncogenic super-enhancers.

Using ChIP-seq with H3K27ac binding signals, we have verified the selective suppression of oncogenic transcriptional promotion by super-enhancers in M1-infected osteosarcoma cells. In subsequent research, our focus shifted to the enhancer component, the RPB1 protein, which exhibited dramatic changes during the early stages of viral infection. We discovered that RPB1 was abnormally enriched in oncogenic super-enhancer regions and closely correlated with the prognosis of osteosarcoma patients. The M1 virus achieved the inhibition of super-enhancer transcriptional activity by diminishing RPB1’s DNA binding abundance. Moreover, this reduction in RPB1 levels is facilitated by its interaction with the non-structural viral protein NSP2, a product of M1 virus replication and translation within host osteosarcoma cells. This interaction prompts RPB1 degradation via recruiting the CUL2-RBX1-ELOC complex. This study delineates the anti-tumor efficacy of alphavirus M1 in osteosarcoma and uncovers the previously unknown viral oncolytic mechanism that depends on super-enhancers. Furthermore, our results suggest that RPB1 expression may serve as a candidate biomarker to identify subsets of osteosarcoma that are either sensitive or resistant to M1 oncolytic virotherapy.

## Results

### M1 virus infection inhibits osteosarcoma cell malignancies

**Fig. 1:**
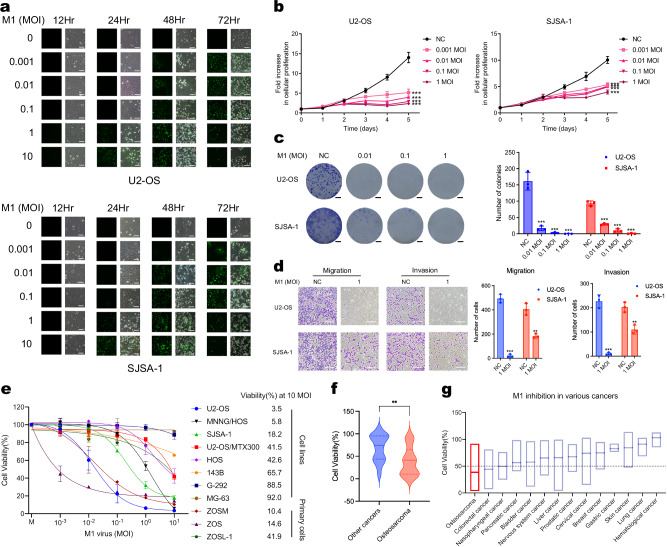
Infection, replication, and anti-tumor effects of M1 virus in osteosarcoma cells. **a** The M1 virus was engineered to express GFP, demonstrating high infection rates and rapid replication in U2-OS and SJSA-1 cells at different time points and viral titers. Scale bars, 100 μm. **b–d** The MTT assay, colony formation assay, and Transwell assay showed significant reductions in cell proliferation (**b**), colony formation (**c**, Scale bars, 5 mm), migration, and invasion (**d**, Scale bars, 150 μm), respectively, in infected osteosarcoma cells. **e** Cell viability was assessed using the MTT assay after 48 h of infection with M1 virus at a range of titers in eight classical osteosarcoma cell lines and three primary tumor cell lines (ZOS, ZOSM, ZOSL-1). *n* = 2 independent experimental batches for each condition, each batch including three biologically independent samples. **f** Comparative analysis of cell viability between other tumor cell lines (*n* = 70) and the aforementioned osteosarcoma cell lines (*n* = 11) after 48 h of treatment with M1 virus at a MOI of 10. **g** Comparative analysis of the inhibitory effect of M1 virus on cell viability across various tumor types. The center line indicates the median, and boxes indicate the minimum-to-maximum range. In (**b**–**d**), data are shown as the mean ± SD, *n* = 3 biologically independent samples. These data were analyzed by two-way ANOVA (**b**), one-way ANOVA (**c**), and two-tailed unpaired Student’s *t* test (**d,f**). **, *P* < 0.01; ***, *P* < 0.001. Source data are provided as a Source Data file.

To evaluate the ability of the M1 virus to infect and replicate in osteosarcoma cells, we utilized a modified M1 virus capable of expressing GFP to treat osteosarcoma cells U2-OS and SJSA-1 at different MOIs and dynamically observed the effect. The results showed that M1 virus could rapidly infect osteosarcoma cells and achieve extensive replication within 24 hours (Fig. 1a and Supplementary Fig. 1). Next, we assessed changes in the malignant characteristics of U2-OS and SJSA-1 cells following M1 virus infection. The results indicated that M1 virus infection significantly inhibited cell proliferation and colony formation of osteosarcoma cells (Fig. 1b, c). The results of transwell assays demonstrated that the migration and invasion abilities of M1-infected osteosarcoma cells were markedly reduced (Fig. 1d). Subsequently, we conducted infection tests on 11 available osteosarcoma cell lines, including three primary osteosarcoma cell lines (ZOS, ZOSM, ZOSL-1). The findings revealed that most osteosarcoma cells exhibited inhibited proliferation following M1 virus infection (Fig. 1e). Interestingly, a comparison with a previously published pan-cancer dataset comprising 70 non-osteosarcoma cancer cell lines showed that osteosarcoma cells were more sensitive to M1 virus-mediated suppression (Fig. 1f and Supplementary Table 1)^12^. Furthermore, the inhibitory effect of M1 virus infection on osteosarcoma cells was more pronounced than in 13 other tumor subtypes (Fig. 1g).

### Virus biodistribution and tumor-suppressive effects in vivo

**Fig. 2:**
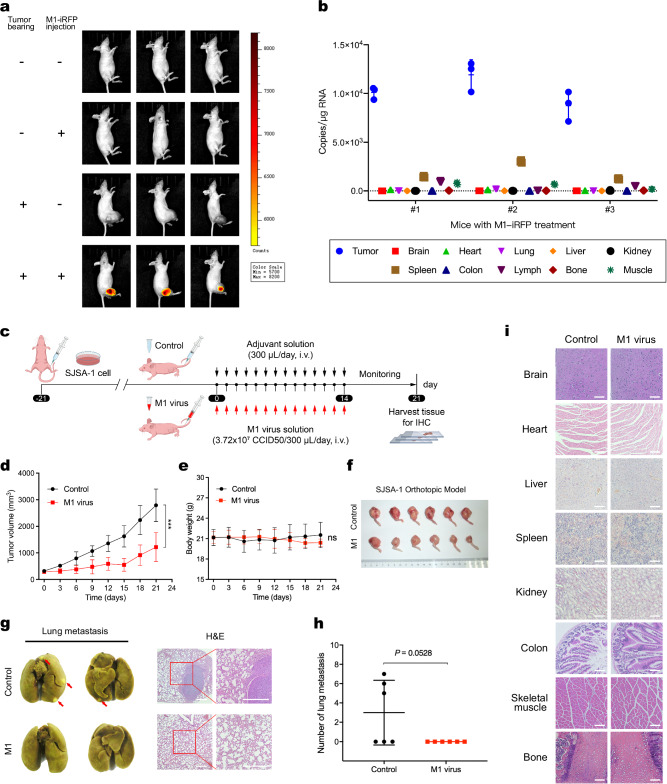
Biodistribution of M1 virus and its therapeutic impact on tumors in vivo. **a** In vivo imaging was used to observe the distribution of M1-iRFP virus in BALB/c nude mice. *n* = 3. **b** RT-qPCR was performed to detect the accumulation of M1 virus in different tissues and organs of BALB/c nude mice. *n* = 3. **c** Schematic diagram of the experimental design for (**d–i)**. Suspended SJSA-1 cells were injected into the tibial bone marrow cavity of BALB/c nude mice to establish an orthotopic transplantation tumor model. After 21 days, the mice were randomly divided into two groups (*n* = 6 per group) to receive either 14 consecutive days of tail vein injections of M1 virus or adjuvant solution, followed by an additional 7 days of observation before euthanasia and sample collection. **d**,**e** Continuous monitoring of tumor growth and body weight curves demonstrated that tail vein injection of M1 virus significantly inhibited tumor volume growth (**d**) without causing noticeable changes in body weight (**e**). **f** At the experimental endpoint, tumors were excised and weighed for comparison between the two groups. **g**,**h** At the experimental endpoint, no visible tumor metastases were observed in the lungs of mice in the M1 virus group, either in gross examination (**g**, left) or histological sections (**g**, right). Scale bars, 500 μm. Quantification of metastatic nodules in histological sections (**h**). **i** Representative H&E images revealed no tissue damage in the brain, heart, liver, spleen, kidneys, colon, skeletal muscle, or bone in M1 treated mice. Representative H&E images are shown from *n* = 6 biologically independent mice per group, with similar results observed across examined mice. Scale bars, 100 μm. In (**d**,**e**,**h**), data are shown as the mean ± SD, *n* = 6 biologically independent mice per group. These data were analyzed by two-way ANOVA (**d**,**e**) and two-tailed unpaired Student’s *t* test (**h**). n.s., not significant; ***, *P* < 0.001. Source data are provided as a Source Data file.

To further investigate the in vivo activity of M1 virus, we firstly intravenously injected M1 virus labeled with the iRFP fluorescent protein into a tibial orthotopic osteosarcoma xenograft model in BALB/c nude mice and monitored its distribution using in vivo imaging technology. The results demonstrated that M1 virus replicated and accumulated extensively within tumor sites, with no significant aggregation observed in normal organ tissues (Fig. 2a). This phenomenon was further validated by viral RNA detection, viral titration, and immunohistochemistry in the organs of BALB/c nude mice (Fig. 2b and Supplementary Fig. 2a, b). Next, we performed serial tail vein injections of the virus in tumor-bearing BALB/c nude mice (Fig. 2c) and found no significant tumor growth during the period of virus administration (Fig. 2d). At the experimental endpoint, tumor growth was significantly inhibited in the M1 virus injection group (Fig. 2f). Lung metastasis of osteosarcoma is a critical factor affecting clinical prognosis^2^. Observations and histological analysis of lung tissues in BALB/c nude mice revealed no tumor metastases in the lungs of the M1 virus injection group, whereas multiple metastatic lesions in the lungs were detected in 3 (50%) of the control group mice (Fig. 2g, h). Additionally, prolonged and continuous virus administration did not cause overt toxic side effects or pathological damage to the examined organs (brain, heart, liver, spleen, lungs, kidneys, colon, skeletal muscle, and bone), as determined by histopathological analysis (Fig. 2e, i). Furthermore, in immunocompetent murine osteosarcoma models, M1 virus exhibited a similar tumor-selective accumulation and distribution pattern (Supplementary Fig. 3a–d). The same virus administration regimen significantly inhibited primary tumor growth and suppressed lung metastasis in immunocompetent mice (Supplementary Fig. 3e–k). Together, these findings support tumor-selective biodistribution and tumor-suppressive activity of M1 in the tested osteosarcoma models, with no overt toxicity detected under the examined conditions.

### ER-stress response is not solely responsible for M1’s effects

**Fig. 3:**
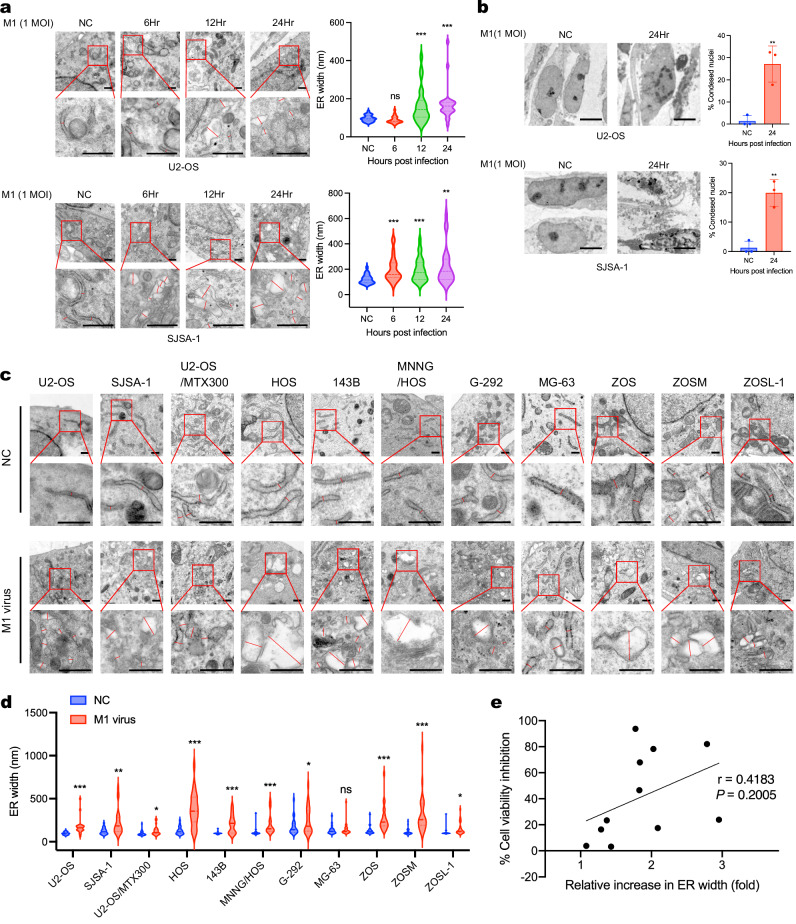
Analysis of M1 virus-induced ER-stress and apoptotic response in osteosarcoma cells. **a** Transmission electron microscopy images showing ER swelling at different time points after M1 virus (1 MOI) infection in U2-OS and SJSA-1 cells (left). Scale bar: 1 μm. Quantification of ER distension is presented (right). **b** Significant signs of cell death, such as nuclear pyknosis, observed under electron microscopy 24 h after M1 virus (1 MOI) infection (left). Scale bar: 5μm. Quantification of condensed nuclei is presented (right). *n* = 3 biologically independent samples. **c**,**d** Observation of ER swelling changes in 11 osteosarcoma cell lines following M1 virus (1 MOI) infection by transmission EM (**c**). Scale bar: 1 μm. Quantification of ER distension is presented (**d**). **e** Correlation analysis between the degree of ER swelling and the cytotoxic effects of the M1 virus. In (**a**,**c**), data are shown as the mean ± SD, *n* = 25 ER width measurements from five fields of view across biologically independent experiments for each condition. These data were analyzed by one-way ANOVA (**a**), two-tailed unpaired Student’s *t* test (**b,d**) and two-tailed Pearson correlation test (**e**). n.s., not significant; *, *P* < 0.05; **, *P* < 0.01; ***, *P* < 0.001. Source data are provided as a Source Data file.

Previous studies have shown that various oncolytic viruses, including M1 virus, trigger intense endoplasmic reticulum (ER) stress upon entering tumor cells, thereby activating the apoptotic process and ultimately leading to host cell lysis^11,16,21–24^. Using electron microscopy, we monitored the intracellular changes in two osteosarcoma cell lines U2-OS and SJSA-1 at different time points following M1 virus infection. The results revealed visible ER swelling and cell death after M1 infection (Fig. 3a, b). Interestingly, we noticed that U2-OS cells, which are more sensitive to M1 virus-induced inhibition compared to SJSA-1 cells, exhibited a delayed and less severe swelling (Fig. 3a). Western blot analysis revealed that the protein levels of ER stress markers BiP and CHOP were not significantly upregulated upon M1 virus infection in U2-OS and SJSA-1 cells (Supplementary Fig. 4a), a pattern consistently observed across all examined osteosarcoma cell lines (Supplementary Fig. 4b). Further electron microscopy analysis of 11 osteosarcoma cell lines post-infection revealed that the degree of ER stress response did not strongly correlate with the extent of M1-induced cell death (Fig. 3c-e). Furthermore, M1 virus exhibited efficient infection and replication capabilities in all examined osteosarcoma cells, yet these parameters did not correlate with the sensitivity of these cells to M1 (Supplementary Fig. 5a–c). Among previously reported host cell receptors that assist alphavirus entry, MXRA8 showed a correlation with the anti-osteosarcoma effect of M1 (Supplementary Fig. 5d). However, although LDLR has been reported to directly bind alphavirus and act as an entry receptor, it was not found to be involved in the oncolytic effect of M1. These findings suggest that the inhibitory effect of M1 virus on osteosarcoma may involve other, as yet unidentified, mechanisms.

### M1 virus disrupts super-enhancer activity in osteosarcoma

**Fig. 4:**
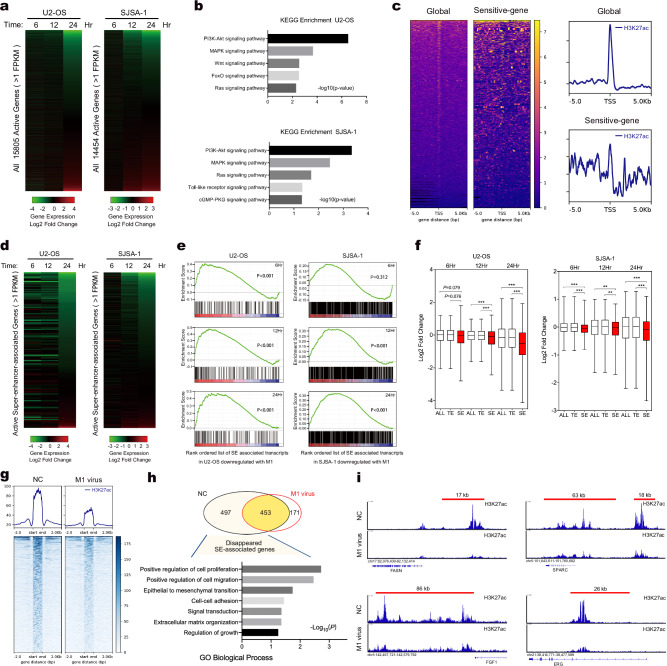
Selective inhibition of tumor-associated super-enhancer activity in osteosarcoma cells. **a** Heatmap showing transcriptomic changes in osteosarcoma cells at 6, 12, and 24 h post-M1 virus (1 MOI) infection. **b** KEGG clustering analysis of significantly downregulated genes in osteosarcoma cells early after virus infection. KEGG pathway enrichment analysis was performed using a one-sided hypergeometric test. **c** ChIP-seq results showing abnormal enrichment of H3K27ac signals in the TSS regions of genes sensitive to M1 virus (1 MOI) infection in SJSA-1 cells. **d** Heatmap showing changes in the expression of super-enhancer-associated genes at different time points after M1 virus infection. **e** GSEA analysis indicating the selective inhibition of osteosarcoma super-enhancer-associated genes by M1 virus infection. GSEA was performed using permutation-based testing. **f** Box plot comparing the expression changes of genes associated with all enhancers (ALL), typical enhancers (TE), and super-enhancers (SE) in U2-OS and SJSA-1 osteosarcoma cells after M1 virus infection. In U2-OS cells, *n* = 15,805 genes for ALL, *n* = 15,672 genes for TE, and *n* = 133 genes for SE. In SJSA-1 cells, *n* = 14,454 genes for ALL, *n* = 13,510 genes for TE, and *n* = 944 genes for SE. The center line indicates the median, boxes indicate the interquartile range, and whiskers indicate the 1st to 99th percentiles. P values were determined by one-way ANOVA. **, *P* < 0.01; ***, *P* < 0.001. **g** Significant changes in the H3K27ac signal at osteosarcoma-specific SE regions of SJSA-1 cells before and after M1 virus infection. **h** After M1 virus infection, a large number of SE elements disappear (upper); functional clustering analysis of genes associated with these disappeared SEs (lower). GO biological process enrichment analysis was performed using a one-sided hypergeometric test. **i** IGV showing H3K27ac signal changes in the genomic regions of cancer gene-related SEs that disappear after M1 virus infection in SJSA-1 cells.

To gain mechanistic insight into M1-induced malignancy suppression in osteosarcoma, we firstly utilized RNA-seq to explore the biological changes in osteosarcoma cell lines U2-OS and SJSA-1 after M1 virus infection. The results showed that the expression levels of numerous genes were significantly downregulated in the early stages of M1 infection (Fig. 4a). Further analysis revealed that, in addition to the unfolded protein response pathway associated with viral infection (Supplementary Fig. 6a), early transcriptional changes were enriched in several previously reported osteosarcoma-oncogenic signaling pathways (Fig. 4b). To clarify the changes in genomic transcriptional activity induced by M1 virus infection, we performed ChIP-seq with the transcriptional activity marker H3K27ac. The results demonstrated that the enhancers of downregulated genes exhibited super-enhancer-like widespread and abnormally high H3K27ac signals prior to infection (Fig. 4c).

Numerous studies, including our previous work, had identified and characterized the super-enhancer landscape in osteosarcoma, underscoring its significant role in osteosarcoma progression^25–28^. Based on these, we attempted an in-depth analysis and found that the expression of driver genes associated with tumor-related super-enhancers was selectively suppressed after M1 virus infection (Fig. 4d–f and Supplementary Fig. 6b). Previous studies have reported that H3K27ac signals represent the transcriptional activity of super-enhancers and serve as a hallmark for their identification^29^. We observed a significant global reduction in H3K27ac signals in the super-enhancer regions of osteosarcoma cells following M1 virus infection (Fig. 4g). Additionally, many super-enhancers, which are associated with tumor signaling pathways and oncogenes, lost their super-enhancer characteristics after infection (Fig. 4h), including those linked to genes such as *FASN*, *SPARC*, *FGF1*, and *ERG* (Fig. 4i). The above findings suggest that M1 virus selectively suppresses the functional activity of tumor-related super-enhancers and the expression of their associated genes in osteosarcoma.

### RPB1 down-regulation linked to M1-induced super-enhancer suppression

**Fig. 5:**
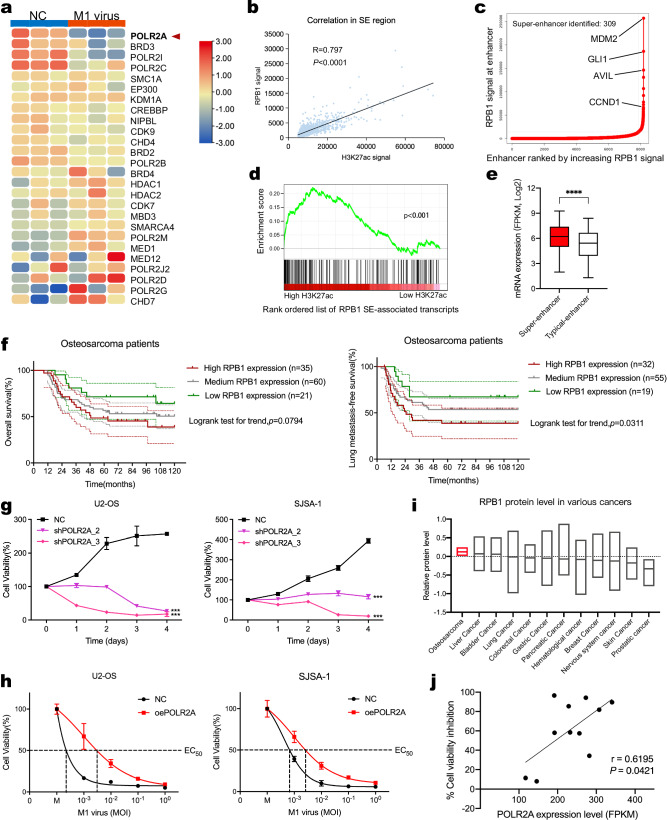
M1 virus infection suppresses the RPB1 protein of the transcription complex, which binds to super-enhancer regions. **a** Changes in transcription complex-related proteins after 12 h of M1 virus infection were analyzed using TMT-based proteomics. **b** The signal abundance of RPB1 positively correlates with H3K27ac in super-enhancer regions. **c** Identification of super-enhancer catalogs using RPB1 signals via the ROSE algorithm. **d** GSEA analysis reveals that RPB1-associated super-enhancer genomic regions exhibit high H3K27ac signal characteristics. P values were calculated by permutation testing. **e** Transcriptional activities of RPB1-associated super-enhancer genes are significantly elevated (*n* = 453 genes for SE, and *n* = 9490 genes for TE.). The center line indicates the median, boxes indicate the interquartile range, and whiskers indicate the 5th to 95th percentiles. **f** Kaplan-Meier (KM) survival analysis of RPB1 protein expression with overall survival (116 cases) (left) and lung metastasis-free survival (106 cases)(right) in tumor specimens from osteosarcoma patients. The data are presented as the means ± 95% CI. **g** Changes in cell viability of U2-OS and SJSA-1 cells after *POLR2A* knockdown as detected by MTT assay. **h** The EC50 shifts of U2-OS and SJSA-1 cells treated with escalating titers of M1 virus with or without *POLR2A* overexpression for 4 days. In (**g**,**h**), data are shown as the means ± SD, *n* = 3 biologically independent samples. **i** Proteomic expression level of RPB1 across various types of human cancer cells. Data were retrieved from CCLE project from Broad Institute. The center line indicates the median, and boxes indicate the minimum-to-maximum range. **j** Correlation analysis between the *POLR2A* expression level and the cytotoxic effects of the M1 virus of 11 osteosarcoma cell lines. These data are were analyzed by two-tailed Pearson correlation test (**b,j**), two-tailed unpaired Student’s t test (**e**), log-rank test (**f**), and two-way ANOVA (**g**). ***, *P* < 0.001; ****, *P* < 0.0001. Source data are provided as a Source Data file.

The potent transcriptional activation activity of super-enhancers relies on the aberrant assembly of RNA Polymerase II (RNAPII), key transcription factors, cofactors, and histone modification marks^30,31^. To investigate the reasons for the decline in super-enhancer activity, TMT-based proteomics was used to detect protein-level changes in these super-enhancer complex components in osteosarcoma cells after M1 infection. The results showed that RPB1 (*POLR2A*), a functional subunit of RNAPII that is involved in the transcriptional activation of super-enhancers, was significantly downregulated (Fig. 5a). RNAPII, the core component of the super-enhancer-mediated transcriptional activating system, is a megacomplex consisting of 12 subunits, with RPB1 being the largest and catalytic subunit^32^. To elucidate the relationship between RPB1 abundance and the transcriptional activity of super-enhancers, ChIP-seq was used to map the genomic distribution of RPB1 in osteosarcoma cells. The results revealed a positive correlation between RPB1 signal density and H3K27ac signal density at super-enhancer regions (Fig. 5b). Using RPB1 as a marker, the ROSE algorithm was employed to identify RPB1-associated super-enhancers (RPB1-SEs) in osteosarcoma (Fig. 5c). Clustering analysis indicated that these RPB1-enriched SEs are closely associated with tumor-related biological processes and signaling pathways (Supplementary Fig. 7a). RPB1-SE regions exhibited significantly higher H3K27ac signals than typical enhancers, and their associated genes were highly expressed (Fig. 5d, e). In addition, a comparison of ChIP-seq data from M1-infected and non-infected osteosarcoma cells revealed that M1 virus infection caused a reduction in RPB1 signals at SEs (identified by H3K27ac) (Supplementary Fig. 7b).

As a functional subunit of the RNAPII complex, the relationship between RPB1 protein and tumor malignancy or patient prognosis has rarely been reported. Analysis of osteosarcoma specimens from 116 patients revealed a negative correlation between RPB1 protein level and both overall survival (OS) and lung metastasis-free survival (LMFS) (Fig. 5f). Notably, similar correlations were observed in other cancers, including sarcoma, skin cutaneous melanoma, bladder cancer, hepatocellular carcinoma, colorectal cancer, as well as head and neck squamous cell carcinoma (Supplementary Fig. 8a, b). Further, knockdown of the *POLR2A* gene (encoding RPB1 protein) significantly inhibited the proliferation and colony formation ability of osteosarcoma cells (Fig. 5g and Supplementary Fig. 9a, b), whereas overexpression of *POLR2A* antagonized the inhibitory effects of M1 virus on osteosarcoma (Fig. 5h and Supplementary Fig. 10a–c). Given that RPB1 is a critical inhibitory target of the M1 virus, we analyzed the relationship between RPB1 expression and the pronounced oncolytic effect of M1 in osteosarcoma. Our results revealed that, compared to other tumor types, osteosarcoma cells exhibit the highest levels of RPB1 protein expression (Fig. 5i). To further validate this expression pattern, we examined the correlation between the oncolytic effect and gene expression. Consistently, we found that osteosarcoma cell lines with high *POLR2A* expression were more sensitive to M1 virus infection (Fig. 5j). This mechanism was further corroborated in vivo, where IHC staining of tumor sections demonstrated that areas positive for M1 virus infection displayed concurrent downregulation of RPB1 and Ki-67 (Supplementary Fig. 11).

These findings suggest that the M1 oncolytic virus downregulates the RPB1 protein, a key factor for super-enhancer activity, and that RPB1 expression is associated with osteosarcoma malignancy and might be a potential biomarker indicating the sensitivity to M1 virus.

### M1 virus triggers RPB1 protein degradation via ubiquitination

**Fig. 6:**
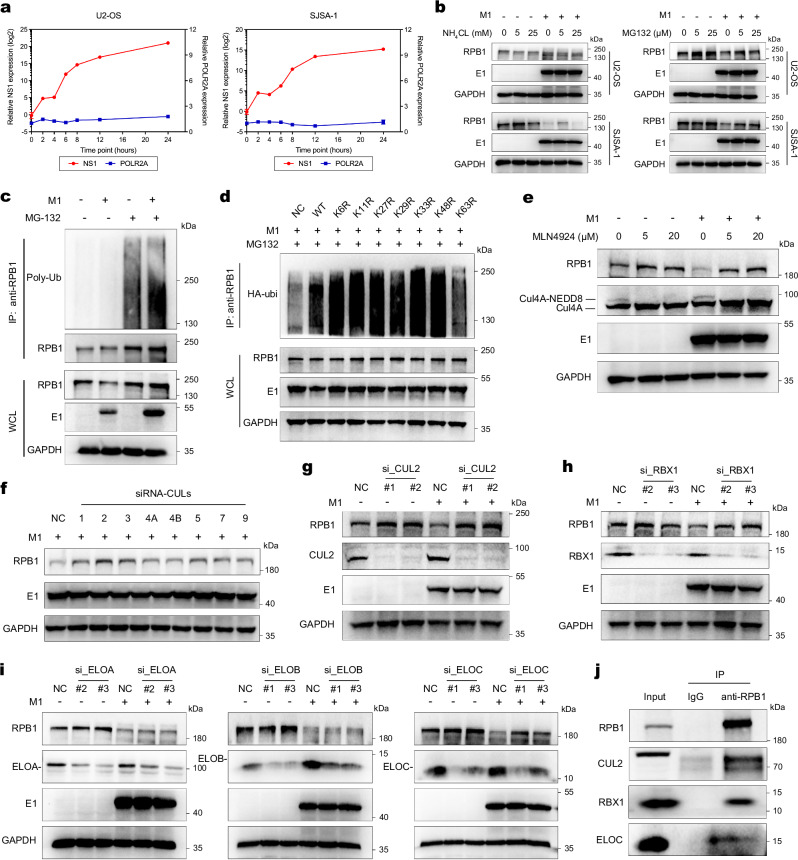
M1 virus induces the ubiquitination and degradation of RPB1 protein via the CUL2-RBX1-ELOC E3 ligase. **a** RT-qPCR analysis of *POLR2A* mRNA levels in U2-OS and SJSA-1 cells infected with M1 virus at different time points (NS1 served as an RNA marker for M1 virus infection). *n* = 3. **b** U2-OS and SJSA-1 cells infected with M1 virus were treated with MG-132 or NH_4_Cl, and western blot analysis was performed to assess RPB1 protein levels 12 h post-infection (E1 served as a protein marker for M1 virus infection). **c** U2-OS cells were treated with 1 MOI M1 virus or control solvent for 12 h, followed by treatment with or without MG-132 for 2 h. Cell lysates were subjected to immunoprecipitation (IP) with anti-RPB1, and ubiquitylated RPB1 proteins were detected using anti-ubiquitin Ab. **d** U2-OS cells transfected with HA-ubiquitin (wild-type or K6R, K11R, K27R, K29R, K33R, K48R, and K63R mutants) were treated with M1 virus and MG-132. Cell lysates were subjected to IP with anti-RPB1, and the ubiquitination of RPB1 was detected using anti-HA Ab. **e** RPB1 ubiquitination was assessed in U2-OS cells treated with or without the neddylation inhibitor MLN4924 for 1 h, followed by M1 virus infection and 12 h of incubation. The absence of Cullin neddylation was confirmed (Cul4A). **f** U2-OS cells transfected with siRNA targeting indicated CUL family member or control vectors were infected with M1 virus for 12 h and analyzed via western blot. **g–i** U2-OS cells transfected with siRNA targeting CUL2 (**g**), RBX1 (**h**), ELOA/B/C (**i**), or control vectors were treated with or without M1 virus and analyzed via western blot. **j** Co-immunoprecipitation analysis of endogenous RPB1, CUL2, RBX1, and ELOC in osteosarcoma cells. U2-OS cell lysates were immunoprecipitated with anti-RPB1 or control IgG, followed by immunoblotting with the indicated Abs. All experimental data (**b–j**) were verified in at least three independent experiments. Source data are provided as a Source Data file.

To investigate the mechanism underlying M1 virus-induced RPB1 downregulation, we first examined the mRNA levels of the *POLR2A* gene after M1 infection and found no significant changes (Fig. 6a). Protein degradation in cells primarily occurs through two pathways: the autophagy-lysosome pathway and the ubiquitin-proteasome pathway^33^. To determine the pathway responsible for M1-induced RPB1 degradation, we treated cells with the lysosomal inhibitor NH_4_Cl and the proteasome inhibitor MG-132. The results showed that MG-132 effectively blocked RPB1 degradation induced by M1 virus, whereas NH_4_Cl had no such effect (Fig. 6b). Furthermore, immunoprecipitation (IP) assays revealed that ubiquitination of RPB1 protein was significantly enhanced in osteosarcoma cells following M1 virus infection (Fig. 6c). Ubiquitin contains seven lysine (Lys) residues: K6, K11, K27, K29, K33, K48, and K63, which mediate different types of ubiquitin chains^34,35^. By overexpressing constructs with lysine-to-arginine mutations at different sites (tagged with HA), we identified that M1 infection increased K63-linked ubiquitination of RPB1 protein (Fig. 6d).

Recent studies on other viruses have shown that viral proteins can reprogram E3 ligases in host cells^36,37^. To date, more than 600 E3 ligases are estimated to be expressed in human cells, with the cullin-RING ligase (CRL) subfamily representing the largest group^34^. To explore the molecular events mediating RPB1 ubiquitination, we inhibited CRLs using the neddylation inhibitor MLN4924^38^. The results demonstrated that MLN4924 treatment completely abolished M1 virus-induced RPB1 ubiquitination, indicating that CRLs mediate RPB1 ubiquitination (Fig. 6e). Cullin-RING E3 ligases are multi-subunit complexes consisting of a scaffold protein (cullin), a RING-containing catalytic component, and an adapter protein. In human cells, multiple cullin subtypes (e.g., CUL1, CUL2, CUL3, CUL4A, CUL4B, CUL5, and CUL9) serve as cores for distinct CRL subclasses^39^. To identify the specific cullin subtype responsible for M1-induced RPB1 degradation in osteosarcoma cells, we knocked down each cullin using siRNA. The results showed that knockdown of endogenous CUL2 significantly blocked RPB1 degradation induced by M1 virus (Fig. 6f,g and Supplementary Fig. 12a, b). Among the dozens of CRL2 complexes identified in mammals, RBX1 serves as the canonical RING component^40^. To test this hypothesis, we knocked down RBX1 using siRNA, which disrupted the CRL complex, and found that M1-induced RPB1 degradation was effectively inhibited (Fig. 6h and Supplementary Fig. 12c). Since Elongin family proteins are essential adapters of CRL2 E3 ligases, we knocked down all Elongin family members, including ELOA, ELOB and ELOC (Supplementary Fig. 12d)^41^. The results revealed that only knockdown of ELOC blocked further RPB1 degradation (Fig. 6i). Furthermore, co-IP experiments confirmed the interaction between RPB1 and the CRL2 complex components (RBX1, CUL2 and ELOC) (Fig. 6j). The above results indicate that M1 virus induces RPB1 protein degradation through K63-linked ubiquitination via CUL2-RBX1-ELOC E3 ligase in osteosarcoma cells.

### NSP2 recruits CRL2 complex for RPB1 degradation in OS cells

**Fig. 7:**
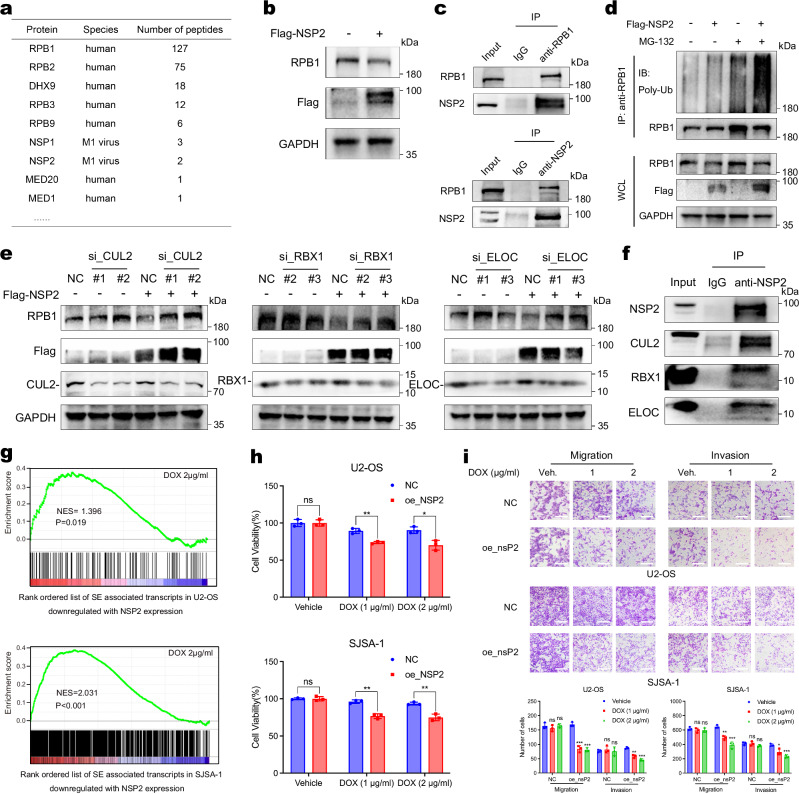
M1 virus non-structural protein NSP2 mediates anti-osteosarcoma activity by inducing RPB1 ubiquitination. **a** LC-MS/MS analysis of RPB1-associated proteins in M1 virus-infected U2-OS cells. Cell lysates from U2-OS cells infected with M1 virus were subjected to immunoprecipitation using an anti-RPB1 Ab. **b** U2-OS cells were transfected with plasmids expressing flag-tagged NSP2, and cell lysates were subjected to immunoblotting to detect alterations in RPB1 protein levels. **c** Co-immunoprecipitation of RPB1 and NSP2 in M1 virus infected U2-OS cells. Cell lysates were immunoprecipitated with anti-RPB1 or anti-NSP2. **d** U2-OS cells were transfected with plasmids expressing flag-tagged NSP2 or control vector, followed by treatment with or without MG-132 for 2 h. Cell lysates were subjected to immunoprecipitation with anti-RPB1 and then analyzed via western blot. **e** U2-OS cells transfected with siRNA targeting CUL2 (left), RBX1 (middle), ELOC (right), or control vectors were then transfected with or without Flag-NSP2 and analyzed via western blot. **f** Co-immunoprecipitation analysis of NSP2, CUL2, RBX1, and ELOC in M1 virus infected cells. Cell lysates were immunoprecipitated with anti-NSP2 or control IgG. **g** U2-OS cells transfected with DOX-inducible Tet-on NSP2 expression plasmid were treated with or without 2 μg/ml DOX, and transcriptional changes were analyzed by RNA sequencing. GSEA showing the enrichment of super-enhancer–associated transcript signature in NSP2 expressed cells versus control cells. P values were calculated by permutation testing. **h** MTT assay evaluating changes in cell viability of osteosarcoma cells transfected with DOX-inducible NSP2 expression plasmid or control vector, treated with 1 μg/ml, 2 μg/ml DOX, or vehicle. *n* = 3 biologically independent samples. The data are presented as the means ± SD and were analyzed by unpaired Student’s t test. *, *P* < 0.05; **, *P* < 0.01; ***, *P* < 0.001. **i** Migration and invasion abilities of osteosarcoma cells, treated as described for (**h**), were evaluated using the Transwell assay (upper). Scale bars, 150 μm. Quantification of cells in the Transwell assay is presented (lower). *n* = 3 biologically independent samples. All experimental data (**b–f**) were verified in at least three independent experiments. The data are presented as the means ± SD and were analyzed by one-way ANOVA. *, *P* < 0.05; **, *P* < 0.01; ***, *P* < 0.001. Source data are provided as a Source Data file.

To explore the mechanism underlying RPB1 ubiquitination induced by M1 virus infection, we employed Immunoprecipitation-Mass Spectrometry (IP-MS). In M1-infected host osteosarcoma cells, we identified that, in addition to interactions with other RNAPII subunits and reported transcription factors, the RPB1 protein also interacted with alphaviral non-structural proteins, NSP1 (non-structural protein 1) and NSP2 (non-structural protein 2) (Fig. 7a). Non-structural proteins of alphaviruses typically do not constitute viral particles but act as enzymes or auxiliary factors in viral replication and transcription. The biological impact of these non-structural proteins on the proteins in host tumor cells remains unclear^42–44^. We expressed NSP1, NSP2, NSP3 and NSP4 of M1 virus in osteosarcoma cells and found that only NSP2 induced a decrease in RPB1 protein levels, whereas other non-structural proteins, including NSP1, did not (Fig. 7b and Supplementary Fig. 13a).

Previous studies have reported that NSP2 is the only viral protein containing nuclear localization sequences (NLS1 and NLS2)^45,46^. As a key subunit of RNAPII, RPB1 predominantly localizes to the nucleus. Co-IP experiments further confirmed the interaction between NSP2 and RPB1 proteins (Fig. 7c). Consistent with changes observed during M1 infection, NSP2 increased the ubiquitination of RPB1 protein (Fig. 7d). To test the hypothesis that NSP2 mediates RPB1 degradation induced by M1 infection, we knocked down components of the CUL2-RBX1-ELOC E3 ligase complex in osteosarcoma cells expressing NSP2, and found that NSP2-induced degradation of RPB1 was blocked (Fig. 7e). Furthermore, the interaction between NSP2 and the CUL2-RBX1-ELOC complex was confirmed (Fig. 7f).

To further elucidate the role of the non-structural protein NSP2 in M1-mediated phenotypic and transcriptional effects, RNA-seq was performed on NSP2-expressing osteosarcoma cells (Supplementary Fig. 13b). The results showed selective downregulation of super-enhancer-associated gene transcription (Fig. 7g). Additionally, in vitro experiments revealed that NSP2 expression inhibited the proliferation and metastatic capabilities of osteosarcoma cells (Fig. 7h, i).

These findings indicate that non-structural protein NSP2 binds to host RPB1 protein during M1 virus infection and recruits the CUL2-RBX1-ELOC E3 ligase complex, promoting RPB1 protein ubiquitination and degradation. Therefore, NSP2 is a key viral factor in M1-mediated suppression of super-enhancer-associated gene expression, which inhibits the malignant characteristics of osteosarcoma cells.

## Discussion

Oncolytic virotherapy, which leverages engineered viruses to selectively infect tumor cells, has demonstrated significant therapeutic potential in various types of solid tumors^47^. In recent years, several oncolytic virus-based drugs have been approved or entered clinical trials, including T-VEC, H101, Reolysin, Pexa-Vec, DNX-2401, CG0070 and VSV-GP^48–51^. However, most studies on oncolytic virotherapy have focused on epithelial-origin tumors and hematologic malignancies, with limited reports in osteosarcoma or sarcomas. Unlike engineered viruses, M1 virus, a strain of Getah-like alphavirus, is a naturally occurring virus with oncolytic properties that was discovered serendipitously. Over the past decade, research has revealed its significant anti-tumor effects in multiple cancer types, providing fresh impetus for the development of oncolytic virotherapy^10,12,15,52^.

**Fig. 8:**
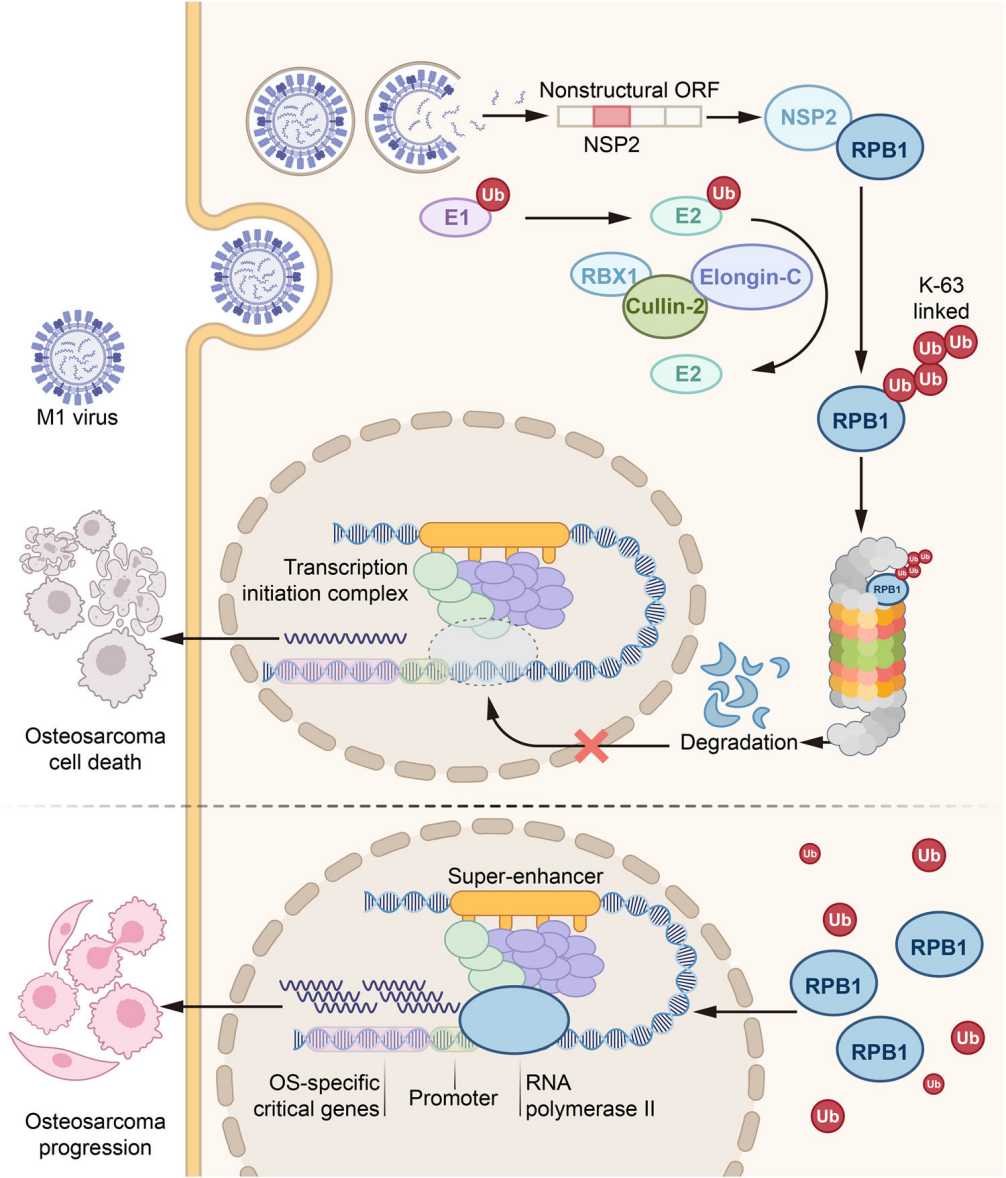
Mechanism by which M1 virus inhibits osteosarcoma progression via RPB1 degradation and super-enhancer suppression. Upon entry into osteosarcoma cells, the M1 virus expresses the non-structural protein NSP2, which directly binds to RPB1—a key component of the transcription initiation complex involved in RNA polymerase II-driven expression of super-enhancer–associated oncogenic genes. NSP2 recruits the Cullin-2 (CUL2)-RBX1-Elongin-C (ELOC) E3 ubiquitin ligase complex, leading to K63-linked ubiquitination of RPB1. This modification triggers proteasomal degradation of RPB1, thereby disrupting super-enhancer-mediated transcription of osteosarcoma-specific critical genes. The inhibition of this transcriptional machinery results in suppression of osteosarcoma cell proliferation and metastasis. In contrast, in the absence of M1 virus, super-enhancer activity remains intact, facilitating osteosarcoma progression.

In this study, we, for the first time, investigated the infectivity of M1 virus in osteosarcoma cells and reported its robust suppressive effects on growth and metastasis of osteosarcoma compared to other types of tumor. Interestingly, in osteosarcoma, the main anti-tumor mechanism of M1 virus was not the classical ER stress-induced apoptosis but rather the targeted inhibition of tumor-associated super-enhancer activity that drives osteosarcoma malignancy. Using multi-omics approaches, we examined the changes in osteosarcoma cells upon M1 virus infection and identified RPB1, a protein crucial for the transcriptional activating effects of tumor-associated super-enhancers, as a key target. Possibly due to varying degrees of dependency on RPB1 for super-enhancer activity among different osteosarcoma cells, suggesting that RPB1 expression could serve as a biomarker for predicting which subsets of osteosarcoma are sensitive or resistant to M1 virus treatment. And mechanistically, RPB1 was found to be degraded upon M1 virus infection. Specifically, M1 induced K63-linked ubiquitination of RPB1 in host osteosarcoma cells, leading to its proteasomal degradation. This process was mediated by viral non-structural protein NSP2, which binds to RPB1 and recruits the CUL2-RBX1-ELOC complex (Fig. 8).

Compared to other anti-tumor agents, oncolytic viruses offer several distinct advantages, including their non-pathogenic nature, relatively favorable safety profile, and the ability to selectively destroy tumor cells while sparing healthy cells^49^. In this study, we demonstrated that M1 virus selectively accumulates and replicates extensively in osteosarcoma lesions but not in normal organs. Furthermore, prolonged intravenous injection of M1 virus did not result in overt histopathological abnormalities in the examined organs. Mechanistically, our study revealed that the activity of super-enhancers that drive oncogene transcription in osteosarcoma is particularly sensitive to M1 virus infection and is significantly suppressed. The dual selectivity of M1 virus—targeting both tumor masses in vivo and oncogene transcription—provides supportive preclinical evidence for its activity in osteosarcoma. These findings suggest that M1 virus holds potential as a candidate therapeutic strategy or a delivery vector for osteosarcoma treatment in the future.

Alphaviruses are enveloped viruses with a capsid and spike protein structure encapsulating a single-stranded RNA (ssRNA) genome of positive polarity. Upon infecting host cells, the alphavirus ssRNA is directly released into the cytoplasm, allowing immediate translation without requiring nuclear transport. Through the highly efficient 26S subgenomic promoter, alphaviruses achieve robust viral protein expression^42^. Previous studies have reported the antitumor effects and mechanisms of alphaviruses such as Sindbis virus (SINV), Venezuelan equine encephalitis virus (VEEV) and M1 virus in glioblastoma, pancreatic cancer, liver cancer, melanoma, breast cancer, ovarian cancer, and leukemia^53^. In these types of tumor, alphaviruses, including M1 virus, were found to induce oncolysis primarily through their highly efficient RNA self-replication and high levels of viral protein expression, which activate mitochondrial apoptosis signaling pathways and trigger ER stress, ultimately leading to cell lysis. This process simultaneously releases tumor-associated antigens and viral RNA, further activating the host immune system and enhancing anti-tumor activity^54,55^. Previously, most studies on oncolytic alphavirus therapies have focused on epithelial tumors, hematopoietic and lymphoid tumors, neurogenic tumors and melanocytic tumors. However, there have been few reports on sarcomas, or even mesenchymal-origin tumors. This study is the first to reveal that M1 virus exerts anti-tumor effects in osteosarcoma primarily through an epigenetic mechanism by downregulating super-enhancer activity, thereby inhibiting oncogene transcription. This novel mechanism of oncolysis mediated by M1 virus offers a fresh perspective for research into oncolytic alphavirus therapies and suggests that different anti-tumor mechanisms of oncolytic alphaviruses may exist across diverse types of tumor.

Since the discovery in 2013 of super-enhancers’ pivotal role in regulating tumor malignancy, therapeutic strategies targeting super-enhancers have shown great promise for clinical translation across a wide range of tumor types^19^. Our previous work also identified lineage-specific super-enhancer elements that regulate numerous well-established oncogenes driving the initiation and progression of osteosarcoma, underscoring their potential as valuable therapeutic targets for osteosarcoma treatment^27,28,56^. Currently, therapeutic agents targeting super-enhancers primarily include BET family protein inhibitors or degraders and CDK inhibitors. Recently, inhibitors such as Pelabresib, ZEN-3694 and Samuraciclib have entered phase I/II/III clinical trials and demonstrated initial efficacy, particularly in hematological malignancies^57–59^. However, several challenges remain: (1) clinical toxicity, with common adverse effects including thrombocytopenia, diarrhea, fatigue, vomiting, anemia and hyperbilirubinemia; (2) lower or absent anti-tumor activity compared to that observed in preclinical studies for some inhibitors; and (3) drug resistance, leading to unsatisfactory clinical trial outcomes and even trial termination^60,61^. Unlike the toxicity and single-protein targeting commonly associated with small-molecule drugs, oncolytic viruses can specifically infect and destroy tumor cells through multiple pathways without damaging normal cells. Based on the unique mechanism of M1 virus in targeting tumor specific super-enhancers, which was discovered and established in this study, M1 virus represents a promising new strategy for super enhancers-targeting therapy.

The genome of the M1 virus is a messenger RNA consisting of 11,690 nucleotides with two open reading frames, which are translated and processed into nine proteins, including four non-structural proteins (NSP1, NSP2, NSP3 and NSP4) and five structural proteins (C, E3, E2, 6 K and E1)^62^. Previous studies have reported that the non-structural proteins (NSs) of viruses such as Rift Valley fever virus, Bunyamwera virus, La Crosse virus and some alphaviruses can inhibit host nuclear transcription factors, the Mediator complex and RNA polymerase II, although the specific mechanisms remain unclear^63–65^. The non-structural protein NSP2 is a catalytic enzyme in viral RNA replication, possessing helicase activity that unwinds secondary RNA structures and facilitates the RNA replication process. Previous studies have also demonstrated that NSP2 exhibits protease activity, which is responsible for cleaving non-structural polypeptides into mature functional non-structural proteins and regulate viral replication^46^. In this study, we, for the first time, identified the role of NSP2 in inducing the degradation of the RNA polymerase II functional subunit RPB1 protein. Previous studies have not reported that NSP2 possesses a ubiquitin ligase domain or associated activity, suggesting that NSP2 does not directly catalyze RPB1 ubiquitination. Recently, Li et al. found that the non-structural protein NSs of Rift Valley fever virus interacts with cellular FBXO3 to form a remodeled E3 ligase, triggering the degradation of the TFIIH complex^36^. Similarly, Ren et al. discovered that the NS5 protein of ZIKA virus (ZIKV) enhances the interaction between STAT2 and the ZSWIM8-CUL3 E3 ligase complex, facilitating STAT2 ubiquitination and suppressing type I interferon (IFN)-mediated antiviral immunity^37^. In this study, we demonstrated that the non-structural protein NSP2 of M1 virus interacts with cellular RPB1 and induces its ubiquitination and proteasome-dependent degradation by recruiting the CUL2-RBX1-ELOC E3 ligase complex.

In 2022, M1 virus was granted orphan drug designation by the FDA for the treatment of liver cancer and malignant glioma, and it has entered phase I clinical trials (NCT06368921, NCT06046742). This study provides the first mechanistic evidence that M1/NSP2 disrupts RPB1-dependent super-enhancer activity in osteosarcoma, together with supportive preclinical observations of antitumor activity. Several limitations should be noted. The in vivo evaluation was limited to one human osteosarcoma xenograft model and one syngeneic murine model, and thus does not fully establish the robustness or generalizability of the observed antitumor effects. In addition, the in vivo activity was demonstrated under an intensive daily intravenous dosing schedule, without systematic assessment of alternative regimens or intratumoral viral kinetics. Although the syngeneic model provides initial support for activity in an immunocompetent setting, the contribution of antiviral and antitumor immunity was not dissected in depth. Moreover, not all osteosarcoma cell lines were sensitive to M1 infection, indicating that response heterogeneity will need to be better defined in future studies. As a naturally occurring virus at an early stage of development, M1 virus holds significant potential for further engineering and sensitizing in cancer therapy. For instance, the valosin-containing protein (VCP) inhibitor has been identified to selectively enhance the potency of oncolytic viruses by up to 3600-fold through cooperation with M1 virus-suppressed IRE1α-XBP1 signaling pathways^16^. The mechanistic insights provided here support further preclinical evaluation and optimization of M1-based approaches.

## Methods

### Cell culture and viruses

Human osteosarcoma cell lines, including U2-OS, SJSA-1, HOS, 143B, MNNG/HOS, G-292, and MG-63 were obtained from the American Type Culture Collection (ATCC). Methotrexate-resistant U2OS/MTX300 cells, a derivative of the U2-OS osteosarcoma cell line, were generously provided by Dr. M. Serra (Istituti Ortopedici Rizzoli, Bologna, Italy). The ZOS and ZOS-M cell lines, derived from a primary osteosarcoma tumor and its metastasis, respectively, have been described previously^66^. Additionally, the ZOSL-1 cell line, established in our laboratory, was derived from a pulmonary metastasis of osteosarcoma^67^. K7M2, BHK-21, Vero, and HEK-293T cells were obtained from Cell Bank, Chinese Academy of Sciences. All cell lines were authenticated prior to use and cultured according to the ATCC’s guidelines.

The M1 virus was provided by Guangzhou Virotech Pharmaceutical Co., Ltd, and its genomic sequence is provided in Supplementary Table 2. M1 virus was produced in Vero cells, which were cultured in VP-SFM (Gibco) supplemented with 10% (vol/vol) MEM-NEAA and GlutaMAX (Thermo Scientific). When approximately 80% of the Vero cells were infected and showed a marked CPE, the supernatant was collected, centrifuged at 2000 × g and 4 °C for 10 min, and stored at−80 °C. In this study, recombinant derivatives were also utilized: M1-GFP, expressing jellyfish green fluorescent protein, and M1-iRFP, expressing the near-infrared fluorescent protein iRFP702. Virus titer: BHK-21 cells were seeded in 96-well plates at 3000 cells per well and incubated for 24 h. The virus-containing supernatant was serially diluted in DMEM and was then added to the BHK-21 cells. Seventy-two hours after infection, GFP expression and the CPE were evaluated under a uorescence microscope. The virus titer was calculated using Spearman-Karber method and converted to PFU. MOI (Multiplicity of Infection) was calculated using the formula: MOI = PFU (virus titer)×Volume of virus (mL)÷Number of target cells. The M1 variant used in this study has been characterized previously^68^.

### Animal experiments

All animal studies were approved by the Institutional Review Board of The First Affiliated Hospital of Sun Yat-sen University and conducted in compliance with established guidelines for the Care and Use of Laboratory Animals (Project Approval No. 2019-022). Female BALB/c nude mice (4–6 weeks old) were anesthetized with isoflurane, and SJSA-1 cells, either wild-type or transfected with plasmids, were injected into the proximal tibia through the cortex of the anterior tuberosity using a 30-gauge needle. For immunocompetent models, murine K7M2 cells were used to establish tumors in 4-week-old female BALB/c mice following the same orthotopic injection procedure. A total of 20 µL of cell suspension was slowly administered into the injection site.

For the M1 biodistribution study, 4-week-old female BALB/c nude mice were inoculated with 20 µL of wild-type SJSA-1 cells or PBS into the proximal tibia. Approximately three weeks later, non-tumor-bearing mice (PBS-injected) and tumor-bearing mice were each randomly divided into two groups (*n* = 3 per group), resulting in a total of four experimental groups. All mice received a single tail vein injection of either 300 µL of M1‑iRFP virus solution (4 × 10⁷ CCID_50_) or adjuvant solution (control). On day 4 post-injection, iRFP expression was assessed using an in vivo imaging system (IVIS). Subsequently, the three tumor-bearing mice that received M1 virus were euthanized, and tissue samples—including tumor, brain, heart, lungs, liver, kidneys, spleen, colon, lymph nodes, bone, and muscle—were collected for quantification of viral presence by RT‑qPCR.

For the in vivo antitumor efficacy study, experiments were conducted in both SJSA-1 xenograft models using BALB/c nude mice and K7M2 syngeneic models using immunocompetent BALB/c mice. Mice bearing tumors of approximately 200 mm³ in volume (established ~2 weeks post-inoculation) were randomly divided into two groups. The mice received either 300 µL of M1 virus solution (3.72 × 10⁷ CCID_50_) or adjuvant solution via tail vein injection once daily for 14 days. Tumor growth was monitored every 3 days over 3 weeks. Tumor dimensions were measured in two perpendicular cross-sectional widths (W1, W2), and the tumor volume was calculated using the formula: *V* = *W1* × *W2*^*2*^*/2*. At the end of the experiment, mice were sacrificed, and their lungs were harvested, fixed in Bouin’s solution, embedded in paraffin, sectioned (5 µm), and stained with H&E. The number of metastatic nodules in the lungs was counted. For safety assessment, mice body weights were recorded throughout the study. Vital tissues (brain, heart, liver, spleen, kidney, colon, skeletal muscle, and bone) were collected and histologically analyzed following H&E staining.

### Patients and clinical database

A retrospective analysis was conducted on osteosarcoma tissue specimens from 116 patients with complete follow-up data (81 males and 35 females; age range, 6–43 years; median age, 16 years), treated at the First Affiliated Hospital of Sun Yat-sen University between August 14, 2003, and May 12, 2010. Osteosarcoma diagnoses were confirmed prior to the study by pathologists in the hospital’s Clinical Pathology Department. All 116 patients were included in the overall survival (OS) analysis, while 106 patients without lung metastases at their initial visit were included in the lung metastasis-free survival (LMFS) analysis. Sex and gender were not considered as biological variables in the analysis because the study focused on cell-based and molecular mechanisms rather than sex-specific clinical outcomes. This study was approved by the Medical Ethics Committee of the First Affiliated Hospital of Sun Yat-sen University and conducted in compliance with the Declaration of Helsinki (Project Approval No. 2023-012). The requirement for informed consent was waived because this retrospective study used archived and did not involve additional intervention, risk, cost, or impact on patient treatment.

### Cell viability assay

Cells were seeded in 96-well plates at a density of 2000 cells per well in 0.1 mL of medium. For time-response assays, 1000 cells per well were plated. Following treatment, 3-(4,5-dimethylthiazol-2-yl)−2,5-diphenyltetrazolium bromide (MTT) was added to each well at a final concentration of 1 mg/mL, and the plates were incubated at 37 °C for 4 h. The medium was then replaced with 150 μL of DMSO and mixed thoroughly to dissolve the formazan crystals. Absorbance was measured at 490 nm using a microplate reader. All experiments were performed in triplicate.

### Clone formation assay

Osteosarcoma cells or plasmid-transfected cells were seeded in six-well plates at a density of 500 cells per well in 2 mL of DMEM supplemented with 10% FBS. Each condition was plated in triplicate. Following treatment, the cells were cultured for 14 days, with the medium refreshed every 3 days. Colonies containing more than 50 cells were stained with crystal violet and counted after 10 days. Data are presented as the mean ± SD from three independent experiments, each performed in triplicate.

### In vitro migration and invasion assays

Cell migration and invasion assays were conducted using Transwell chambers with 8-μm microporous filters, which were either uncoated or precoated with an extracellular matrix (BD Biosciences), following the manufacturer’s protocol. For the assays, 200 μL of cell suspension (5 × 10^4^ cells/mL) in serum-free DMEM was added to the upper chambers of 24-well plates, while 500 μL of DMEM containing 10% FBS was placed in the lower chambers as a chemoattractant. After 24 h, cells remaining on the upper surface of the filter were removed, and those that migrated to the lower surface were fixed, stained, and counted in three random fields under a light microscope. Data are presented as the mean ± SD from three independent experiments, each performed in triplicate.

### Transmission electron microscopy

Osteosarcoma cells were infected with M1 virus at a multiplicity of infection (MOI) of 1 for the indicated durations. Cells were harvested by centrifugation at 1000 × g for 5 min at room temperature. The resulting cell pellets were resuspended, washed once with phosphate-buffered saline (PBS), and centrifuged again at 1500 × g for 5 min. The pellets were then fixed on ice for 4 h in 0.1 M PBS (pH 7.4) containing 2.5% glutaraldehyde and 2% paraformaldehyde. Fixed samples were submitted to the Electron Microscopy Facility at Zhongshan School of Medicine, Sun Yat-sen University, for standard ultrastructural analysis using transmission electron microscopy.

### RNA-seq and GSEA

Total RNA was extracted from U2-OS and SJSA-1 cells infected with M1 virus (1 MOI) for 0, 6, 12, and 24 h or transfected with the Tet-on DOX-inducible NSP2 plasmid using TRIzol reagent (Invitrogen), following the manufacturer’s protocol. RNA purity, concentration, and integrity were assessed using NanoDrop, Qubit RNA BR Assay Kit, and RNA ScreenTape, respectively. For library preparation, 0.1–1 μg of total RNA was used to isolate mRNA with the NEBNext® Poly(A) mRNA Magnetic Isolation Module, followed by library construction using the NEBNext® Ultra™ II mRNA Library Prep Kit for Illumina®. Library quality and concentration were evaluated with the Qubit™ dsDNA HS Assay Kit, D1000 ScreenTape, and KAPA Library Quant Kit. Sequencing was performed on a NovaSeq 6000 platform. Raw reads in FASTQ format were processed with fastp for quality control, adapter trimming, and filtering. High-quality clean reads were aligned to the reference genome using HISAT2 v2.1.0. Gene expression levels were quantified with FeatureCounts, and FPKM values were calculated based on gene length and mapped read counts.

Gene Set Enrichment Analysis (GSEA) was performed using the GSEA software from the Broad Institute (https://www.gsea-msigdb.org/gsea). The analysis was conducted using gene sets from the Molecular Signatures Database (MSigDB) as well as super-enhancer-associated genes specific to each cell line.

### Chromatin immunoprecipitation, sequencing, and analysis

ChIP assays were conducted following the manufacturer’s protocol (Millipore, Danvers, MA, USA). Briefly, M1 virus-infected or uninfected SJSA-1 cells were cultured at 37 °C in 5% CO2 and cross-linked with 1% formaldehyde at room temperature for 10 minutes, followed by neutralization with glycine. The cells were resuspended, lysed in lysis buffer, and sonicated on ice to shear the DNA into fragments of 200–750 bp. Magnetic beads were pre-bound with 10 µg of the specified antibodies (anti-H3K27ac, #ab4729, Abcam, or anti-RPB1, #ab26271, Abcam). For immunoprecipitation, the sonicated chromatin was incubated overnight at 4 °C with antibody-bound magnetic beads to enrich for DNA fragments associated with the target proteins. Beads were washed three times with sonication buffer and twice with low-stringency wash buffer. DNA was eluted in elution buffer, and RNA and proteins were digested using RNase A (#70856, Millipore, USA) and Proteinase K, respectively. The DNA was purified using phenol-chloroform extraction followed by ethanol precipitation.

Illumina sequencing libraries were prepared according to the NEBNext® Ultra™ DNA Library Prep Kit protocol. Sequencing data were processed as described by Lin et al. ^69^. Reads passing quality filters were aligned to the human genome (hg38) using Bowtie2 (v2.4.2). Enriched regions (enhancers) were identified using MACS2 with a Q-value threshold of <0.1.

Super-enhancers were determined using the ROSE (Rank Ordering of Super-Enhancers) algorithm (https://bitbucket.org/young_computation/rose) as described in prior studies^29^. H3K27ac peaks within a 12.5 kb distance were merged, excluding those within 2 kb of a RefSeq TSS. Stitched domains were ranked by H3K27ac or RPB1 signal, and super-enhancers were distinguished from typical enhancers by identifying the inflection point on the ranking curve, where the slope equals one. Each enhancer was assigned to the nearest transcript based on the proximity of its center to a RefSeq TSS.

### TMT LC-MS/MS and analysis

Cell lysis and protein extraction were performed using SDT buffer (4% SDS, 100 mM Tris-HCl, 1 mM DTT, pH 7.6) on cells treated with 1 MOI M1 virus or adjuvant solution for 12 h. Protein concentration was measured using the BCA Protein Assay Kit (Bio-Rad). Proteins were digested using the filter-aided sample preparation (FASP) method, and peptides were desalted on C18 cartridges, vacuum-concentrated, and reconstituted in 0.1% formic acid. Peptides (100 µg per sample) were labeled with TMT reagents (Thermo Scientific) following the manufacturer’s protocol. Labeled peptides were fractionated via strong cation exchange (SCX) chromatography using an AKTA Purifier system (GE Healthcare) with a gradient of buffer B (500 mM KCl in 25% ACN). Fractions were desalted on C18 cartridges and reconstituted for LC-MS/MS analysis. Peptides were separated on an Easy-nLC system (Thermo Scientific) using a trap column and an analytical column with a flow rate of 300 nL/min. The eluted peptides were analyzed on a Q-Exactive mass spectrometer in data-dependent acquisition mode. Full MS scans were acquired at 70,000 resolution, followed by MS/MS analysis of the top 20 most intense ions at 17,500 resolution. Raw MS data were analyzed using the MASCOT engine (Matrix Science) integrated with Proteome Discoverer 1.4 for protein identification and quantification.

### Immunohistochemical staining, evaluation, and analysis

Formalin-fixed, paraffin-embedded tissue sections were stained with anti-RPB1 (#ab26721, Abcam) at 4 °C overnight, followed by incubation with the Dako EnVision secondary antibody (Dako, Glostrup, Denmark) for 30 min at room temperature. The staining intensity and proportion of positive cells were independently assessed and scored by two pathologists blinded to the patient data. Staining intensity was graded as follows: 0 = no staining, 1 = weak, 2 = moderate, and 3 = strong. The extent of positive staining was scored as follows: 0 = no positive staining, 1 = 1–25% of cells, 2 = 26–50% of cells, 3 = 51–75% of cells, and 4 = 76–100% of cells. The final score, ranging from 0 to 12, was calculated as the product of the intensity and extent scores. Low, medium, and high expression levels were determined using the X-tile software^70^, based on the final scores. For statistical analysis, RPB1 expression levels were categorized into low (score 0–3), medium (score 4–8), and high (score 9–12). These categories were used to analyze overall survival (OS) and lung metastasis-free survival (LMFS).

### Cell transfection

Short hairpin RNA (shRNA) sequences targeting *POLR2A* were obtained from GeneCopoeia. Three shRNA sequences were designed and cloned into the psi-LVRU6GP-puro vector, with a scrambled shRNA serving as a negative control. The most effective shRNA sequences were *POLR2A* shRNA #2 (GCATTGACTTGCGTTTCCACC) and #3 (GGGACATTGTTATCTTCAACC).

To overexpress *POLR2A*, the full-length coding sequence was cloned into the pEZ-Lv157 lentiviral expression vector (GeneCopoeia) with a C-terminal 3×HA tag. For the overexpression of M1 viral genes NS1–4, their full-length coding sequences were individually cloned into the pEZ-Lv206 lentiviral expression vector (GeneCopoeia) with a C-terminal 3×FLAG tag. To achieve inducible overexpression of NSP2 with a 3×FLAG tag, the coding sequence was cloned into the Tet-on inducible expression vector GL912 (Obio Technology, China), with the 3×FLAG tag fused at the C-terminus. The construct was verified by DNA sequencing to confirm correct insertion and tag orientation. Plasmids encoding 3×HA-tagged ubiquitin mutants with lysine-to-arginine (K-to-R) substitutions at positions K6, K11, K27, K29, K33, K48, and K63 were kindly provided by Dr. Shanyu Huang (Zhongshan School of Medicine, Guangzhou, China).

Recombinant lentiviruses were generated and used to infect target cells in the presence of 8 µg/mL polybrene following the manufacturer’s protocol. Stable cell lines were selected using 1 µg/mL puromycin for 2 days, during which uninfected cells were completely eliminated. Protein samples for Western blot analysis were collected 1 week post-selection. For cells transfected with the Tet-on system, inducible expression of 3×FLAG-tagged NSP2 was initiated by treating cells with doxycycline (DOX; Selleck Chemicals) at the indicated concentrations.

Specific and non-targeting siRNAs were purchased from Ribobio. Cells were cultured in DMEM supplemented with 10% fetal bovine serum (without penicillin/streptomycin). Transfection was performed using Lipofectamine RNAiMAX (Thermo Fisher, #13778-150) in OPTI-MEM (Thermo Fisher, #31985070) following the manufacturer’s instructions.

### RNA extraction and RT-qPCR

Total RNA was extracted using TRIzol reagent (Invitrogen) following the manufacturer’s protocol. Reverse transcription was conducted with the PrimeScript RT Reagent Kit (Takara). Quantitative PCR (qPCR) was performed using SYBR Premix (Takara) on an Applied Biosystems 7500 Fast Real-Time PCR System (Life Technologies). All reactions, including no-reverse-transcription and no-template controls, were conducted in triplicate in a 20 µL reaction volume. Primer sequences were obtained from PrimerBank (http://pga.mgh.harvard.edu/primerbank/). The expression levels of target gene mRNA were normalized to GAPDH. M1 RNA concentrations were expressed as copy numbers per microgram of RNA. The RT-qPCR primer sequences used are listed in materials detailed information.

### Western blotting

Cells were lysed using M-PER Mammalian Protein Extraction Reagent (Thermo Scientific) supplemented with protease and phosphatase inhibitor cocktails (Roche). Cell debris was removed by centrifugation at 12,000 × g for 10 min. Equal amounts of protein were boiled for 10 minutes, separated by SDS-PAGE, and transferred onto polyvinylidene difluoride (PVDF) membranes (Millipore). Membranes were blocked with 5% nonfat dry milk in TBST for 1 h at room temperature, followed by overnight incubation at 4 °C with primary antibodies. After three washes with TBST, the membranes were incubated with the appropriate secondary antibodies. Immunoreactive proteins were visualized using a ChemiDoc XRS+ System (Bio-Rad) and ECL detection reagents (Millipore).

### Flow cytometry

Osteosarcoma cells (U2-OS and SJSA-1) transfected with either the *POLR2A* overexpression plasmid or a control vector were treated with M1-GFP at a multiplicity of infection (MOI) of 1 for 0, 6, 12, and 24 h. Following treatment, cells were trypsinized and permeabilized using the CytoFix/CytoPerm kit (BD Biosciences) according to the manufacturer’s instructions. Flow cytometry analysis was conducted using a Flow Cytometer (CytoFLEX, Beckman Colter), and data were processed and analyzed with CytExpert software.

### Immunoprecipitation and ubiquitination analyses

U2-OS cell samples, either untreated or treated with M1 virus, plasmid transfection, or the MG-132 inhibitor as indicated, were lysed in ice-cold immunoprecipitation (IP) buffer (#P0013, Beyotime) containing protease and phosphatase inhibitor cocktails. Insoluble materials were removed by centrifugation. The clarified lysates were incubated overnight at 4 °C with BeyoMag™ Protein A + G magnetic beads (#P2108, Beyotime) pre-coated with anti-RPB1 or anti-NSP2 antibodies. The beads were washed multiple times with 1× TBS using a magnetic rack and then resuspended in 1× SDS-PAGE loading buffer. After heating at 95 °C for 5 min, the beads were placed on the magnetic rack to separate the supernatant, which was collected as the final sample for subsequent analysis. Ubiquitinated RPB1 was detected by immunoblotting (IB) using anti-ubiquitin and anti-HA antibodies (for HA-tagged ubiquitin mutants).

### Statistics and reproducibility

Statistical analyses were conducted using Prism 8.0 (GraphPad), SPSS version 26.0 (SPSS Inc., Chicago, IL, USA), and R version 4.1.3 (The R Foundation for Statistical Computing). Data were obtained from at least three independent experiments and are presented as the mean ± SD. Statistical details of experiments can be found in figure legends. Differences between groups were analyzed using Student’s *t*-test, one-way ANOVA, or two-way ANOVA, as appropriate. Kaplan-Meier survival analyses were performed using the log-rank test, and Pearson correlation was used for correlation analyses. A *P* value < 0.05 was considered statistically significant. In all cases, the *p* values are represented as follows: *p < 0.05, **p < 0.01, ***p < 0.001 and ****p < 0.0001.

### Reporting summary

Further information on research design is available in the Nature Portfolio Reporting Summary linked to this article.

## Supplementary information

Supplementary information

Reporting Summary

Transparent Peer Review file

## Source data

Source Data

## Data Availability

The data supporting the findings of this study are available within the Article, Supplementary Information and Source Data file. Source data are provided with this paper. The ChIP-seq data of osteosarcoma cells pre- and post-M1 virus infection, using anti-H3K27ac and anti-RPB1 antibodies, have been deposited at GEO (GSE293855) [https://www.ncbi.nlm.nih.gov/geo/query/acc.cgi?acc=GSE293855]. RNA-seq data of osteosarcoma cells pre- and post-M1 virus infection, as well as pre- and post-plasmid transfection, have been deposited at GEO (GSE293856) [https://www.ncbi.nlm.nih.gov/geo/query/acc.cgi?acc=GSE293856]. The proteomics data, including TMT-based quantitative proteomics and IP-MS data, have been deposited to the ProteomeXchange Consortium under accession code PXD065368. Source data are provided with this paper.

## References

[1] Isakoff, M. S., Bielack, S. S., Meltzer, P. & Gorlick, R. Osteosarcoma: current treatment and a collaborative pathway to success. *J. Clin. Oncol.* **33**, 3029–3035 (2015). 26304877 10.1200/JCO.2014.59.4895 PMC4979196

[2] Beird, H. C. et al. Osteosarcoma. *Nat. Rev. Dis. Prim.* **8**, 77 (2022). 36481668 10.1038/s41572-022-00409-y

[3] Siegel, R. L., Miller, K. D. & Jemal, A. Cancer statistics, 2019. *CA Cancer J. Clin.* **69**, 7–34 (2019). 30620402 10.3322/caac.21551

[4] Gill, J. & Gorlick, R. Advancing therapy for osteosarcoma. *Nat. Rev. Clin. Oncol.* **18**, 609–624 (2021). 34131316 10.1038/s41571-021-00519-8

[5] Lawler, S. E., Speranza, M. C., Cho, C. F. & Chiocca, E. A. Oncolytic viruses in cancer treatment: a review. *JAMA Oncol.* **3**, 841–849 (2017). 27441411 10.1001/jamaoncol.2016.2064

[6] Raja, J., Ludwig, J. M., Gettinger, S. N., Schalper, K. A. & Kim, H. S. Oncolytic virus immunotherapy: future prospects for oncology. *J. Immunother. Cancer* **6**, 140 (2018). 30514385 10.1186/s40425-018-0458-z PMC6280382

[7] Harrington, K., Freeman, D. J., Kelly, B., Harper, J. & Soria, J. C. Optimizing oncolytic virotherapy in cancer treatment. *Nat. Rev. Drug Discov.* **18**, 689–706 (2019). 31292532 10.1038/s41573-019-0029-0

[8] N. Macedo, D. M. Miller, R. Haq, H. L. Kaufman. (2020). Clinical landscape of oncolytic virus research in 2020. *J. Immunother. Cancer* **8**, 1486 (2020). 10.1136/jitc-2020-001486 PMC7552841 33046622

[9] Pol, J., Kroemer, G. & Galluzzi, L. First oncolytic virus approved for melanoma immunotherapy. *Oncoimmunology* **5**, e1115641 (2016). 26942095 10.1080/2162402X.2015.1115641 PMC4760283

[10] Guo, L. et al. Directed natural evolution generates a next-generation oncolytic virus with a high potency and safety profile. *Nat. Commun.* **14**, 3410 (2023). 37296165 10.1038/s41467-023-39156-3 PMC10256765

[11] Cai, J. & Yan, G. The identification and development of a novel oncolytic virus: alphavirus M1. *Hum. Gene Ther.* **32**, 138–149 (2021). 33261513 10.1089/hum.2020.271

[12] Lin, Y. et al. Identification and characterization of alphavirus M1 as a selective oncolytic virus targeting ZAP-defective human cancers. *Proc. Natl. Acad. Sci. USA* **111**, E4504–E4512 (2014). 25288727 10.1073/pnas.1408759111 PMC4210284

[13] Dan, J. et al. Visualization of the oncolytic alphavirus M1 life cycle in cancer cells. *Virol. Sin.* **36**, 655–666 (2021). 33481190 10.1007/s12250-020-00339-7 PMC8379316

[14] Dan, J. et al. Oncolytic virus M1 functions as a bifunctional checkpoint inhibitor to enhance the antitumor activity of DC vaccine. *Cell Rep. Med.* **4**, 101229 (2023). 37820722 10.1016/j.xcrm.2023.101229 PMC10591054

[15] Song, D. et al. Identification of the receptor of oncolytic virus M1 as a therapeutic predictor for multiple solid tumors. *Signal Transduct. Target. Ther.* **7**, 100 (2022). 35393389 10.1038/s41392-022-00921-3 PMC8989880

[16] H. Zhang, et al. Targeting VCP enhances anticancer activity of oncolytic virus M1 in hepatocellular carcinoma. *Sci. Transl. Med.* **9**, 7996 (2017). 10.1126/scitranslmed.aam7996 28835517

[17] Sun, S. et al. Combining NanoKnife with M1 oncolytic virus enhances anticancer activity in pancreatic cancer. *Cancer Lett.* **502**, 9–24 (2021). 33444691 10.1016/j.canlet.2020.12.018

[18] Zhai, X. et al. LDLR is used as a cell entry receptor by multiple alphaviruses. *Nat. Commun.* **15**, 622 (2024). 38245515 10.1038/s41467-024-44872-5 PMC10799924

[19] Liu, S. et al. Effects of super-enhancers in cancer metastasis: mechanisms and therapeutic targets. *Mol. Cancer* **23**, 122 (2024). 38844984 10.1186/s12943-024-02033-8 PMC11157854

[20] Lovén, J. et al. Selective inhibition of tumor oncogenes by disruption of super-enhancers. *Cell* **153**, 320–334 (2013). 23582323 10.1016/j.cell.2013.03.036 PMC3760967

[21] Jiang, J., Wang, W., Xiang, W., Jiang, L. & Zhou, Q. The phosphoinositide 3-kinase inhibitor ZSTK474 increases the susceptibility of osteosarcoma cells to oncolytic vesicular stomatitis virus VSVΔ51 via aggravating endoplasmic reticulum stress. *Bioengineered* **12**, 11847–11857 (2021). 34720036 10.1080/21655979.2021.1999372 PMC8809975

[22] Jia, X., Chen, Y., Zhao, X., Lv, C. & Yan, J. Oncolytic vaccinia virus inhibits human hepatocellular carcinoma MHCC97-H cell proliferation via endoplasmic reticulum stress, autophagy and Wnt pathways. *J. Gene Med.* **18**, 211–219 (2016). 27441866 10.1002/jgm.2893

[23] Cui, B. et al. Non-small cell lung cancers (NSCLCs) oncolysis using coxsackievirus B5 and synergistic DNA-damage response inhibitors. *Signal Transduct. Target. Ther.* **8**, 366 (2023). 37743418 10.1038/s41392-023-01603-4 PMC10518312

[24] Wang, G. et al. The potential of swine pseudorabies virus attenuated vaccine for oncolytic therapy against malignant tumors. *J. Exp. Clin. Cancer Res.* **42**, 284 (2023). 37891570 10.1186/s13046-023-02848-1 PMC10604416

[25] Lu, B. et al. Pharmacological inhibition of core regulatory circuitry liquid-liquid phase separation suppresses metastasis and chemoresistance in osteosarcoma. *Adv. Sci.* **8**, e2101895 (2021). 10.1002/advs.202101895 PMC8529446 34432948

[26] Ouyang, Z. et al. Construction of a five-super-enhancer-associated-genes prognostic model for osteosarcoma patients. *Front. Cell. Dev. Biol.* **8**, 598660 (2020). 33195283 10.3389/fcell.2020.598660 PMC7661850

[27] Zhang, J. et al. Targeting super-enhancer-associated oncogenes in osteosarcoma with THZ2, a covalent CDK7 inhibitor. *Clin. Cancer Res.* **26**, 2681–2692 (2020). 31937612 10.1158/1078-0432.CCR-19-1418

[28] Huang, G. et al. SNPs give LACTB oncogene-like functions and prompt tumor progression via dual-regulating p53. *Adv. Sci.* **11**, e2405907 (2024). 10.1002/advs.202405907 PMC11578386 39324579

[29] Whyte, W. A. et al. Master transcription factors and mediator establish super-enhancers at key cell identity genes. *Cell* **153**, 307–319 (2013). 23582322 10.1016/j.cell.2013.03.035 PMC3653129

[30] Hnisz, D. et al. Super-enhancers in the control of cell identity and disease. *Cell* **155**, 934–947 (2013). 24119843 10.1016/j.cell.2013.09.053 PMC3841062

[31] Tang, F., Yang, Z., Tan, Y. & Li, Y. Super-enhancer function and its application in cancer targeted therapy. *npj Precis. Oncol.* **4**, 2 (2020). 32128448 10.1038/s41698-020-0108-z PMC7016125

[32] Lao, L. et al. ARMC5 is part of an RPB1-specific ubiquitin ligase implicated in adrenal hyperplasia. *Nucleic Acids Res* **50**, 6343–6367 (2022). 35687106 10.1093/nar/gkac483 PMC9226510

[33] Rusilowicz-Jones, E. V., Urbé, S. & Clague, M. J. Protein degradation on the global scale. *Mol. Cell* **82**, 1414–1423 (2022). 35305310 10.1016/j.molcel.2022.02.027

[34] Beck, D. B., Werner, A., Kastner, D. L. & Aksentijevich, I. Disorders of ubiquitylation: unchained inflammation. *Nat. Rev. Rheumatol.* **18**, 435–447 (2022). 35523963 10.1038/s41584-022-00778-4 PMC9075716

[35] van Huizen, M. & Kikkert, M. The role of atypical ubiquitin chains in the regulation of the antiviral innate immune response. *Front. Cell. Dev. Biol.* **7**, 392 (2019). 32039206 10.3389/fcell.2019.00392 PMC6987411

[36] Li, H. et al. Rift valley fever virus coordinates the assembly of a programmable E3 ligase to promote viral replication. *Cell* **187**, 6896–6913.e15 (2024). 39366381 10.1016/j.cell.2024.09.008

[37] Ren, W. et al. Zika virus NS5 protein inhibits type I interferon signaling via CRL3 E3 ubiquitin ligase-mediated degradation of STAT2. *Proc. Natl. Acad. Sci. USA* **121**, e2403235121 (2024). 39145933 10.1073/pnas.2403235121 PMC11348293

[38] Soucy, T. A. et al. An inhibitor of NEDD8-activating enzyme as a new approach to treat cancer. *Nature* **458**, 732–736 (2009). 19360080 10.1038/nature07884

[39] Harper, J. W. & Schulman, B. A. Cullin-RING ubiquitin ligase regulatory circuits: a quarter century beyond the F-box hypothesis. *Annu. Rev. Biochem.* **90**, 403–429 (2021). 33823649 10.1146/annurev-biochem-090120-013613 PMC8217159

[40] Cardote, T. A. F., Gadd, M. S. & Ciulli, A. Crystal structure of the Cul2-Rbx1-EloBC-VHL ubiquitin ligase complex. *Structure* **25**, 901–911.e3 (2017). 28591624 10.1016/j.str.2017.04.009 PMC5462531

[41] Zhao, Y., Xiong, X. & Sun, Y. Cullin-RING Ligase 5: functional characterization and its role in human cancers. *Semin. Cancer Biol.* **67**, 61–79 (2020). 32334051 10.1016/j.semcancer.2020.04.003

[42] Skidmore, A. M. & Bradfute, S. B. The life cycle of the alphaviruses: from an antiviral perspective. *Antivir. Res* **209**, 105476 (2023). 36436722 10.1016/j.antiviral.2022.105476 PMC9840710

[43] Ahola, T., McInerney, G. & Merits, A. Alphavirus RNA replication in vertebrate cells. *Adv. Virus Res.* **111**, 111–156 (2021). 34663497 10.1016/bs.aivir.2021.07.003

[44] Lundstrom K. Self-replicating alphaviruses: from pathogens to therapeutic agents. *Viruses* **16**, 1762 (2024). 10.3390/v16111762 PMC11598883 39599876

[45] Russo, A. T., White, M. A. & Watowich, S. J. The crystal structure of the venezuelan equine encephalitis alphavirus nsP2 protease. *Structure* **14**, 1449–1458 (2006). 16962975 10.1016/j.str.2006.07.010

[46] Rupp, J. C., Sokoloski, K. J., Gebhart, N. N. & Hardy, R. W. Alphavirus RNA synthesis and non-structural protein functions. *J. Gen. Virol.* **96**, 2483–2500 (2015). 26219641 10.1099/jgv.0.000249 PMC4635493

[47] Ma, R., Li, Z., Chiocca, E. A., Caligiuri, M. A. & Yu, J. The emerging field of oncolytic virus-based cancer immunotherapy. *Trends Cancer* **9**, 122–139 (2023). 36402738 10.1016/j.trecan.2022.10.003 PMC9877109

[48] Cripe, T. P. et al. Phase 1 study of intratumoral Pexa-Vec (JX-594), an oncolytic and immunotherapeutic vaccinia virus, in pediatric cancer patients. *Mol. Ther.* **23**, 602–608 (2015). 25531693 10.1038/mt.2014.243 PMC4351466

[49] Lin, D., Shen, Y. & Liang, T. Oncolytic virotherapy: basic principles, recent advances and future directions. *Signal Transduct. Target Ther.* **8**, 156 (2023). 37041165 10.1038/s41392-023-01407-6 PMC10090134

[50] Gállego Pérez-Larraya, J. et al. Oncolytic DNX-2401 virus for pediatric diffuse intrinsic pontine glioma. *N. Engl. J. Med.* **386**, 2471–2481 (2022). 35767439 10.1056/NEJMoa2202028

[51] Das, K. et al. A modular self-adjuvanting cancer vaccine combined with an oncolytic vaccine induces potent antitumor immunity. *Nat. Commun.* **12**, 5195 (2021). 34465781 10.1038/s41467-021-25506-6 PMC8408233

[52] Cai, J. et al. Selective replication of oncolytic virus M1 results in a bystander killing effect that is potentiated by Smac mimetics. *Proc. Natl. Acad. Sci. Usa.* **114**, 6812–6817 (2017). 28607091 10.1073/pnas.1701002114 PMC5495246

[53] Lundstrom, K. Alphaviruses in cancer therapy. *Front. Mol. Biosci.* **9**, 864781 (2022). 35495626 10.3389/fmolb.2022.864781 PMC9046730

[54] Pampeno, C., Opp, S., Hurtado, A., Meruelo, D. Sindbis virus vaccine platform: a promising oncolytic virus-mediated approach for ovarian cancer treatment. *Int. J. Mol. Sci.* **25**, 2925 (2024). 10.3390/ijms25052925 PMC10932354 38474178

[55] Aliahmad, P., Miyake-Stoner, S. J., Geall, A. J. & Wang, N. S. Next generation self-replicating RNA vectors for vaccines and immunotherapies. *Cancer Gene Ther.* **30**, 785–793 (2023). 35194198 10.1038/s41417-022-00435-8 PMC8861484

[56] Huang, G. et al. Prognostic and predictive value of super-enhancer-derived signatures for survival and lung metastasis in osteosarcoma. *J. Transl. Med.* **22**, 88 (2024). 38254188 10.1186/s12967-024-04902-8 PMC10801997

[57] Blum, K. A. et al. A phase I study of pelabresib (CPI-0610), a small-molecule inhibitor of BET proteins, in patients with relapsed or refractory lymphoma. *Cancer Res. Commun.* **2**, 795–805 (2022). 36923307 10.1158/2767-9764.CRC-22-0060 PMC10010313

[58] Kharenko, O. A., Patel, R. G., Calosing, C. & van der Horst, E. H. Combination of ZEN-3694 with CDK4/6 inhibitors reverses acquired resistance to CDK4/6 inhibitors in ER-positive breast cancer. *Cancer Gene Ther.* **29**, 859–869 (2022). 34385584 10.1038/s41417-021-00375-9

[59] Coombes, R. C. et al. Dose escalation and expansion cohorts in patients with advanced breast cancer in a phase I study of the CDK7-inhibitor samuraciclib. *Nat. Commun.* **14**, 4444 (2023). 37488191 10.1038/s41467-023-40061-y PMC10366102

[60] Tang, P., Zhang, J., Liu, J., Chiang, C. M. & Ouyang, L. Targeting bromodomain and extraterminal proteins for drug discovery: from current progress to technological development. *J. Med. Chem.* **64**, 2419–2435 (2021). 33616410 10.1021/acs.jmedchem.0c01487 PMC8106541

[61] Hill, M. D. et al. Development of BET inhibitors as potential treatments for cancer: a search for structural diversity. *Bioorg. Med. Chem. Lett.* **44**, 128108 (2021). 33991625 10.1016/j.bmcl.2021.128108

[62] Wen, J. S. et al. Genomic analysis of a Chinese isolate of getah-like virus and its phylogenetic relationship with other alphaviruses. *Virus Genes* **35**, 597–603 (2007). 17570048 10.1007/s11262-007-0110-3

[63] Billecocq, A. et al. NSs protein of rift valley fever virus blocks interferon production by inhibiting host gene transcription. *J. Virol.* **78**, 9798–9806 (2004). 15331713 10.1128/JVI.78.18.9798-9806.2004 PMC514997

[64] Léonard, V. H., Kohl, A., Hart, T. J. & Elliott, R. M. Interaction of bunyamwera orthobunyavirus NSs protein with mediator protein MED8: a mechanism for inhibiting the interferon response. *J. Virol.* **80**, 9667–9675 (2006). 16973571 10.1128/JVI.00822-06 PMC1617248

[65] Verbruggen, P. et al. Interferon antagonist NSs of La Crosse virus triggers a DNA damage response-like degradation of transcribing RNA polymerase II. *J. Biol. Chem.* **286**, 3681–3692 (2011). 21118815 10.1074/jbc.M110.154799 PMC3030371

[66] Zou, C. Y. et al. Establishment and characteristics of two syngeneic human osteosarcoma cell lines from primary tumor and skip metastases. *Acta Pharmacol. Sin.* **29**, 325–332 (2008). 18298897 10.1111/j.1745-7254.2008.00756.x

[67] Fan, Z. et al. Establishment and characterization of a highly metastatic human osteosarcoma cell line from osteosarcoma lung metastases. *J. Bone Oncol.* **29**, 100378 (2021). 34221892 10.1016/j.jbo.2021.100378 PMC8243521

[68] Zhu, W. et al. Real-time visualization and quantification of oncolytic M1 virus in vitro and in vivo. *Hum. Gene Ther.* **32**, 158–165 (2021). 33504253 10.1089/hum.2020.273

[69] Lin, C. Y. et al. Transcriptional amplification in tumor cells with elevated c-Myc. *Cell* **151**, 56–67 (2012). 23021215 10.1016/j.cell.2012.08.026 PMC3462372

[70] Camp, R. L., Dolled-Filhart, M. & Rimm, D. L. X-Tile: a new bio-informatics tool for biomarker assessment and outcome-based cut-point optimization. *Clin. Cancer Res.* **10**, 7252–7259 (2004). 15534099 10.1158/1078-0432.CCR-04-0713

