# Supplementary material for: Alphavirus M1 disrupts super-enhancer-driven oncogenic transcription via non-structural protein NSP2 in osteosarcoma
Source: Nat Commun. 2026 Jul 1;17:8208. doi: 10.1038/s41467-026-75013-9 (PMC13463023; doi:10.1038/s41467-026-75013-9)
Supplement: Supplementary file 1 [file 41467_2026_75013_MOESM1_ESM.pdf]

Supplementary information

Alphavirus M1 disrupts super-enhancer-driven oncogenic transcription via non-structural protein NSP2 in osteosarcoma

Jiajun Zhang[1,2,#], Lifeng Yin[1,2,#], Qianqian Han[3,#], Yuanyuan Li[4,#], Fei Wu[1,2], Shanyu Huang[4], Jiayu Zhang[5], Yiwei Fu[1,2], Guanyu Huang[1,2], Yu Xu[1,2], Hanxiao Yin[1,2], Jiankai Liang[4], Wenbo Zhu[4], Yuan Lin[4], Guangmei Yan[4], Junqiang Yin[1,2], Jingnan Shen[1,2,*], Jing Cai[4,*], and Weihai Liu[1,2,*]

[1]Department of Musculoskeletal Oncology, The First Affiliated Hospital of Sun Yat-Sen University, Guangzhou, China.

[2]Guangdong Provincial Key Laboratory of Orthopedics and Traumatology, The First Affiliated Hospital of Sun Yat-Sen University, Guangzhou, China.

[3]Guangdong Provincial Key Laboratory of Malignant Tumor Epigenetics and Gene Regulation, Sun Yat-sen Memorial Hospital, Sun Yat-sen University, Guangzhou, China.

[4]Department of Pharmacology, Zhongshan School of Medicine, Sun Yat-sen University, Guangzhou, China.

[5]Biomedical Innovation Center, The Sixth Affiliated Hospital, Sun Yat-sen University, Guangzhou, China.

[#]These authors contributed equally

*Corresponding authors:

Weihai Liu (E-mail: liuwh25@mail.sysu.edu.cn)

Jing Cai (E-mail: caij53@mail.sysu.edu.cn)

Jingnan Shen (E-mail: shenjn@mail.sysu.edu.cn)

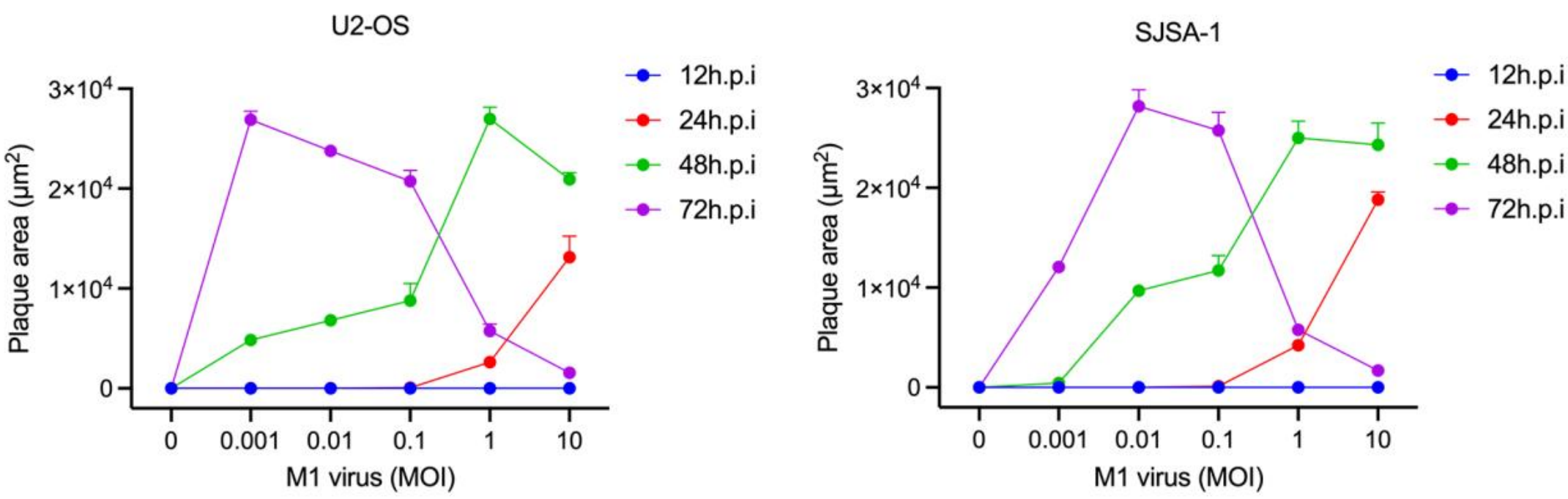

Supplementary Figure 1 | Viral Growth Quantification by Plaque Area, related to Fig. 1
Quantification of plaque area in U2-OS and SJSA-1 osteosarcoma cells infected with oncolytic virus M1 at different multiplicities of infection (MOI). The plaque area serves as a quantitative indicator of viral replication efficiency. Data are shown as the mean ± SD, n = 3 biologically independent samples. Source data are provided as a Source Data file.

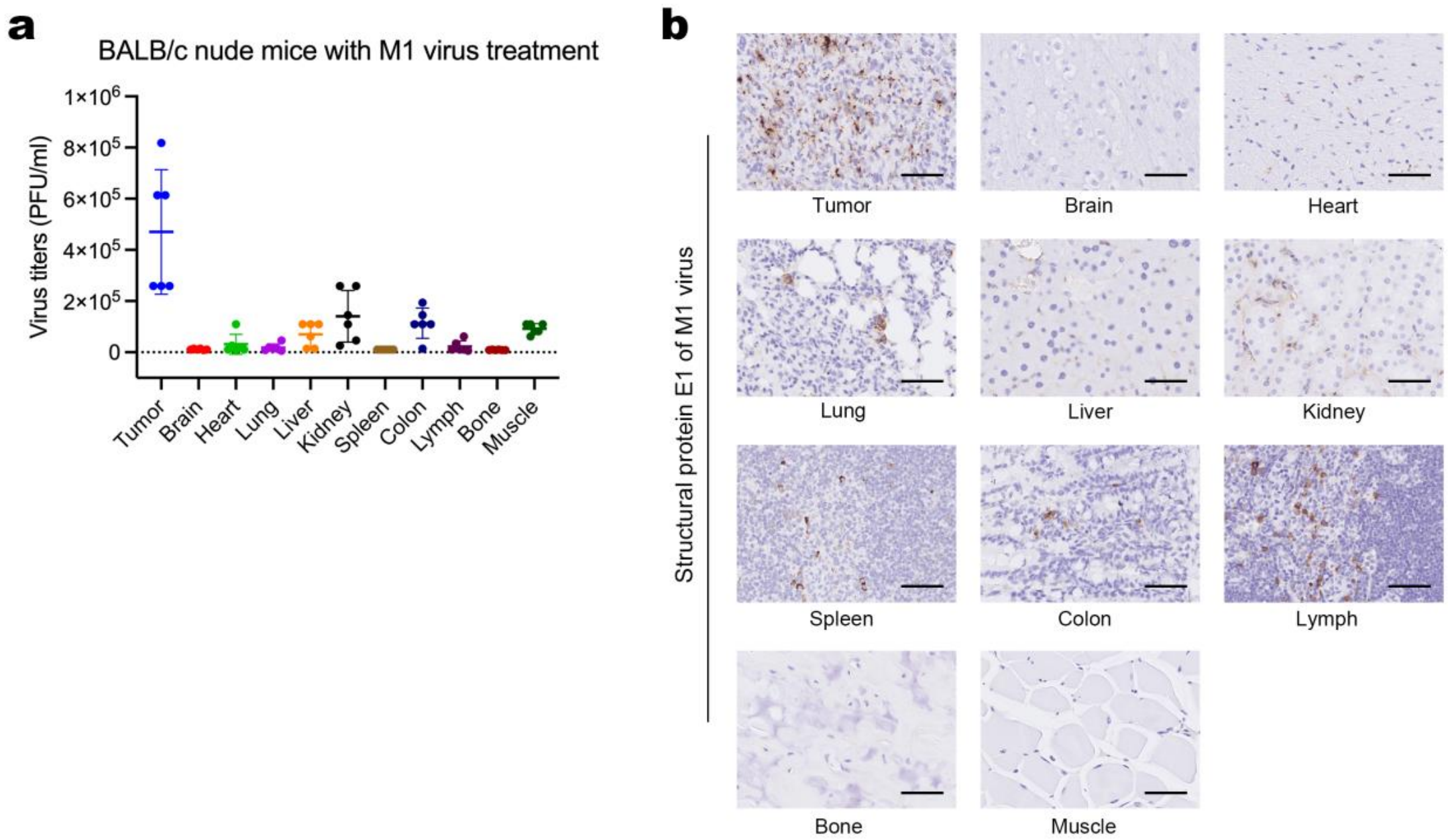

Supplementary Figure 2 | Distribution of M1 virus in mouse tissues, related to Fig. 2
a, Viral titers (plaque-forming units, PFU) in indicated tissues harvested from mice following systemic administration of M1 virus. Data are presented as PFU per mL of tissue homogenate. Data are shown as the mean ± SD, n = 6 biologically independent mice. **b**, Representative immunohistochemistry (IHC) images showing the expression of the M1 virus structural protein E1 in corresponding tissue sections. Scale bar, 50 μm. These complementary assays were performed to accurately assess the biodistribution of M1 virus *in vivo*. Source data are provided as a Source Data file.

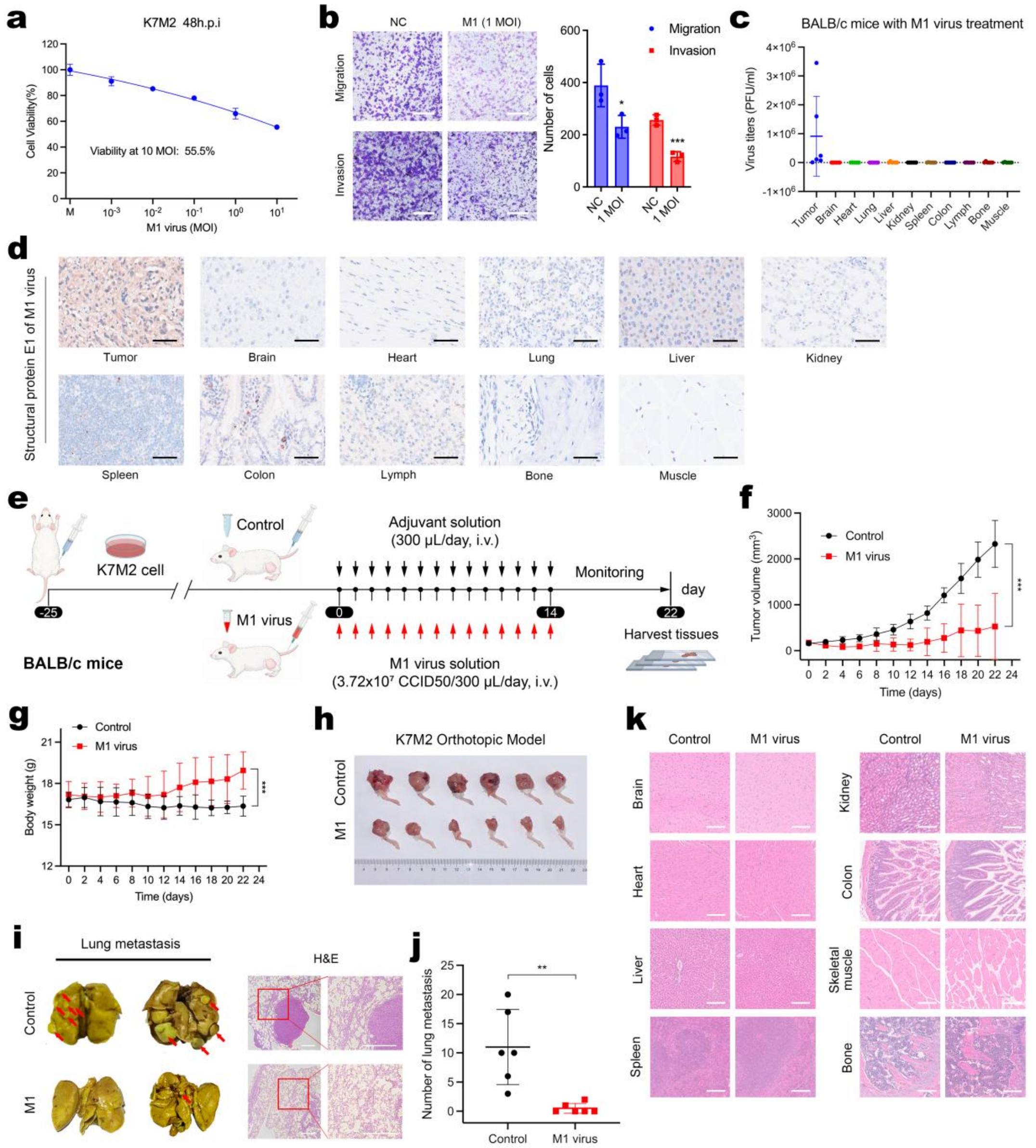

Supplementary Figure 3 | Antitumor activity and biodistribution of M1 virus in an immunocompetent murine osteosarcoma model

a, Viability of murine cell line K7M2 after infection with M1 virus at indicated MOIs for 48 hours. **b**, Representative images of K7M2 cell migration and invasion assay after treatment with M1 virus (1 MOI) or NC (negative control). Scale bars, 150 µm. In (**a**, **b**), data are shown as the mean ± SD, n = 3 biologically independent samples. **c**, Viral titers (plaque-forming units, PFU) in indicated tissues harvested from BALB/c mice on day 4 post-administration of M1 virus. Data are presented as PFU per mL of tissue homogenate. Data are shown as the mean ± SD, n = 6 biologically independent mice. **d**, Representative immunohistochemistry (IHC) images showing the expression of the M1 virus E1 protein in various tissues from treated mice. Scale bar, 50 µm. **e**, Schematic illustration of the experimental timeline for the in vivo therapeutic study. K7M2 cells were implanted into the tibia of BALB/c mice to establish an orthotopic model, followed by intravenous administration of M1 virus or vehicle control. **f**,**g**, Tumor volume (**f**) and body weight (**g**) changes of tumor-bearing mice during the treatment period. Data are presented as mean ± SD (n=6 mice per group). **h**, Images of orthotopic tibial tumors from the control and M1 virus-treated groups at the experimental endpoint. **i**, Representative

lung tissue images showing metastatic nodules (indicated by red arrows) (**i**, left). H&E images were taken at magnification of 40× and 100× (**i**, right). Scale bars, 500 µm. **j**, Quantification of lung metastatic foci from (**i**). Data are shown as the mean ± SD, n = 6 biologically independent mice per group. **k**, Representative H&E images revealed no tissue damage in the brain, heart, liver, spleen, kidneys, colon, skeletal muscle, or bone in M1 treated mice. Scale bars, 150 µm. These data are presented as the means ± SD and were analyzed by two-way ANOVA (**f, g**), and two-tailed unpaired Student's t test (**b, j**). *, P < 0.05; **, P < 0.01; ***, P < 0.001. Source data are provided as a Source Data file.

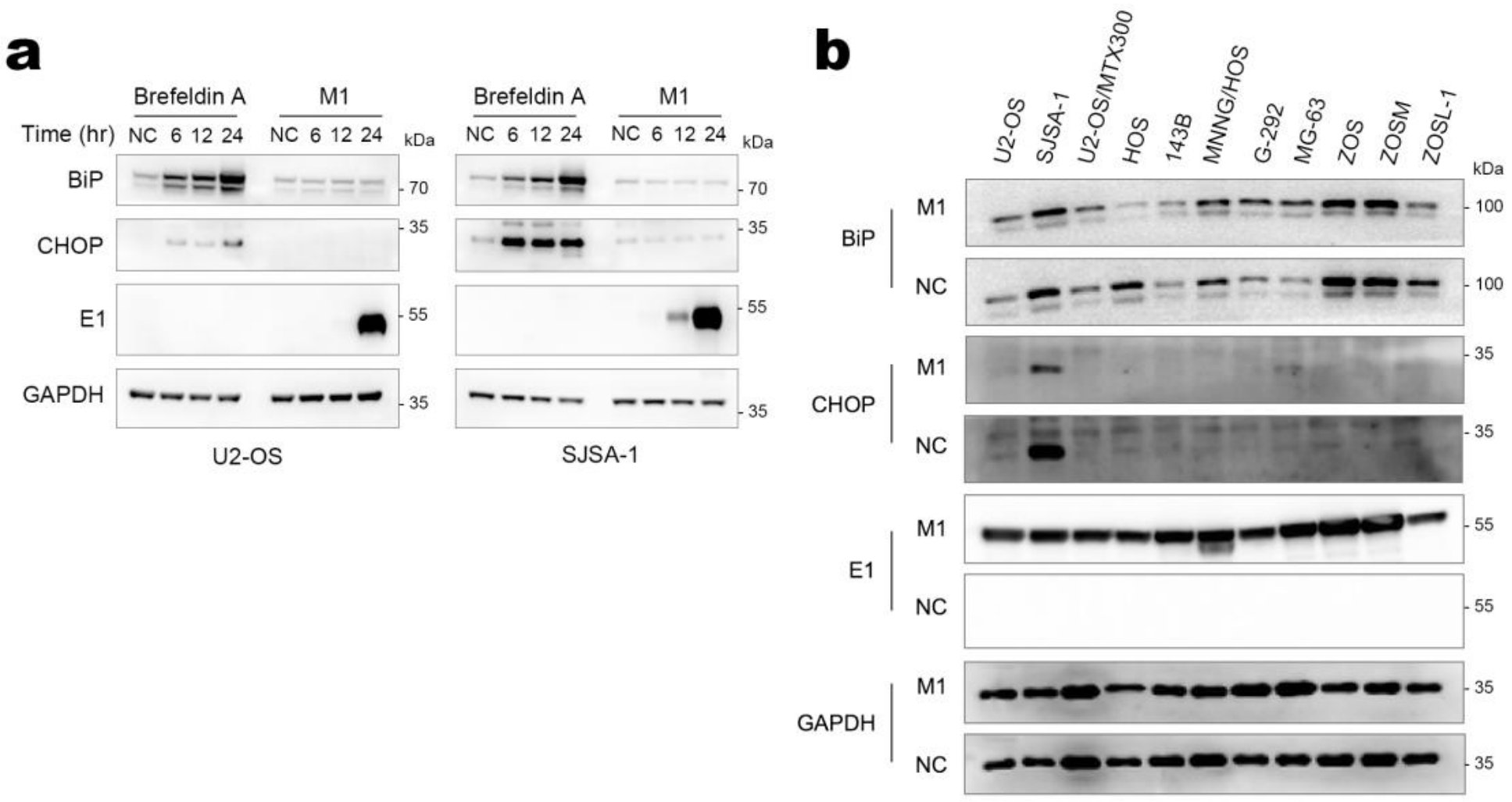

Supplementary Figure 4 | Limited induction of canonical ER stress by M1 in osteosarcoma cells, related to Fig. 3

a, U2-OS and SJSA-1 cells were infected with M1 for the indicated times. Brefeldin A (BFA) served as a positive control. Western blot analysis of ER-stress markers (BiP and CHOP) showed robust induction by BFA but no significant elevation following M1 infection (Viral E1 confirmed infection). **b**, ER-stress markers were further examined in 11 osteosarcoma cell lines. No consistent upregulation of BiP or CHOP was observed after M1 infection (Viral E1 confirmed infection). All experimental data (**a**, **b**) were verified in at least three independent experiments. Source data are provided as a Source Data file.

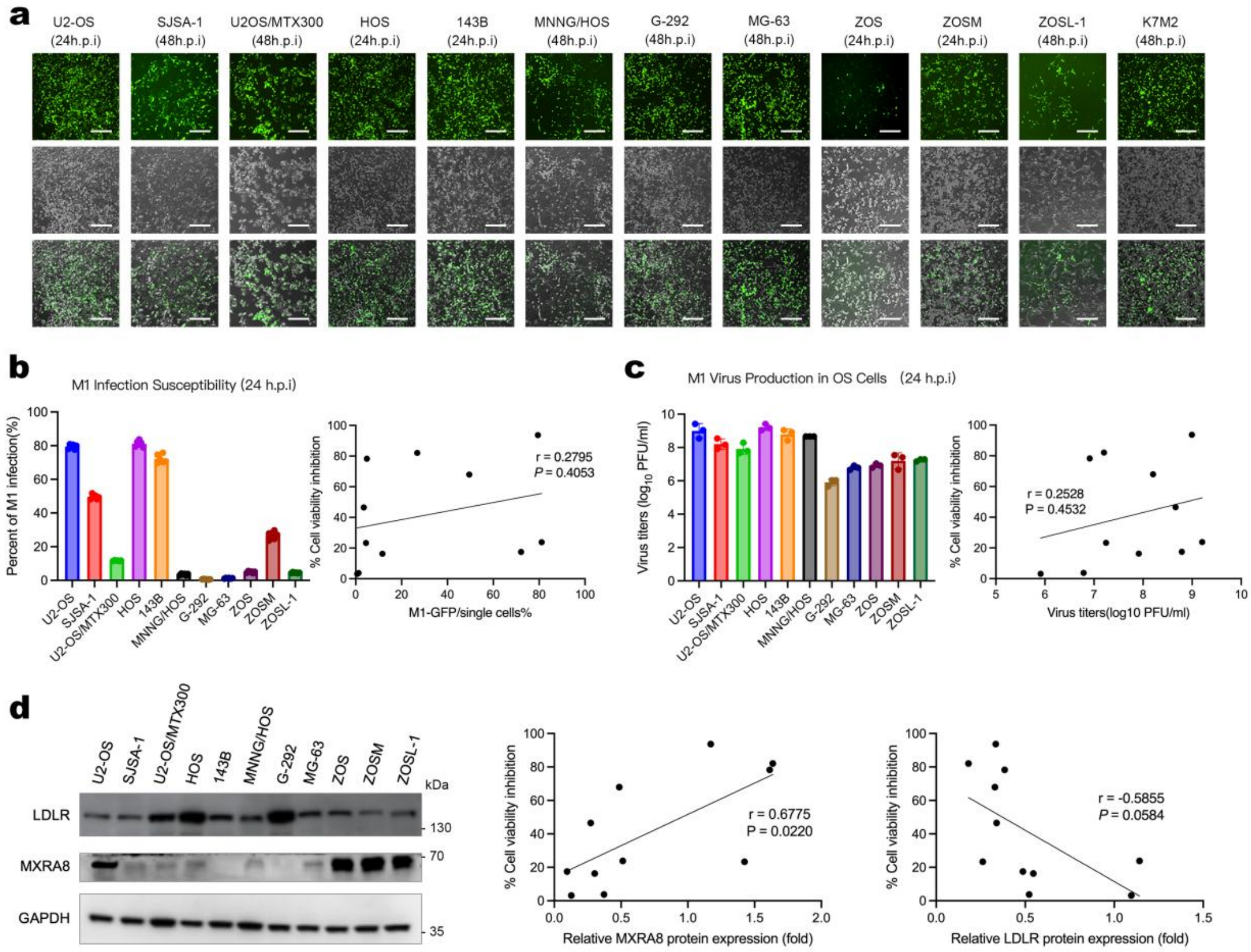

Supplementary Figure 5 | Characterization of M1 virus infection, replication, and receptor correlation in osteosarcoma cells, related to Fig. 3

a, Representative fluorescence images of 11 human osteosarcoma cell lines and the murine osteosarcoma cell line K7M2 infected with M1-GFP virus at the indicated time points (24 or 48 h.p.i). Scale bar, 100 μm. M1 virus demonstrated efficient infection and replication capabilities in all tested osteosarcoma cell lines. **b**, Left: Quantification of M1-GFP-positive cells by flow cytometry in 11 human osteosarcoma cell lines at 24 h.p.i. Data are shown as the mean ± SD, n = 6 biologically independent samples. Right: Correlation analysis between M1-GFP positivity and M1 virus-induced cytotoxicity (cell viability at 24 h.p.i). No significant correlation was observed (r = 0.2795, P = 0.4053). **c**, Left: Viral titers in culture supernatants from 11 human osteosarcoma cell lines at 24 h.p.i, determined by TCID50 assay on BHK-21 cells and expressed as log_{10} PFU/mL. Data are shown as the mean ± SD, n = 3 biologically independent samples. Right: Correlation analysis between viral titers and M1 virus-induced cytotoxicity. No significant correlation was detected (r = 0.2528, P = 0.4532). **d**, Left: Western blot analysis of previously reported alphavirus entry receptors (LDLR and MXRA8) in the indicated osteosarcoma cell lines. Middle: Correlation analysis between MXRA8 expression levels and M1 virus-induced cytotoxicity, showing a significant positive correlation (r = 0.6775, P = 0.0220). Right: Correlation analysis between LDLR expression levels and M1 virus-induced cytotoxicity, showing no significant correlation (r = -0.5855, P = 0.0584). These data indicate that while M1 virus efficiently infects and replicates in osteosarcoma cells, neither infection rate nor viral production directly correlates with oncolytic efficacy, with only MXRA8 expression showing a positive correlation. Source data are provided as a Source Data file.

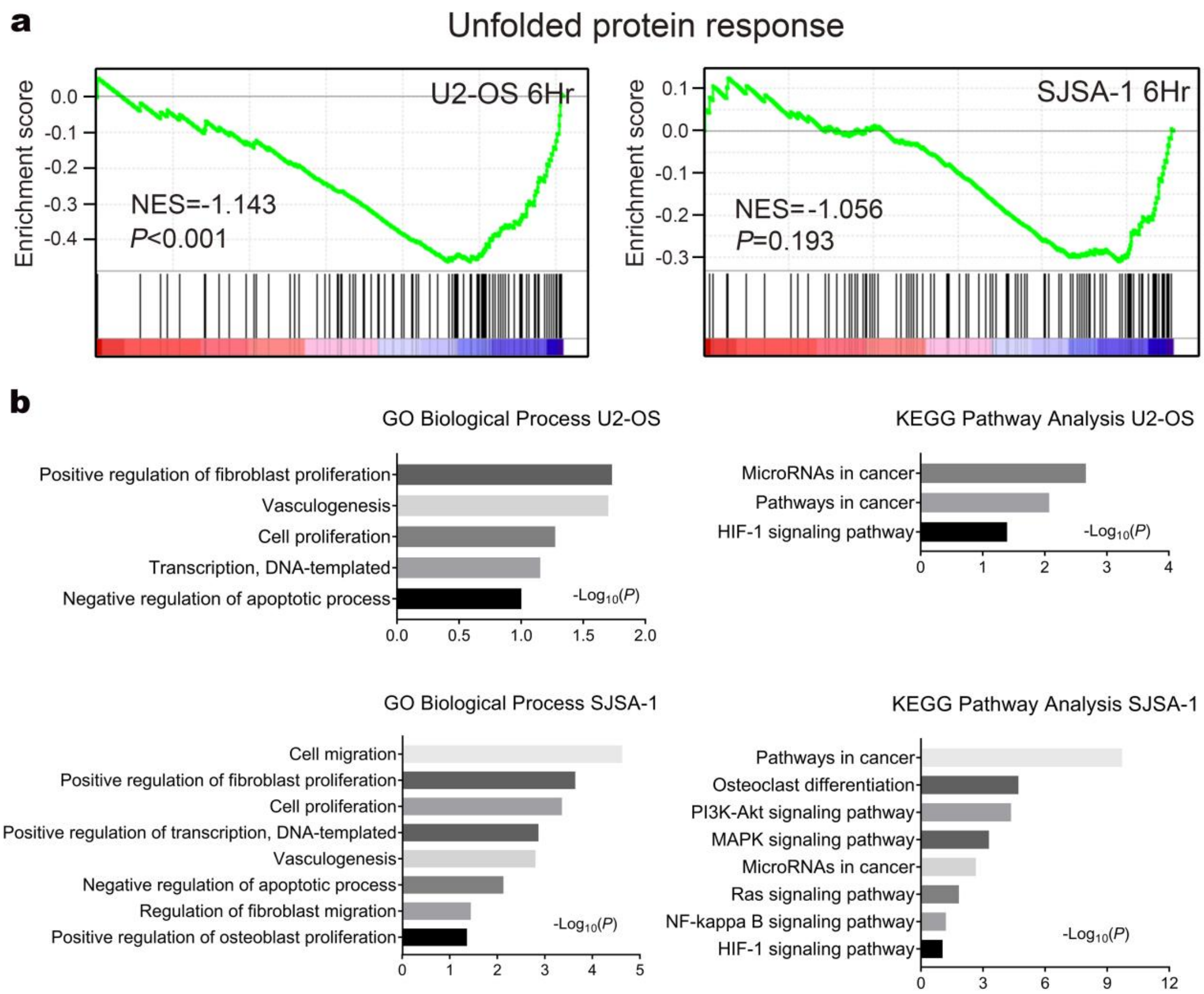

Supplementary Figure 6 | Early transcriptional and epigenetic alterations induced by M1 virus, related to Fig. 4

a, GSEA analysis of unfolded protein response pathway-related gene expression in the early transcriptome of M1 virus-infected cells. P values were calculated by permutation testing. **b**, GO and KEGG clustering analysis of super-enhancer-associated genes with significantly downregulated expression during the early stages of M1 virus infection.

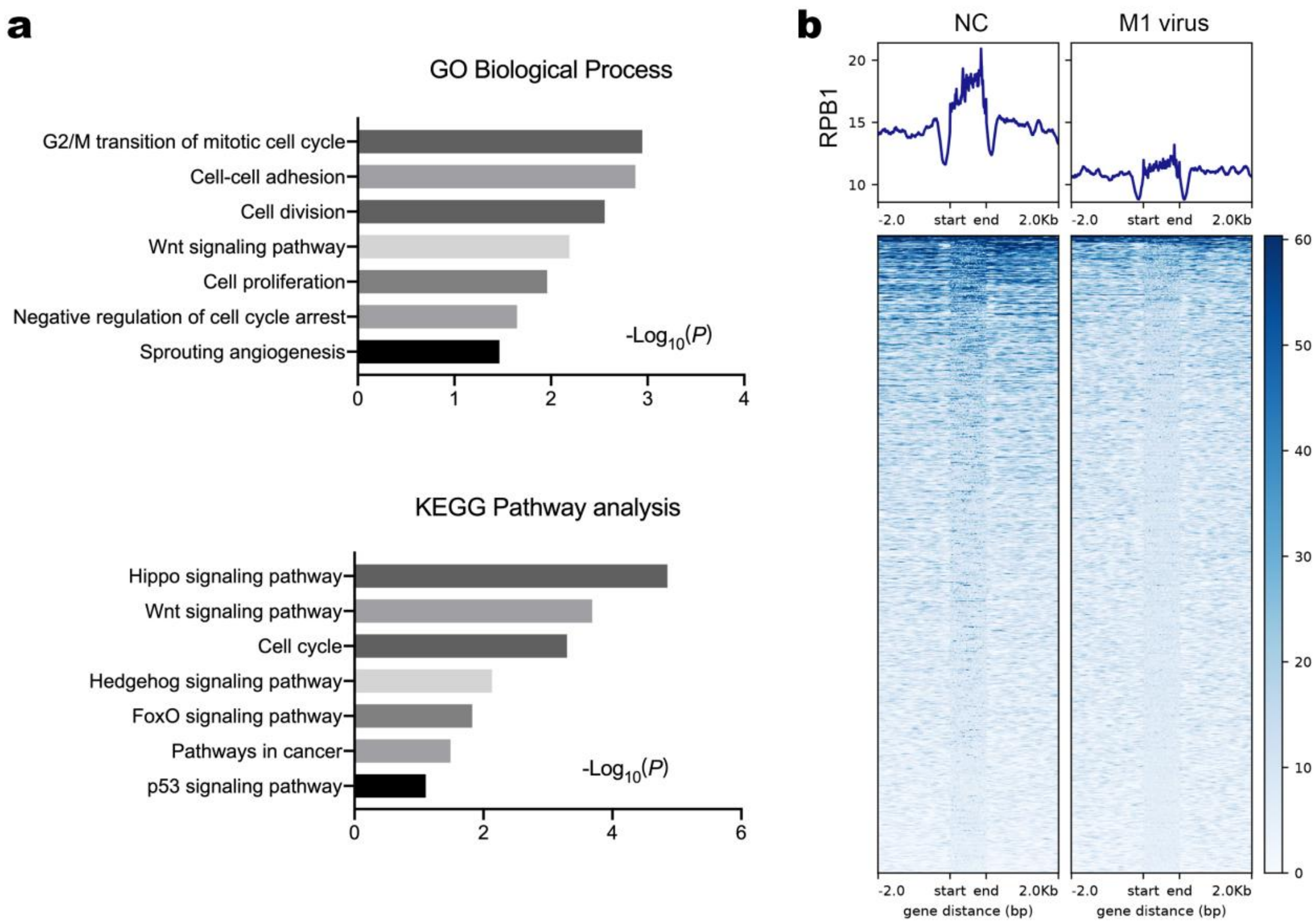

Supplementary Figure 7 | RPB1-SEs link to oncogenic pathways and are disrupted by M1 virus, related to Fig. 5

a, GO and KEGG enrichment analysis of RPB1-SE-related genes. **b**, Heatmap showing changes in RPB1 signal at genomic regions marked by H3K27ac-identified super-enhancers after M1 virus infection.

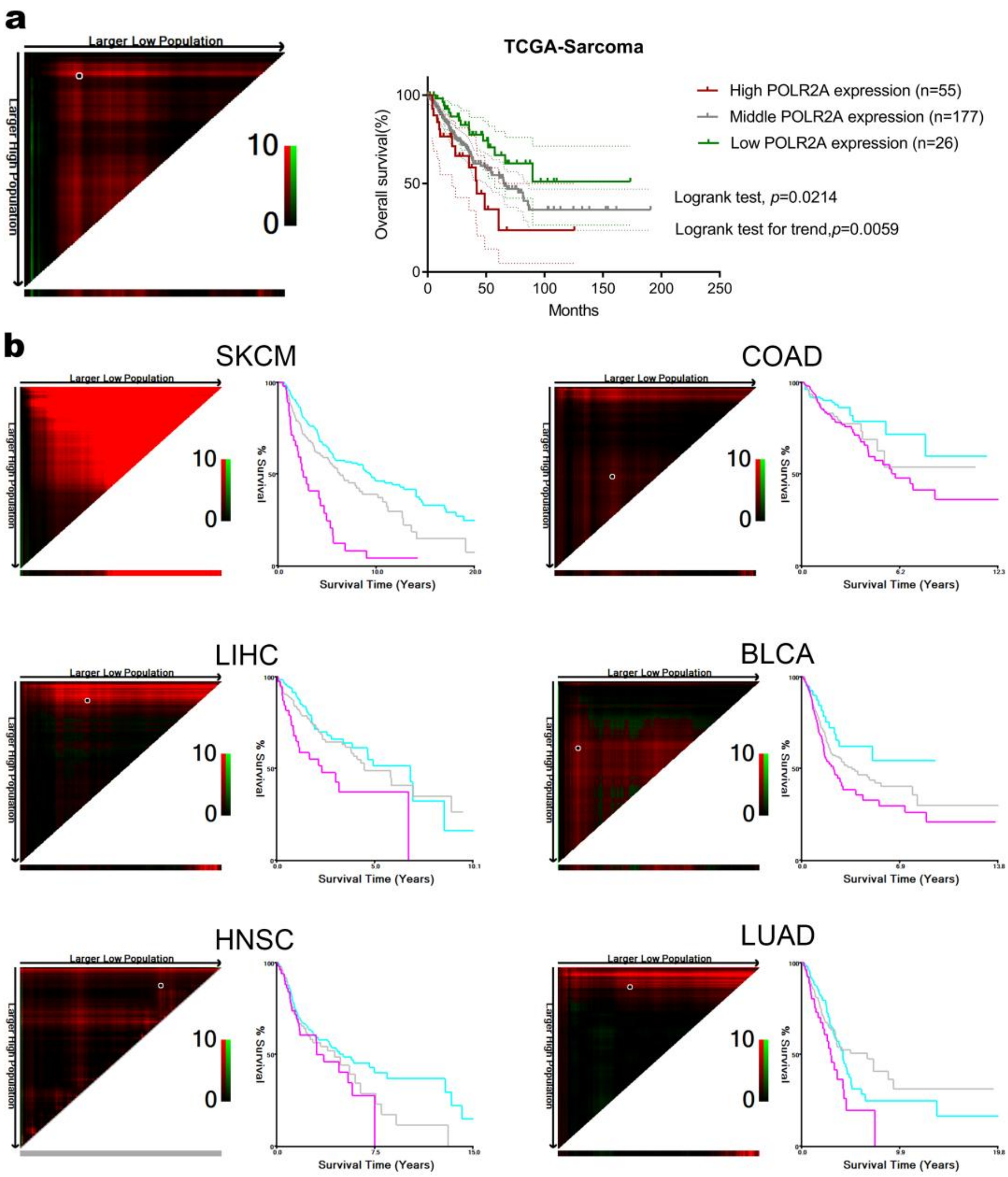

Supplementary Figure 8 | Prognostic significance of POLR2A expression across multiple cancer types, related to Fig. 5

a, X-tile plots and Kaplan-Meier (KM) curve of POLR2A expression for overall survival in 258 sarcoma patients from the TCGA database. The coloration of the X-tile plot, ranging from low (black) to high (red or green), represents the strength of the association at each division, with red indicating an inverse association and green indicating a direct association between marker expression and survival. The optimal cutoff score for POLR2A expression was selected using X-tile plots (black dot) based on its association with survival. The data are presented as the means ± 95% CI and were analyzed by log-rank test. **b**, X-tile plots and Kaplan-Meier curve of POLR2A expression for overall survival in patients with other tumors from the TCGA database, including skin cutaneous melanoma (SKCM), colon adenocarcinoma (COAD), liver hepatocellular carcinoma (LIHC), bladder urothelial carcinoma (BLCA), head and neck squamous cell carcinoma (HNSC), and lung adenocarcinoma (LUAD).

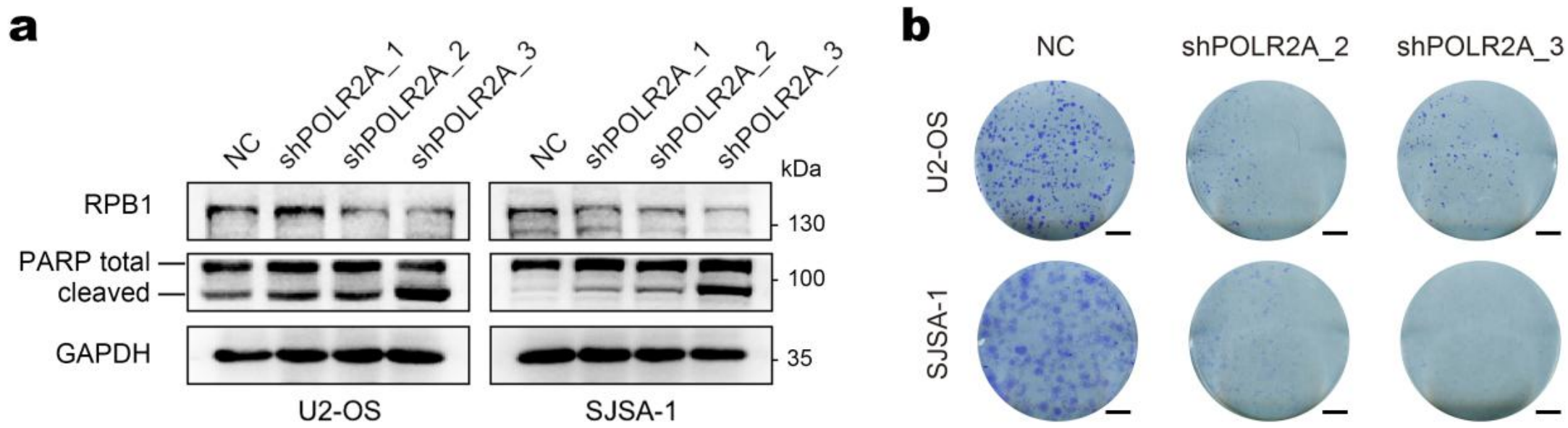

Supplementary Figure 9 | RPB1 depletion impairs osteosarcoma cell proliferation and induces apoptotic signaling, related to Fig. 5

a, Immunoblotting analyses of RPB1, total/cleaved-Parp (apoptosis marker), and internal controls in U2-OS and SJSA-1 cells stably transfected with shRNA targeting POLR2A. Data were verified in at least three independent experiments. **b**, Colony formation assay was used to evaluate the cell growth of U2-OS and SJSA-1 cells transfected with shPOLR2A or control vector. Scale bars, 5 mm. Source data are provided as a Source Data file.

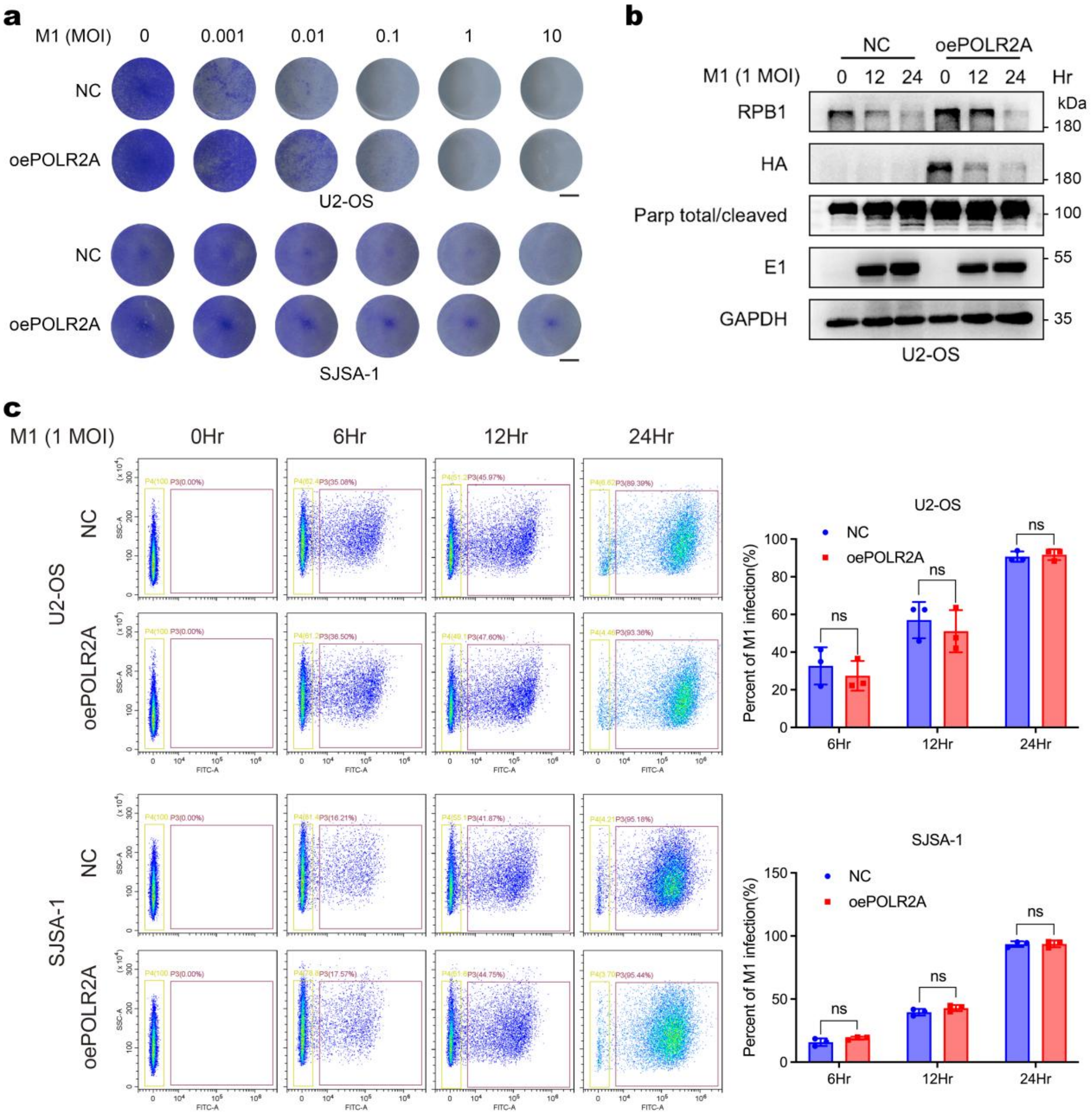

Supplementary Figure 10 | POLR2A overexpression attenuates M1 virus-mediated growth inhibition and apoptosis in OS, related to Fig. 5

Sensitivity of osteosarcoma cells to M1 virus following POLR2A overexpression compared to controls, including colony formation (**a**) and apoptosis (**b**). Scale bars, 5 mm. **c**, Flow cytometry analysis of the number of M1 virus-infected osteosarcoma cells in the POLR2A overexpression group and control group, showing that POLR2A expression did not significantly affect M1 virus infection rates. These data are presented as the means ± SD and were analyzed by two-tailed unpaired Student's t test, n = 3 biologically independent samples (**c**). n.s., not significant. All experimental data (**a-b**) were verified in at least three independent experiments. Source data are provided as a Source Data file.

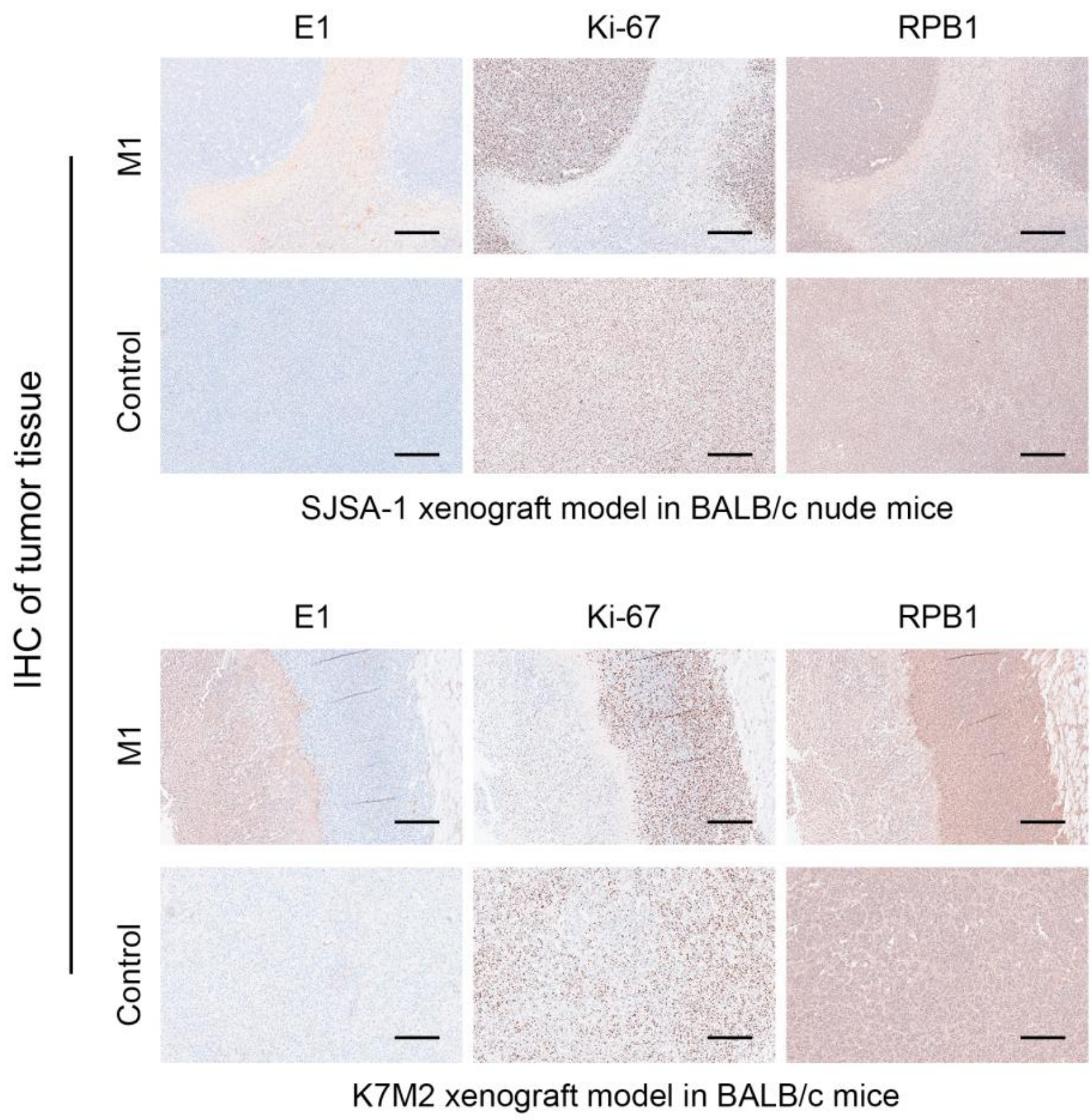

Supplementary Figure 11 | M1 virus downregulates RPB1 expression and suppresses proliferation in osteosarcoma xenografts, related to Fig. 5

Representative immunohistochemistry (IHC) images of tumor tissues from SJSA-1 orthotopic xenografts in BALB/c nude mice (**upper panel**) and K7M2 orthotopic xenografts in immunocompetent BALB/c mice (**lower panel**). Mice were treated with either control vehicle or M1 virus, and tumor tissues were harvested on day 4 post-administration. Sections were stained for the M1 viral protein E1 (infection marker), the proliferation marker Ki-67, and RPB1. Scale bar, 300 μm.

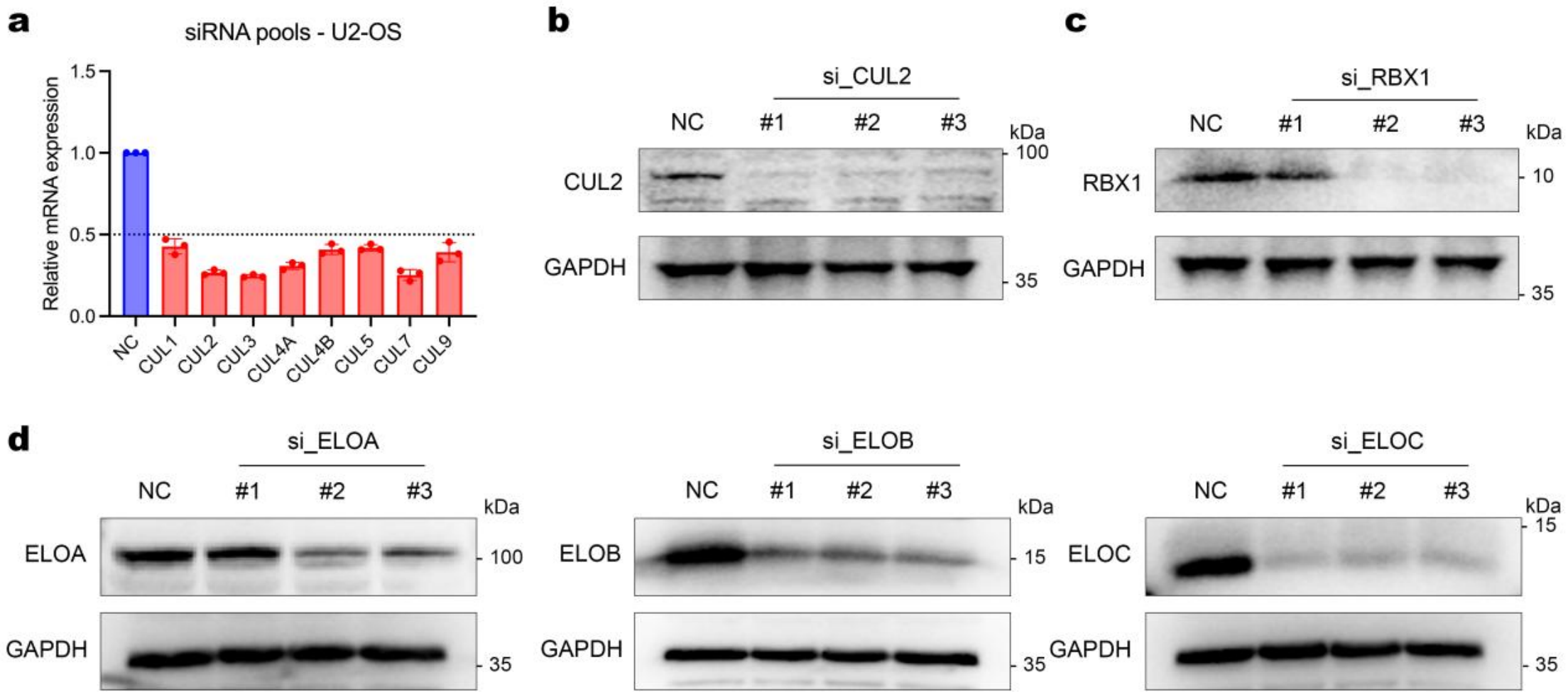

Supplementary Figure 12 | Assessment of knockdown efficiency for E3 ligase components, related to Fig. 6

a, RT-qPCR analysis of mRNA expression of target genes in U2-OS cells transfected with siRNA targeting the CUL family genes or control vector. The data are presented as the means ± SD, n = 3 biologically independent samples. **b-d**, Immunoblotting analysis of protein knockdown efficiency in U2-OS cells transfected with three different siRNA targeting CUL2 (**b**), RBX1 (**c**), and ELOA/B/C (**d**), respectively, or control vector. All experimental data (**b-d**) were verified in at least three independent experiments. Source data are provided as a Source Data file.

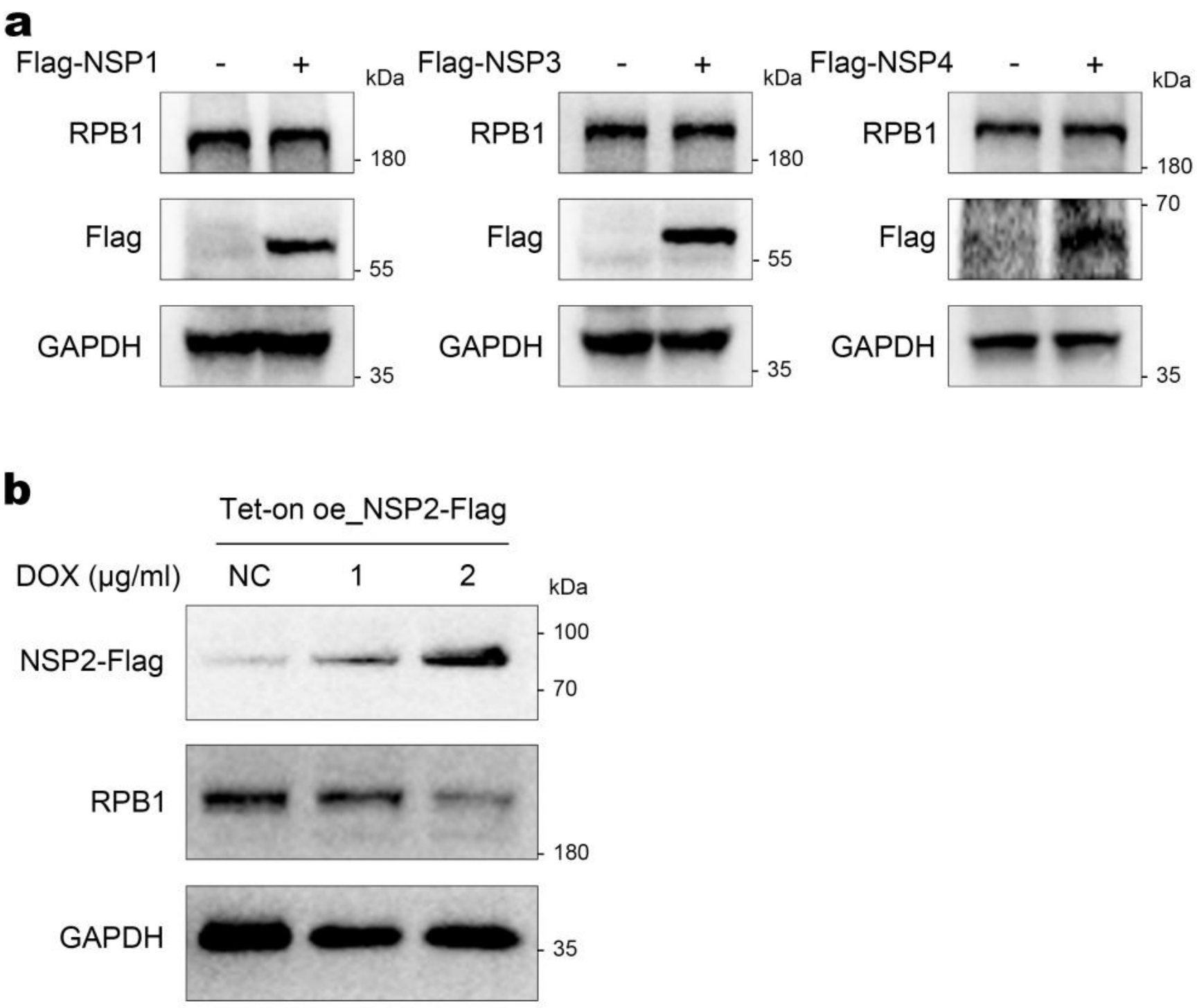

Supplementary Figure 13 | Functional analysis of NSPs in M1 virus-induced RPB1 degradation, related to Fig. 7

a, U2-OS cells were transfected with plasmids expressing flag-tagged NSP1/3/4 and analyzed for alterations in RPB1 protein levels via western blot. **b**, U2-OS cells stably transfected with Tet-on plasmids for conditional expression of NSP2-Flag, induced by DOX, were treated with 1 μg/ml, 2 μg/ml DOX, or vehicle. Immunoblotting was performed to detect changes in NSP2-Flag and RPB1 protein levels. All experimental data (**a,b**) were verified in at least three independent experiments. Source data are provided as a Source Data file.

Supplementary Table 1 | Anticancer efficacy of M1 in various cancer types

Disease	Cell line	Viability (%)	Disease	Cell line	Viability (%)
Osteosarcoma	U2-OS	3.5		LN-229	98.6
	U2OS/MTX300	41.5		SF-767	98.6
	ZOS	14.6		A-172	98.7
	ZOSM	10.4	Liver cancer	Hep3B	3.8
	HOS	42.6		Huh-7	52.2
	143B	65.7		Huh-6	59
	MNNG/HOS	5.8		SK-HEP-1	71.2
	SJSA-1	18.2		Li-7	72.1
	G-292	88.5		PLC	80.5
	MG-63	92		HepG2	88.6
	ZOSL-1	41.9		Bel-7402	99.6
Colorectal cancer	LoVo	6.9	Prostatic cancer	22Rv1	39.1
	HCT-8	35.2		PC-3	72.7
	SW620	43.7		DU 145	91.4
	SW480	53.8	Cervical cancer	C-33 A	14.8
	HCT 116	81.3		HeLa	66
Nasopharyngeal cancer	CNE-2	24.5		Ca Ski	87
	CNE-1	48.2		SiHa	99.6
	HNE-1	76.5		ME-180	103.2
Pancreatic cancer	Capan-1	40.4	Breast cancer	MDA-MB-468	43.7
	PANC-1	49.3		MDA-MB-231	58.9
	SW1990	45.6		T-47D	86.5
	MIA PaCa-2	49.1		SK-BR-3	89.6
	BxPC-3	98.8		MCF7	95.2
	HPAC	30.7	Gastric cancer	NCI-N87	76.4
	PK-59	27.2		HGC-27	79.2
	Hs766T	98.7		AGS	92.3
	CFPAC-1	63.5	Skin cancer	A-375	47.3
Bladder cancer	SCaBER	11.5		M14	92.1
	T24	21.1		MV3	114
	UM-UC-3	39.8	Lung cancer	A549	68.2
	5637	50.2		NCI-H358	87.8
	SW780	88		H1299	96.3
	EJ	95.3		NCI-H460	99.5
	J82	96.8		HCC827	103
Nervous system cancer	U-87 MG	32.4	Hematological cancer	HL-60	85.2
	U-251	34.7		K-562	95.1
	T98G	38.2		CCRF-CEM	110.3
	U-138 MG	40.1		HEL	112
	MGR2	63.2		Reh	113.1
	DBTRG-05MG	83.3			

Supplementary Table 2 | Genomic sequence of M1 virus

>gi|116878336|gb|EF011023.1| Alphavirus M1, complete genome

1 atggcggacg tgtgacatca ccgttcgctc tttctaggat cctttgctac tccacatagt
61 gagagacaaa caacccaaat gaaggtaacc gtggacgttg aggctgatag cccattcctt
121 aaggcccttc agaaggcgtt tcccgccttt gaggttgaat cacagcaggt cacaccgaat
181 gaccatgcta acgctagagc attttcgcat ctggctacta aactgattga gcaagaggtt
241 ccaacaggcg tcaccatcct ggacgtgggt agtgcacccg caaggaggtt gatgtctgac
301 cacacctacc actgcatctg ccccatgaaa agtgcggaag acccagagag gctggcgaat
361 tacgctcgaa agctggcgaa agcatcgggg actgtgctag acaagaatgt gtccggaaag
421 ataacggacc tacaagacgt catggccact ccagacttgg aatccccgac tttttgcctg
481 cacactgacg agacgtgccg cactagggct gaggtcgctg tgtaccagga cgtatacgct
541 gtgcacgcac cgacgtcact gtatcaccag gccatcaaag gtgtcaggac ggcgtattgg
601 attggattcg acaccactcc attcatgttc gaggcactag cgggcgcgta tcctgcgtac
661 tcgaccaact gggcagatga gcaagtgctg caggctcgta acatcggcct gtgcgcgaca
721 ggcctctccg aggggcgtcg cggcaaactc tccatcatga gaaagaagtg cttgcgaccg
781 agcgacagag taatgttttc ggtcgggtcc accttgtaca ccgagagccg aaagctgctg
841 cgcagctggc atttaccttc cgtgtttcac ctgaagggta agaacagttt tacctgcagg
901 tgcgacacgg tggtgtcatg cgaaggttac gtggtaaaga agatcaccat aagcccgggc
961 atatatggaa aaacagtcga ttacgcagtt acccatcacg cagagggttt tctggtgtgt
1021 aagatcactg atacagtcag aggagaaaga gtctctttcc cggtctgtac ctatgtgcct
1081 gcaaccatat gcgatcagat gacgggtata cttgccaccg acgtgacacc agaggatgcc
1141 cagaagctcc tggttggatt gaaccaacgc atagtggtga atggtaggac gcaaagaaac
1201 acaaacacaa tgaaaaacta tctactgcca gtggtagcgc aagcattcag taaatgggca
1261 cgagaggcac gcgcagacat ggaggatgaa aaacccctag gcaccagaga acgcacgttg
1321 acgtgttgtt gcctgtgggc gtttaaaagc cacaaaaccc acaccatgta taagcggcct
1381 gaaactcaaa ctatcgtcaa agtgccttcc acttttgact cctttgtgat accgagcctg
1441 aggtcatcca gtctttccat ggatatcaga cggaggatca aactgctact cagcgcaaga
1501 atggcccaag gcctaccata ctcaggagac cgcactgaag ctcgcgcggc agaagaagaa
1561 gagaaggagg cgcaggaggc agaacttacg agggcagcgc tgccaccgct agtgagcggc
1621 tcttgtgctg acgatatcgc ccaggtggat gtagaggaat taaccttcag agccggagcc
1681 ggggttgtgg aaacacccag gaatgccctg aaggttacac cgcaagcaca cgaccacctc
1741 ataggctcct acttgatcct ttccccccaa acggtgttga aaagcgagaa gctggcaccc
1801 atccaccctc ttgctgagca agtcacggtc atgacccact ctggaagatc cggcagatac
1861 ccagtcgaca agtacgacgg acgggtattg atcccaacag gagcggccat cccagtgagt
1921 gagttccagg cactcagcga gagcgcaacc atggtgtaca atgagaggga atttataaat
1981 cgcaagctac accacatagc gctatacggg ccagccttga ataccgacga ggaaagctac
2041 gaaaaagtga gagctgagag ggcagagaca gagtatgtgt ttgacgtgga caagaaggca
2101 tgtatcaaga aggaggaggc atcaggcctt gtgttaacag gggacctaat caatccacct
2161 ttccacgaat tcgcatacga aggactcaag atccgcccag cagccccgta ccacacgacg
2221 atcattggtg tgtttggcgt tccaggttcg ggcaagtcgg ctatcattaa gaacatggtg
2281 acgactcgcg atctggtggc cagtggaaag aaggagaact gccaagagat catgaatgat
2341 gtaaagaggc aacgcgggtt ggacgtgacc gctaggaccg tcgactcaat cttattgaat
2401 gggtgcaaga aaggcgtaga aaacctttac gtcgatgagg cgttcgcgtg tcactcgggc
2461 actttgctag cgctcatcgc gctggtgaga ccgtcaggta aggtagtact gtgcggcgac

2521 cccaagcagt gtggtttctt caatttgatg caactgaagg tgcactataa ccacaacatt
2581 tgtacaaggg tgctccataa gagcatctcc agaagatgca ctctacctgt tacggcgatc
2641 gtgtccacct tgcactacca agggaagatg agaacgacga accgatgcaa cacccccatt
2701 cagattgaca ccaccggttc ttccaaacca gcctcaggag atatcgtgtt aacgtgcttc
2761 cgcggctggg tgaagcaact gcaaatcgac tatcgtggac acgaggtgat gtccgcagct
2821 gcttcccagg gtctgacaag gaaaggcgtg tacgccgtga gacagaaagt gaacgaaaac
2881 ccactgtact caccactgtc ggagcacgtc aatgtgctgt tgacccgaac tgaaaaccga
2941 ctggtgtgga agacactgtc gggtgacccg tggataaagg tgttaaccaa tgttccacgt
3001 ggggatttca gtgcaacttt ggaggaatgg caagaagaac atgacggtat catgagagtg
3061 ttgaacgagc gaccggcgga ggttgatcca ttccaaaaca aggccaaagt gtgctgggca
3121 aaatgtctgg tgcaagttct tgagacggcc ggaatacgta tgacggcaga tgaatggaac
3181 accatcttgg ctttcagaga ggacagagcg tactcaccag aagtcgctct caacgagatt
3241 tgcactcgtt actacggcgt tgacctagac agcggcctat tctcagcgca gtcagtttcc
3301 ctcttttatg agaacaacca ctgggacaac aggcccggag gacgcatgta cgggttcaac
3361 catgaagtag ccaggaaata tgcagccagg tttccatttc tacgtggcaa catgaactcg
3421 gggctacaac taaacgtccc tgagaggaag ctccaacctt ttagcgctga atgcaatata
3481 gtcccatcca atcgtcggtt accgcatgct ctagtcacaa gctatcagca gtgccgtggg
3541 gagagggtag agtggttgct gaaaaagatt ccaggtcacc aaatgttact tgtaagtgag
3601 tacaacctgg tgatacctca caaaagagtc ttctggattg cacctccgcg ggtgtcaggc
3661 gcggaccgca cgtacgacat ggacctaggg ttacctatgg atgcaggccg ttacgatctg
3721 gtattcgtca acatccatac tgagtaccgg caacaccact accaacaatg cgtcgaccat
3781 tcaatgcgtc tgcagatgct gggaggggat tcactacacc tgcttagacc aggaggctcg
3841 ctgctgatga gagcatatgg ttacgcagac agagtcagcg agatggtggt gacagccctg
3901 gctaggaaat tctcggcgtt ccgtgtcctg agaccggcgt gtgtgacgag caacacagaa
3961 gtgttcctgc tgttttctaa ctttgataac ggcagaagag cggtaacctt gcaccaagct
4021 aaccagaaac ttagctcaat gtatgcctgc aacggactgc acactgctgg ctgtgcaccg
4081 tcatacaggg tccgccgcgc agatatatca ggacacggtg aggaagcagt cgtaaatgct
4141 gccaatgcca agggtaccgt gagcgatgga gtgtgcaggg cggtcgctaa gaagtggcca
4201 tcatctttca aggggctgc aactccagtc ggcacagcca agatgatccg cgcagatggc
4261 atgaccgtaa tccacgcagt gggaccgaac ttctccaccg taacagaagc cgaaggggac
4321 agagagctag cggccgcgta ccgagctgtg gctagcataa ttagtaccaa caacataaag
4381 agcgtcgcag taccgctgct atccacaggc accttttccg gcggtaagga cagagtgatg
4441 cagtccttga accacttatt cacggcattg gatgccaccg atgcagacgt ggttatctac
4501 tgcagagata aaaactggga aaagaagatt caggaagcca tcgacaggcg gacggcaata
4561 gagctcgtat ccgaagacgt gaccttggaa accgatctgg ttagggtaca cccggacagt
4621 tgcttagtcg gcagaaatgg ttacagtgca actgacggta aactgtactc ttaccttgag
4681 ggcacgaggt tccaccagac ggcggtcgac atggctgaaa tatcaacttt atggccaaga
4741 ctccaagatg ctaacgagca gatctgcctg tacgccctag gggagacgat ggacagcata
4801 cgcactaaat gcccagtaga ggacgccgat tcgtctacgc cgccgaaaac ggtaccgtgt
4861 ctatgtcggt acgcgatgac cgcggagcgg gttgccagac ttaggatgaa taacaccaaa
4921 aacatcatcg tgtgctcctc ctttccattg ccgaagtaca ggatagaagg cgtgcagaag
4981 gtgaagtgtg accgagtgct aatttttgac cagaccgtcc cgtcactagt aagtcccaga
5041 aagtacatac agcagccgcc ggaacagctg gataatgtga gcctgacttc tacgacgtcg
5101 acgggatccg catggtcatt tccatcggaa acgacctacg aaaccatgga agtcgtagcc

5161 gaggtacaca ccgaacctcc gatccctccg cctcgccgac gtagagcagc cgtcgcccaa
5221 cttagacagg atctggaagt caccgaggag atcgagccgt acgtgataca gcaagcagag
5281 atcatggtca tggagagggt cgcgacgaca gacatacgcg ctatcccagt cccggcacgg
5341 cgggccatta caatgccagt cccagccccc agtgttcgta aggtcgctac cgaacctcca
5401 ttagaaccgg aagctcctat cccggcacca agaaagagaa gaaccactgg caccacacct
5461 ccgcataacc ccgaggattt cgttcccagg gtaccggttg agttaccgtg ggagccggaa
5521 gacctagaca tccaattcgg tgacttggag ccacgccgcc ggaacaccag ggaccgagat
5581 gtcagcacag gaatacagtt cggtgacatc gactttaacc agtccagact aggcagggct
5641 ggcgcgtata tcttttcgtc tgacactggc ccgggtcacc tacagcagaa gtccgtaagg
5701 caacatgaat tgccatgcga gactctgtat gcccatgaag acgaacgcat atacccgccg
5761 gcatttgacg gagagaaaga gaaggtactc caggcaaaga tgcagatggc cccgacagaa
5821 gcgaataaga gcaggtacca gtcgaggaaa gtagagaaca tgaaggcatt aattgtagaa
5881 agactacgcg aaggagcaaa gatgtacctc catgagcaaa ccgacaaagt acccacgtac
5941 accagcaagt accctagacc tgtgtactca ccatcggtgg atgacagcct gagcgatccg
6001 gaagtggctg tggccgcctg taactctttc ttagaggaga attatccgac cgtggcgaac
6061 taccagataa ccgatgagta tgacgcttat ctggacttgg tcgacggctc tgaaagctgc
6121 ctcgacagag ctacgttctg cccggccaaa ctaagatgtt accctaagca ccacgcatac
6181 caccaaccac aaatcaggag cgcagtacct tcccctttcc aaaacacgtt acaaaacgtg
6241 ctagccgcgg ccactaaaag aaattgtaat gtcacccaaa tgagagaatt accaaccatg
6301 gactctgcgg tgtttaacgt agaaagcttc aaaaaatacg cctgtaccgg cgaatattgg
6361 caagaattta aagacaatcc tatacggatc accaccgaaa acataacgac gtacgtggct
6421 aaactcaagg gtccaaaggc tgctgccctt tttgccaaga cgcataacct ggtgccgctc
6481 caggaggtgc caatggaccg cttcgtgatg gatatgaaga gagatgtgaa agttacacca
6541 ggcaccaagc ataccgaaga aaggcctaaa gtgcaagtga ttcaagcggc ggaaccattg
6601 gccacggcat atttatgcgg aatccacaga gagttagtca ggcggctaaa agccgttctg
6661 accccgaaca ttcacactct gtttgacatg tcggcggagg actttgatgc catcatagcg
6721 gcacattttc aaccgggaga tgctgtactg gagacagata tcgcatcctt cgataagagc
6781 caagacgact ccttagcgct aacggcgcta atgcttctgg aagacctcgg ggtcgaccaa
6841 gaactgctgg accttatcga agctgcgttt ggtgagatca cgggtgtgca tctacctacc
6901 ggtacaagat ttaaattcgg tgctatgatg aagtcaggaa tgtttcttac actcttcatc
6961 aacacgctgc tgaacattgt catagcgtgc cgcgtcttac gcgacaaatt atcgtcctcg
7021 gcgtgcgccg ccttcatagg tgatgacaac atagtgcacg gcgtgaggtc agacccgcta
7081 atggcagaaa ggtgtgcgag ttgggtcaac atggaagtaa agatcatcga tgccacaatg
7141 tgtgagaaac caccgtactt ttgtggagga ttcatcctgt acgacagtgt cgccggtaca
7201 gcgtgtaggg ttgcagaccc gttaaagagg ctgttcaaac tcgggaaacc gctcccagcg
7261 gacgacaacc aggatgaaga cagaagaagg gcactaaaag acgaaacagt taagtggtcc
7321 cgcataggat tgagagaaga attagacgtg gcactgagct caagatacca agtcagtggc
7381 gtcgggaaca tcactagagc gatgtccacg ctgtctaaga atttgaagtc ttttaggaaa
7441 ataagaggtc ccattataca tctgtacggc ggtcctaaat agatgcagga ttacactaca
7501 tctaaagacc acgtattaca gacaccatga attacatccc aactcaaacc ttttacggac
7561 gccgttggcg accacgcccg gcgttccgtc catggcgggt gccgatgcag ccggccccac
7621 ccatgattcc agagctgcaa actccgatcg tccaggccca acagatgcag cagctaatca
7681 gtgcagtttc tgccctgacg actaagcaaa atggcaaagc accgaagaag ccgaagaaaa
7741 agccgcaaaa agcgaaggct aagaaaaacg aacagcaaaa gaaaaacgag aacaagaaac

7801 caccacctaa gcagaagaat ccggctaaga agaagaaacc aggaaaaagg gaacgcatgt
7861 gcatgaagat agaaaatgat tgcatcttcg aggtcaagct tgacggtaag gtcacgggat
7921 acgcctgcct agtcggggat aaagtgatga agccggcaca cgtcaaaggt gtgatcgaca
7981 accccgacct agcgaagctt acctacaaga aatcgagcaa gtatgaccta gagtgcgccc
8041 agataccggt gcacatgaag tcagatgctt caaaatacac ccatgaaaaa ccagaagggc
8101 actacaattg gcatcacggt gcagtgcagt acagcggtgg caggttcaca atcccgacag
8161 gcgcaggtaa accaggagac agcggccggc cgatcttcga caacaaagga cgtgtggtgg
8221 ccattgtcct gggaggggcc aacgaaggag ccaggactgc cctatctgtc gtgacctgga
8281 ccaaagacat ggtcacacgg tacaccccag aaggaacaga agaatggtcc gccgccttga
8341 tgatgtgcgt cttagccaac gttacattcc cacgctcaga gcccgcatgt gcaccctgtt
8401 gctatgaaaa acaaccagaa cagacactga ggatgttgga ggacaacgtg gaccgcccgg
8461 gctactacga cctgctcgag gccacgatga cgtgtaacaa cagtgcacgc caccgtcgca
8521 gtgtgacgga acacttcaat gtctacaagg ccacgaaacc gtacctagcg tattgcgcgg
8581 actgcggaga cgggcagttc tgttacagcc cggtggctat agaaaaaatt agggatgagg
8641 cttccgatgg catgataaag atccaggtcg cagcgcaaat tggcatcaac aaaggaggaa
8701 cacacgaaca caacaaaatc aggtacatcg ccgggcatga catgaaagag gcaaaccggg
8761 attctttaca agtgcatact tccggtgtgt gcgctattcg aggcacgatg ggccacttca
8821 tcgtggccta ctgccctcca gggggcgaac taaaggtcca gttccaagat gcagaatcgc
8881 acacacaggc ctgcaaagtg cagtacaaac acgctccggc cccagtaggc agagaaaaat
8941 tcaccgtcag gccccacttc ggtatcgaag tgccatgcac aacgtaccag ctgactaccg
9001 caccgacgga ggaagagatc gacatgcata ccccaccgga tatcccagac ataacgttgc
9061 tgtcgcagca gtcaggtaac gtaaagatca cagcaggagg aaaaaccatc agatacaact
9121 gcacgtgtgg tagtggcaac gtgggcacca ccagtagcga caagactatc aattcgtgca
9181 aaatagcaca gtgccacgct gcggtgacta accacgataa gtggcagtac acctcctcgt
9241 ttgtccctag agccgaccag ttgtctcgca aaggtaaagt gcacgtaccc ttccctctga
9301 ccaactccac atgcagggtg ccccttgcac gtgcaccagg tgtcacatac ggaaagagag
9361 aactgacagt gaaactgcat ccagatcatc ccacgctgtt gacgtaccgg agtctaggag
9421 cagatccgcg tccgtatgag gagtggatag accgatacgt cgaacggacc ataccggtga
9481 ccgaagatgg gatagagtac agatggggaa acaacccacc cgtgcgcttg tgggcccagc
9541 tgacaactga aggcaaaccc catgggtggc cgcacgagat catactctat tactatgggc
9601 tatacccagc agccaccatc gtcgctgtct cagccgcgtg tctcgcagtc gtactatcgc
9661 tgctggcgtc atgttacatg ttcgccactg cacgccgcaa gtgcctgacc ccatacgccc
9721 tgacccccgg agccgtcgtc ccggtaacac taggagtact atgctgcgca ccacgagcgc
9781 atgccgcgtc gtttgcggaa tctatggcgt atctatggga tgagaatcaa accctgtttt
9841 ggctggagct tgcaacgccg ctcgctgcca taatcatact tgtatgctgc ctgaagaacc
9901 tgctttgctg ctgcaaaccg ctatcttttt tagtgttggt gagcctggga actcccgtcg
9961 taaaatctta cgaacacacc gcaacgatcc cgaatgtggt gggattcccg tataaggctc
10021 acattgagag gaacggcttc tccccgatga ccctacagct tgaggtactt ggaaccagct
10081 tggaacccac actaaactta gagtacataa cctgtgaata caagacagtc gtgccgtcac
10141 cttatatcaa gtgctgcggg acatcagaat gcagatccat ggagcgcccc gactatcaat
10201 gccaggtcta cacaggagtg tacccattta tgtggggcgg cgcatactgc ttctgcgaca
10261 ctgagaacac ccagctgagt gaagcatacg ttgacagatc ggacgtatgc aagcacgacc
10321 atgccgcggc ctacaaggcg catactgcgg caatgaaagc caccatccga ataagctacg
10381 ggaacctcaa tcagacaaca acggcgttcg tcaacgggga gcacacagtg accgtcggag

10441 gcagcaggtt tacttttggt ccaatctcca ctgcctggac gcctttcgac aacaagattg
10501 tcgtctataa gaacgacgtc tacaacctgg acttcccacc ctacgggtca ggacaaccag
10561 gaaggtttgg agacatccag agcaggacgg tagagagcaa ggacctgtat gccaacaccg
10621 ccctcaagtt gtcaagacct tcgtccggta ctgttcacgt gccttacaca cagacccctt
10681 ctggctttaa gtactggata aaagagagag gcacgtcgct gaatgacaag gctccctttg
10741 gatgcgtaat caagaccaac ccagttagag cagaaaattg cgccgttggc aacatcccag
10801 tctccatgga catcccggac tccgcgttta cgcgcgtgat tgatgcacct gccgtcacaa
10861 acctggagtg ccaagtggcg gtctgcacgc actcaccgga cttcggcggg attgcgactc
10921 tgactttcaa aaccgacaaa cccggaaaat gtgctgtcca ttctcattcg aacgtagcca
10981 ccatacagga ggcagctgtg gacatcaaaa cagatggcaa gataaccctg catttctcta
11041 cagcatcagc atccccggca ttcaaggtat ctgtgtgcag tgccaaaacg acatgcatgg
11101 cagcgtgtga gccgccgaag gattacatcg tcccttatgg ggcgagccat aacaaccaag
11161 tttttcctga catgtctggc acggcaatga catgggtgca gcgggtagct ggcggattcg
11221 gcgggctaac actcgccgct gtggcagtac ttatactggt gacgtgtgtg actatgcgcc
11281 gctaaccggg aggcttgaca taatgtatat atataagcat catagtttta ataaagcaca
11341 taaataatca agtagatcaa agggctacct aacccctgaa tagtaacaaa acgcaaaata
11401 caaaaacatt agttcaaagg gccagtaacc cctgaatagt aacaaaacat aaaaaccaaa
11461 aaaaaacagt agttcaaagg gctatacaac ccctgaatag taacaaaata cagaaaaacc
11521 ataaaaatta taaaattaac taatctgatc atctaaattt gactaattgg aaatagccga
11581 actctacgga gatgtaggcg tccgaactcc acggagacgt aggacaaaat tctgccgaac
11641 cccagaccat cggggacgta ggcgtctaat ttgttttttt aatattttac

Materials Detailed Information

**Antibodies**		
H3K27ac antibody	Abcam	Cat# ab4729; RRID: AB_2118291
RPB1 antibody	Abcam	Cat# ab26721; RRID: AB_777726
GAPDH antibody	Cell Signaling Technology	Cat# 2118S; RRID: AB_561053
Ubiquitin antibody	Cell Signaling Technology	Cat# 43124; RRID: AB_2799235
CUL4A antibody	Cell Signaling Technology	Cat# 2699; RRID: AB_2086563
CUL2 antibody	Proteintech	Cat# 10981-2-AP; RRID: AB_2086422
RBX1 antibody	Proteintech	Cat# 14895-1-AP; RRID: AB_2179719
ELOA antibody	Proteintech	Cat# 10502-1-AP; RRID: AB_2201278
ELOB antibody	Proteintech	Cat# 10779-1-AP; RRID: AB_2240375
ELOC antibody	Proteintech	Cat# 12450-1-AP; RRID: AB_2201139
FLAG antibody	Cell Signaling Technology	Cat# 14793; RRID: AB_2572291
Total/cleaved-PARP antibody	Abcam	Cat# ab191217; RRID: AB_2861274
LDL Receptor antibody	Abcam	Cat# ab52818; RRID: AB_881213
MXRA8 Antibody	Thermo Fisher Scientific	Cat# PA5-63178; RRID: AB_2644326
Viral E1 antibody	Beijing Protein Innovation	N/A
Viral NSP2 antibody	Abgent	N/A
Rabbit anti-Mouse IgG antibody	Cell Signaling Technology	Cat# 3900; RRID: AB_1550038
HRP-conjugated Goat Polyclonal antibody recognizes Rabbit IgG(H + L)	Arigo	Cat# ARG65351; RRID: AB_2920652
HRP-conjugated Goat Polyclonal antibody recognizes Mouse IgG(H + L)	Arigo	Cat# ARG65350; RRID: AB_2920653
**Bacterial and virus strains**		
psi-LVRU6GP-puro	GeneCopoeia	LVRU6GP
pEZ-Lv157	GeneCopoeia	pEZ-Lv157

pEZ-Lv206	GeneCopoeia	pEZ-Lv206
pRLenti-TRE-EGFP-EF1-rtTA3-IRES-Puro	Obio Technology	GL912
**Biological samples**		
Human osteosarcoma tissue samples	The First Affiliated Hospital of Sun Yat-sen University	N/A
**Chemicals, peptides, and recombinant proteins**		
DMEM	Gibco	11965092
OPTI-MEM	Gibco	31985070
VP-SFM	Gibco	11681020
Fetal Bovine Serum	Gibco	10099-141
TRIzol reagent	Invitrogen	15596018
Penicillin/streptomycin	HyClone	SV30010
Lipofectamine RNAiMAX	Invitrogen	13778-150
Extracellular matrix	Beyotime	C0372
SYBR Premix	Takara	RR820
M-PER Reagent	Thermo Scientific	78501
Protease inhibitor	Roche	05892791001
Phosphatase inhibitor	Roche	4906837001
Immunoprecipitation buffer	Beyotime	P0013
BeyoMag™ Protein A+G magnetic beads	Beyotime	P2108
Bouin's solution	Macklin	B766879
RNase A	Millipore	70856
MG132	Selleck Chemicals	S2619
MLN4924	Selleck Chemicals	S7109
Doxycycline	Selleck Chemicals	S4005
NH_4Cl	Aladdin	A116369
Puromycin	Selleck Chemicals	S7417
Polybrene	Selleck Chemicals	E1299
**Critical commercial assays**		
Qubit™ RNA BR Assay Kit	Invitrogen	Q10210

NEBNext® Poly(A) mRNA Magnetic Isolation Module	NEB	E7490L
NEBNext® Ultra™ II mRNA Library Prep Kit	NEB	E7770L
Qubit™ dsDNA HS Assay Kit	Invitrogen	Q32851
KAPA Library Quant Kit	KAPA	KK4824
NovaSeq S4 reagent kit	llumina	20012866
BCA Protein Assay Kit	Beyotime	P0012
PrimeScript™ RT Reagent Kit	Takara	RR047
EZ-Magna ChIP® HiSens Chromatin Immunoprecipitation Kit	Sigma-aldrich	17-10461
**Deposited data**		
ChIP-seq data	This study	GEO: GSE293855
RNA-seq data	This study	GEO: GSE293856
**Cell lines**		
U2-OS	ATCC	HTB-96
SJSA-1	ATCC	CRL-2098
HOS	ATCC	CRL-1543
143B	ATCC	CRL-8303
MNNG/HOS	ATCC	CRL-1547
G-292	ATCC	CRL-1423
MG-63	ATCC	CRL-1427
U2OS/MTX300	Rizzoli Orthopaedic Institute	DOI: 10.1093/annonc/mdh004
ZOS	The First Affiliated Hospital of Sun Yat-sen University	CVCL_R779
ZOS-M	The First Affiliated Hospital of Sun Yat-sen University	CVCL_R780
ZOSL-1	The First Affiliated Hospital of Sun Yat-sen University	CVCL_B0F2

BHK-21	Cell Bank, Chinese Academy of Sciences	SCSP-5287
Vero	Cell Bank, Chinese Academy of Sciences	SCSP-520
293T	Cell Bank, Chinese Academy of Sciences	SCSP-502
**Experimental models**		
BALB/c nude mice	GemPharmatech Co., Ltd.	D000521
**Oligonucleotides**		
Primers for PCR	GENEray	Table S1 for detailed sequence information
**Software and algorithms**		
GSEA	Broad Institute	http://www.gsea-msigdb.org/gsea/downloads.jsp
Grahpad Prism	Dotmatics	https://www.graphpad.com
SPSS	IBM	https://www.ibm.com/products/spss-statistics
RStudio	RStudio	https://www.rstudio.com/
ImageJ	National Institutes of Health (NIH)	https://imagej.nih.gov/ij
CytExpert	Beckman Coutler	https://www.beckman.tw/flow-cytometry/software
X-tile	Yale School of Medicine	https://medicine.yale.edu/lab/rimm/research/software
