# Supplementary material for: Alphavirus M1 disrupts super-enhancer-driven oncogenic transcription via non-structural protein NSP2 in osteosarcoma
Source: Nat Commun. 2026 Jul 1;17:8208. doi: 10.1038/s41467-026-75013-9 (PMC13463023; doi:10.1038/s41467-026-75013-9)
Supplement: Supplementary file 2 [file 41467_2026_75013_MOESM2_ESM.pdf]

nature portfolio

Corresponding author(s): Weihai Liu, Jing Cai, Jingnan Shen

Last updated by author(s): Feb 21, 2026

Reporting Summary

Nature Portfolio wishes to improve the reproducibility of the work that we publish. This form provides structure for consistency and transparency in reporting. For further information on Nature Portfolio policies, see our Editorial Policies and the Editorial Policy Checklist.

Statistics

For all statistical analyses, confirm that the following items are present in the figure legend, table legend, main text, or Methods section.

n/a	Confirmed	
☐	☒	The exact sample size (*n*) for each experimental group/condition, given as a discrete number and unit of measurement
☐	☒	A statement on whether measurements were taken from distinct samples or whether the same sample was measured repeatedly
☐	☒	The statistical test(s) used AND whether they are one- or two-sided<br>*Only common tests should be described solely by name; describe more complex techniques in the Methods section.*
☒	☐	A description of all covariates tested
☐	☒	A description of any assumptions or corrections, such as tests of normality and adjustment for multiple comparisons
☐	☒	A full description of the statistical parameters including central tendency (e.g. means) or other basic estimates (e.g. regression coefficient) AND variation (e.g. standard deviation) or associated estimates of uncertainty (e.g. confidence intervals)
☐	☒	For null hypothesis testing, the test statistic (e.g. *F*, *t*, *r*) with confidence intervals, effect sizes, degrees of freedom and *P* value noted<br>*Give P values as exact values whenever suitable.*
☒	☐	For Bayesian analysis, information on the choice of priors and Markov chain Monte Carlo settings
☒	☐	For hierarchical and complex designs, identification of the appropriate level for tests and full reporting of outcomes
☐	☒	Estimates of effect sizes (e.g. Cohen's *d*, Pearson's *r*), indicating how they were calculated

Our web collection on statistics for biologists contains articles on many of the points above.

Software and code

Policy information about availability of computer code

Data collection	No customized software was used in this study. The following commercial instruments and associated software were utilized for data acquisition: Microscopy: NIS-Elements Viewer 4.20 (Nikon) was used for image acquisition from microscopy. Cell viability: Data were collected using Gen5 software (BioTek). In vivo fluorescence imaging: iRFP expression in mice was detected using the PerkinElmer IVIS Spectrum system. Flow cytometry: Flow cytometric data were acquired using the CytoFLEX flow cytometer (Beckman Coulter) with CytExpert 2.4 software. qRT-PCR: Real-time PCR data were collected using the 7500 Fast Real-Time PCR System (Applied Biosystems, Life Technologies) and its associated software. Western blotting: Images were captured using the ChemiDoc XRS+ System (Bio-Rad). Transmission electron microscopy (TEM): Performed using a Hitachi HT7800 transmission electron microscope. RNA sequencing: Library preparation and sequencing were performed using the NovaSeq 6000 system (Illumina) and the NovaSeq S4 Reagent Kit. ChIP sequencing: Libraries were prepared using the NEBNext® Ultra™ DNA Library Prep Kit and sequenced on the NovaSeq 6000 platform (Illumina). TMT-based quantitative proteomics (LC-MS/MS): Samples were separated using an Easy-nLC HPLC system and analyzed with a Q-Exactive mass spectrometer. Raw data were searched and quantified using Mascot 2.2 and Proteome Discoverer 1.4. Additional details about software and data analysis are described in the Methods section or are available upon request.
Data analysis	Statistical analyses were conducted using Prism 8.0 (GraphPad), SPSS version 26.0 (SPSS Inc., Chicago, IL, USA), and R version 4.1.3 (The R Foundation for Statistical Computing). Data were obtained from at least three independent experiments and are presented as the mean ± SD. Statistical details of experiments can be found in figure legends. Differences between groups were analyzed using Student's t-test or one-way ANOVA, as appropriate. Kaplan-Meier survival analyses were performed using the log-rank test, and Pearson correlation was used for correlation analyses. A P value < 0.05 was considered statistically significant. In all cases, the p values are represented as follows: *p < 0.05, **p < 0.01, ***p < 0.001 and ****p < 0.0001.

For manuscripts utilizing custom algorithms or software that are central to the research but not yet described in published literature, software must be made available to editors and reviewers. We strongly encourage code deposition in a community repository (e.g. GitHub). See the Nature Portfolio guidelines for submitting code & software for further information.

Data

Policy information about availability of data

All manuscripts must include a data availability statement. This statement should provide the following information, where applicable:

- Accession codes, unique identifiers, or web links for publicly available datasets
- A description of any restrictions on data availability
- For clinical datasets or third party data, please ensure that the statement adheres to our policy

The data supporting the findings of this study are available within the Article, Supplementary Information and Source Data file. Source data are provided with this paper. The ChIP-seq data of osteosarcoma cells pre- and post-M1 virus infection, using anti-H3K27ac and anti-RPB1 antibodies, have been deposited at GEO (GSE293855) [https://www.ncbi.nlm.nih.gov/geo/query/acc.cgi?acc=GSE293855]. RNA-seq data of osteosarcoma cells pre- and post-M1 virus infection, as well as pre- and post-plasmid transfection, have been deposited at GEO (GSE293856) [https://www.ncbi.nlm.nih.gov/geo/query/acc.cgi?acc=GSE293856]. The proteomics data, including TMT-based quantitative proteomics and IP-MS data, have been deposited to the ProteomeXchange Consortium under accession code PXD065368 [https://proteomecentral.proteomexchange.org/?pxid=PXD065368].

Research involving human participants, their data, or biological material

Policy information about studies with human participants or human data. See also policy information about sex, gender (identity/presentation), and sexual orientation and race, ethnicity and racism.

Reporting on sex and gender	This study did not conduct gender analysis.
Reporting on race, ethnicity, or other socially relevant groupings	This study did not involve reporting on race, ethnicity, or other socially relevant groupings.
Population characteristics	The clinical tumor specimens tested in this study were formalin-fixed, paraffin-embedded (FFPE) tissues obtained postoperatively from patients previously treated at the First Affiliated Hospital of Sun Yat-sen University and stored in our tissue archive.
Recruitment	A retrospective analysis was conducted on osteosarcoma tissue specimens from 116 patients, treated at the First Affiliated Hospital of Sun Yat-sen University between August 14, 2003, and May 12, 2010.
Ethics oversight	This study was approved by the Medical Ethics Committee of the First Affiliated Hospital of Sun Yat-sen University (approval ID: [2023]012) and conducted in compliance with the Declaration of Helsinki.

Note that full information on the approval of the study protocol must also be provided in the manuscript.

Field-specific reporting

Please select the one below that is the best fit for your research. If you are not sure, read the appropriate sections before making your selection.

☒ Life sciences ☐ Behavioural & social sciences ☐ Ecological, evolutionary & environmental sciences

For a reference copy of the document with all sections, see nature.com/documents/nr-reporting-summary-flat.pdf

Life sciences study design

All studies must disclose on these points even when the disclosure is negative.

Sample size	For in vitro studies, the experiments were repeated at least 3 times .It is commonly used as the sample size for in vitro studies. For the in vivo experiments,the sample size is determined by the minimum principle of ethical requirements and the tumorigenic capacity of the cancer cells.
Data exclusions	No data were excluded from the data analysis.
Replication	All biological experiments were carried out under clearly defined and standard conditions and the results of all the experiments were generated from at least three biologically independent samples unless noted otherwise in the figure legend.
Randomization	For in vivo experiments , randomizing were performed such that the average tumor sizes were similar across treatment groups. For experiments other than mice experiments, samples were randomly allocated into experimental groups.
Blinding	In in vitro experiments utilizing instrumental readouts such as MTT assay, flow cytometry, and western blot, blinding was not implemented due to the inherent objectivity of the detection instruments. However, in cases of potential bias, such as microscopy analysis or in vivo experiments, data collectors were blinded to the experimental groups.

Reporting for specific materials, systems and methods

We require information from authors about some types of materials, experimental systems and methods used in many studies. Here, indicate whether each material, system or method listed is relevant to your study. If you are not sure if a list item applies to your research, read the appropriate section before selecting a response.

Materials & experimental systems

n/a	Involved in the study
☐	☒ Antibodies
☐	☒ Eukaryotic cell lines
☒	☐ Palaeontology and archaeology
☐	☒ Animals and other organisms
☐	☒ Clinical data
☒	☐ Dual use research of concern
☒	☐ Plants

Methods

n/a	Involved in the study
☐	☒ ChIP-seq
☐	☒ Flow cytometry
☒	☐ MRI-based neuroimaging

Antibodies

Antibodies used

For chromatin immunoprecipitation, the following antibodies were used: anti-H3K27ac antibody (10 μg of antibody in 200 μL chromatin immunoprecipitation reaction, Abcam, Cat# ab4729, RRID: AB_2118291), anti-RPB1 antibody (10 μg of antibody in 200 μL chromatin immunoprecipitation reaction, Abcam, Cat# ab26721, RRID: AB_777726).
For immunoprecipitation, the following antibodies were used: anti-RPB1 antibody (4 μg of antibody per 1 mg protein sample, Abcam, Cat# ab26721, RRID: AB_777726), anti-Viral NSP2 antibody (10 μg of antibody per 1 mg protein sample, Abgent), Rabbit anti-Mouse IgG antibody (4 μg of antibody per 1 mg protein sample, Cell Signaling Technology, Cat# 3900, RRID: AB_1550038).
For Western blotting, the following antibodies were used: anti-RPB1 antibody (dilution 1:1000, Abcam, Cat# ab26721, RRID: AB_777726), anti-GAPDH antibody (dilution 1:1000, Cell Signaling Technology, Cat# 2118S, RRID: AB_561053), anti-Ubiquitin antibody (dilution 1:1000, Cell Signaling Technology, Cat# 43124, RRID: AB_2799235), anti-CUL4A antibody (dilution 1:1000, Cell Signaling Technology, Cat# 2699, RRID: AB_2086563), anti-CUL2 antibody (dilution 1:1000, Proteintech, Cat# 10981-2-AP, RRID: AB_2086422), anti-RBX1 antibody (dilution 1:1000, Proteintech, Cat# 14895-1-AP, RRID: AB_2179719), anti-ELOA antibody (dilution 1:1000, Proteintech, Cat# 10502-1-AP, RRID: AB_2201278), anti-ELOB antibody (dilution 1:2000,Proteintech, Cat# 10779-1-AP, RRID: AB_2240375), anti-ELOC antibody (dilution 1:2000,Proteintech, Cat# 12450-1-AP, RRID: AB_2201139), anti-FLAG antibody (dilution 1:1000, Cell Signaling Technology, Cat# 14793, RRID: AB_2572291), anti-Total/cleaved-PARP antibody (dilution 1:1000, Abcam, Cat# ab191217, RRID: AB_2861274), anti-LDL Receptor antibody (dilution 1:1000, Abcam, Cat# ab52818, RRID: AB_881213), anti-MXRA8 antibody (dilution 1:1000, Thermo Fisher Scientific, Cat# PA5-63178, RRID: AB_2644326), anti-Viral E1 antibody (dilution 1:1000, Beijing Protein Innovation), anti-Viral NSP2 antibody (dilution 1:2000, Abgent), HRP-conjugated Goat Polyclonal antibody recognizes Rabbit IgG(H + L) (dilution 1:5000, Arigo, Cat# ARG65351, RRID: AB_2920652), HRP-conjugated Goat Polyclonal antibody recognizes Mouse IgG(H + L) (dilution 1:5000, Arigo, Cat# ARG65350, RRID: AB_2920653).
For immunohistochemical staining, the following antibodies were used: anti-RPB1 antibody (dilution 1:100, Abcam, Cat# ab26721, RRID: AB_777726).

Validation

Anti-viral NSP2 antibody was customized using the whole protein by Abgent. This is a rabbit polyclonal antibody and well validated by WB and IP. Anti-viral E1 antibody was customized using the whole protein by Beijing Protein Innovation, and was used according to previous experience. This is a mouse monoclonal antibody and well validated by WB. Our research team has demonstrated that the target band is clear in several published papers (DOI: 10.1126/scitranslmed.aam7996; DOI: 10.1073/ pnas.1408759111; DOI: 10.1038/mt.2015.172; DOI: 10.1073/pnas.1701002114;DOI: 10.1128/JVI.01331-17; DOI: 10.1038/s41467-018-03913-6; DOI: 10.1038/s41467-018-06771-4).
Other antibodies used in this study are commercially available and are validated by the manufacturers. Related information is available from their website:
anti-H3K27ac antibody (https://www.abcam.cn/products/primary-antibodies/histone-h3-acetyl-k27-antibody-chip-grade-ab4729.html),
anti-RPB1 antibody (https://www.abcam.cn/products/primary-antibodies/rna-polymerase-ii-ctd-repeat-ysptsps-antibody-chip-grade-ab26721.html),
anti-GAPDH antibody (https://www.cellsignal.cn/products/primary-antibodies/gapdh-14c10-rabbit-mab/2118),
anti-Ubiquitin antibody (https://www.cellsignal.cn/products/primary-antibodies/ubiquitin-e4i2j-rabbit-mab/43124),
anti-CUL4A antibody (https://www.cellsignal.cn/products/primary-antibodies/cul4a-antibody/2699),
anti-CUL2 antibody (https://www.ptgcn.com/products/CUL2-Antibody-10981-2-AP.htm),
anti-RBX1 antibody (https://www.ptgcn.com/products/RBX1-Antibody-14895-1-AP.htm),
anti-ELOA antibody (https://www.ptgcn.com/products/TCEB3-Antibody-10502-1-AP.htm),
anti-ELOB antibody (https://www.ptgcn.com/products/TCEB2-Antibody-10779-1-AP.htm),
anti-ELOC antibody (https://www.ptgcn.com/products/TCEB1-Antibody-12450-1-AP.htm),
anti-FLAG antibody (https://www.cellsignal.cn/products/primary-antibodies/dykddddk-tag-d6w5b-rabbit-mab-binds-to-same-epitope-as-sigma-aldrich-anti-flag-m2-antibody/14793),
anti-Total/cleaved-PARP antibody (https://www.abcam.cn/products/primary-antibodies/parp1-antibody-epr18461-ab191217.html),
anti-LDL Receptor antibody (https://www.abcam.cn/products/primary-antibodies/ldl-receptor-antibody-ep1553y-ab52818),
anti-MXRA8 antibody (https://www.thermofisher.cn/cn/zh/antibody/product/MXRA8-Antibody-Polyclonal/PA5-63178),
Rabbit anti-Mouse IgG antibody (https://www.cellsignal.cn/products/primary-antibodies/rabbit-da1e-mab-igg-xp-isotype-control/3900),
HRP-conjugated Goat Polyclonal antibody recognizes Rabbit IgG(H + L) (https://www.arigobio.cn/Goat-anti-Rabbit-IgG-antibody-HRP-

ARG65351.html),
HRP-conjugated Goat Polyclonal antibody recognizes Mouse IgG(H + L) (https://www.arigobio.cn/Goat-anti-Mouse-IgG-antibody-HRP-ARG65350.html).

Eukaryotic cell lines

Policy information about cell lines and Sex and Gender in Research

Cell line source(s)	Human osteosarcoma cell lines, including U2-OS(Cat# HTB-96), SJSA-1(Cat# CRL-2098), HOS(Cat# CRL-1543), 143B(Cat# CRL-8303), MNNG/HOS(Cat# CRL-1547), G-292(Cat# CRL-1423), and MG-63(Cat# CRL-1427) were obtained from the American Type Culture Collection (ATCC). Methotrexate-resistant U2OS/MTX300 cells, a derivative of the U2-OS osteosarcoma cell line, were generously provided by Dr. M. Serra (Istituti Ortopedici Rizzoli, Bologna, Italy). The ZOS and ZOS-M cell lines, derived from a primary osteosarcoma tumor and its metastasis, respectively, have been described previously (DOI: 10.1111/j.1745-7254.2008.00756.x). Additionally, the ZOSL-1 cell line, established in our laboratory, was derived from a pulmonary metastasis of osteosarcoma (DOI: 10.1016/j.jbo.2021.100378). K7M2(Cat# SCSP-5217), BHK-21(Cat# SCSP-5287), Vero(Cat# SCSP-520), and HEK-293T(Cat# SCSP-502) cells were obtained from Cell Bank, Chinese Academy of Sciences.
Authentication	U2-OS, SJSA-1, HOS, 143B, MNNG/HOS, G-292, and MG-63 were obtained from the American Type Culture Collection (ATCC). K7M2, BHK-21, Vero, and HEK-293T cells were obtained from Cell Bank, Chinese Academy of Sciences. Methotrexate-resistant U2OS/MTX300 cells and primary tumor cell lines (ZOS, ZOSM, ZOSL-1) were authenticated in previously (DOI: 10.1111/j.1745-7254.2008.00756.x, DOI: 10.1016/j.jbo.2021.100378) and frequently checked by their morphological features.
Mycoplasma contamination	All cell lines tested negative for mycoplasma contamination.
Commonly misidentified lines (See ICLAC register)	No commonly misidentified cell lines were used in this study.

Animals and other research organisms

Policy information about studies involving animals; ARRIVE guidelines recommended for reporting animal research, and Sex and Gender in Research

Laboratory animals	Female BALB/c nude mice (4–6 weeks old) and Female BALB/c mice (4–6 weeks old) were all purchased from GemPharmatech Co., Ltd. and housed in a pathogen-free room in the experimental animal center of Sun Yat-sen University.
Wild animals	This study did not involve wild animals.
Reporting on sex	This study did not involve reporting on sex.
Field-collected samples	This study did not involve field-collected samples.
Ethics oversight	All animal studies were approved by the Institutional Review Board of The First Affiliated Hospital of Sun Yat-sen University(approval ID: [2019]022) and conducted in compliance with established guidelines for the Care and Use of Laboratory Animals.

Note that full information on the approval of the study protocol must also be provided in the manuscript.

Clinical data

Policy information about clinical studies

All manuscripts should comply with the ICMJE guidelines for publication of clinical research and a completed CONSORT checklist must be included with all submissions.

Clinical trial registration	*Provide the trial registration number from ClinicalTrials.gov or an equivalent agency.*
Study protocol	*Note where the full trial protocol can be accessed OR if not available, explain why.*
Data collection	*Describe the settings and locales of data collection, noting the time periods of recruitment and data collection.*
Outcomes	*Describe how you pre-defined primary and secondary outcome measures and how you assessed these measures.*

Plants

Seed stocks

Report on the source of all seed stocks or other plant material used. If applicable, state the seed stock centre and catalogue number. If plant specimens were collected from the field, describe the collection location, date and sampling procedures.

Novel plant genotypes

Describe the methods by which all novel plant genotypes were produced. This includes those generated by transgenic approaches, gene editing, chemical/radiation-based mutagenesis and hybridization. For transgenic lines, describe the transformation method, the number of independent lines analyzed and the generation upon which experiments were performed. For gene-edited lines, describe the editor used, the endogenous sequence targeted for editing, the targeting guide RNA sequence (if applicable) and how the editor was applied.

Authentication

Describe any authentication procedures for each seed stock used or novel genotype generated. Describe any experiments used to assess the effect of a mutation and, where applicable, how potential secondary effects (e.g. second site T-DNA insertions, mosiacism, off-target gene editing) were examined.

ChIP-seq

Data deposition

☒ Confirm that both raw and final processed data have been deposited in a public database such as GEO.

☒ Confirm that you have deposited or provided access to graph files (e.g. BED files) for the called peaks.

Data access links
May remain private before publication.

The ChIP-seq data of osteosarcoma cells pre- and post-M1 virus infection, using anti-H3K27ac and anti-RPB1 antibodies, have been deposited at GEO (GSE293855). RNA-seq data of osteosarcoma cells pre- and post-M1 virus infection, as well as pre- and post-plasmid transfection, have been deposited at GEO (GSE293856). These data can be accessed using the following links:
https://www.ncbi.nlm.nih.gov/geo/query/acc.cgi?acc=GSE293855 (Reviewer token=clarkocknhgjrih)
https://www.ncbi.nlm.nih.gov/geo/query/acc.cgi?acc=GSE293856 (Reviewer token=yvqlkawutzklhct)

Files in database submission

ChIP assays were performed on M1 virus-infected or uninfected SJSA-1 cells with anti-H3K27ac or anti-RPB1, respectively. The following H3K27ac ChIP files are included: GSM8891951(negative control, ChIP), GSM8891952 (negative control, INPUT), GSM8891953(M1 virus infected, ChIP), GSM8891954(M1 virus infected, INPUT). The following RPB1 ChIP files are included: GSM8891955(negative control, ChIP), GSM8891956 (negative control, INPUT), GSM8891957(M1 virus infected, ChIP), GSM8891958(M1 virus infected, INPUT). For RNA-seq files, the following samples are included: U2-OS and SJSA-1 cells infected with M1 virus (1 MOI) for 0, 6, 12, and 24 hours or transfected with the Tet-on DOX-inducible NSP2 plasmid, each in triplicate.

Genome browser session
(e.g. UCSC)

Processed ChIP files for all samples have been deposited to GEO with accession number GSE293855 (https://www.ncbi.nlm.nih.gov/geo/query/acc.cgi?acc=GSE293855) and GSE293856 (https://www.ncbi.nlm.nih.gov/geo/query/acc.cgi?acc=GSE293856), for accessibly viewing in a genome browser of choice, respectively.

Methodology

Replicates

Three biological replicates were performed for each cell line (U2-OS, SJSA-1) on RNA-seq. The data were merged before downstream analysis. ChIP samples were not replicated.

Sequencing depth

ChIP-seq libraries were sequenced on the Illumina NovaSeq 6000 platform using 150 bp paired-end reads (PE150). Each sample generated approximately 12 GB of sequencing data, corresponding to an average of ~40 million reads per sample, with variation across samples being less than 10%.

Antibodies

H3K27ac ChIP-seq antibody (Cat# ab4729) and RPB1 ChIP-seq antibody (Cat# ab26721) for ChIP experiments was purchased from Abcam.

Peak calling parameters

Peak calling was performed using MACS2 (v2.1.2) with the parameters -m 5 50. Constituent enhancer peaks were defined based on H3K27ac or RPB1 enrichment. Super-enhancers were identified using the ROSE algorithm with a stitching distance of 12,500 bp, excluding peaks within ±2,500 bp of annotated transcription start sites (TSS).

Data quality

Sequencing data quality was assessed using standard filtering steps. Adapters and low-quality reads were trimmed using Cutadapt (v2.5). Reads were aligned to the hg38 (or mm10 for mouse samples) genome using Bowtie2 (v2.3.5.1) with default parameters. All samples yielded sufficient coverage (~40 million reads), and biological replicates showed consistent enrichment profiles, supporting high-confidence peak identification and downstream super-enhancer analysis.

Software

The following tools were used in the ChIP-seq analysis pipeline:
Cutadapt (v2.5) for adapter trimming
Bowtie2 (v2.3.5.1) for read alignment
MACS2 (v2.1.2) for peak calling
ROSE (https://bitbucket.org/young_computation/rose) for super-enhancer identification
HOMER (v4.10.4) for enhancer annotation using annotatePeaks.pl
clusterProfiler (v3.6.0) in R for Gene Ontology and pathway analysis
MEME (v5.1.1) for motif discovery in super-enhancer peaks

Flow Cytometry

Plots

Confirm that:

☒ The axis labels state the marker and fluorochrome used (e.g. CD4-FITC).

☒ The axis scales are clearly visible. Include numbers along axes only for bottom left plot of group (a 'group' is an analysis of identical markers).

☒ All plots are contour plots with outliers or pseudocolor plots.

☒ A numerical value for number of cells or percentage (with statistics) is provided.

Methodology

Sample preparation	U2-OS and SJSA-1 cells were infected with M1viruses for 0, 6, 12, 24h, and the infection rate was determined by detecting the expression of GFP during viral replication.
Instrument	CytoFLEX, Beckman Coulter
Software	CytExpert 2.4
Cell population abundance	The purity of sorted cells was over 99% because there's no sorting or purification.
Gating strategy	To determine GFP expression in tumor cells, live single cells were further gated on FSC-A/SSC-A, FSC-A/FSC-H, and then the expression level of GFP was detected using FITC channels.

☒ Tick this box to confirm that a figure exemplifying the gating strategy is provided in the Supplementary Information.
