# Supplementary material for: Alphavirus M1 disrupts super-enhancer-driven oncogenic transcription via non-structural protein NSP2 in osteosarcoma
Source: Nat Commun. 2026 Jul 1;17:8208. doi: 10.1038/s41467-026-75013-9 (PMC13463023; doi:10.1038/s41467-026-75013-9)
Supplement: Supplementary file 3 [file 41467_2026_75013_MOESM3_ESM.pdf]

nature portfolio

Peer Review File

Alphavirus M1 disrupts super-enhancer-driven oncogenic transcription via non-structural protein NSP2 in osteosarcoma

Corresponding Author: Dr Jiajun Zhang

This file contains all reviewer reports in order by version, followed by all author rebuttals in order by version.

Version 0:

Reviewer comments:

Reviewer #1

(Remarks to the Author)
This study investigates the mechanisms underlying the oncolytic activity of the M1 virus in osteosarcoma. The authors demonstrate that M1 virus disrupts super-enhancer activity in tumors by downregulating RPB1. This effect is mediated by the viral protein NSP2, which recruits the CRL2 complex and induces K63-linked ubiquitination of the RPB1 subunit. The study is relevant and timely, as it explores the potential of M1 as an anti-tumor agent and provides a detailed mechanistic insight into a novel oncolytic mechanism.
Major Concerns:
1. In Vivo Anti-Tumor Efficacy:
My main concern is the modest in vivo anti-tumor effect shown in Figure 2, despite 14 consecutive intravenous administrations of the virus. Moreover, there is an inconsistency in the mouse model: the text describes the animals as nude mice, while the Materials and Methods section and the figure legend refer to Balb/C mice. Regardless of this discrepancy, the current data are not sufficient to support robust in vivo efficacy. I strongly recommend testing the therapeutic effect in an additional, well-characterized in vivo model.
2. Virus Dosing Strategy:
In the IVIS study, viral persistence in tumors is demonstrated 4 days post-injection. Given this, it is unclear why the therapeutic experiment required 14 daily virus administrations. Is such frequent dosing necessary to achieve efficacy? Additional explanation or justification for this intensive regimen would strengthen the study.
3. ER-Stress Evaluation:
While the microscopy data related to ER stress are visually informative, they should be supported by more objective molecular measurements. Assessing the expression of ER-stress sensor proteins or other relevant markers would provide stronger evidence for the conclusions drawn.
Minor Comments:
• Please clarify which 70 epithelial tumor cell lines were used in Figure 1f and 1g. A table or supplementary list would be helpful.
tumor cell lines were used in Figure 1f and 1g. A table or supplementary list would be helpful.

Reviewer #2

(Remarks to the Author)
In this manuscript, Zhang and colleagues report that the oncolytic alphavirus M1 has strong cytolytic activity in OS cells and identify an "OS-specific" mechanism whereby M1 NSP2 leads to the degradation of RPB1 and disruption of oncogenic super enhancer function.

While intruiging, there are several experimental shortcomings that limit my overall enthusiasm:

1.) the activity of M1 against OS in vivo must be assessed using immunocompetent models. Too many OVs have fallen short because their activity was assessed solely in xenografts.

2.) the mechanism identified must be validated in vivo, using immunocompetent model systems. Does M1 productively infect OS cells in a complex TME and kill them using this mechanism?

3.) what is the breadth of this mechanism? Is it unique to M1, or do other OVs possess it in OS cells?

Reviewer #3

(Remarks to the Author)
This study uncovers a novel therapeutic mechanism of the natural alphavirus M1 in osteosarcoma, a cancer with limited treatment options and poor prognosis. The researchers demonstrate that M1 virus selectively infects and suppresses osteosarcoma cells both in vitro and in vivo, not primarily through the usual ER stress pathway, but by disrupting the activity of oncogenic super-enhancers—genomic regions that drive cancer gene expression. The viral non-structural protein NSP2 binds to the transcription factor RPB1 and recruits the host's CUL2-RBX1-ELOC E3 ligase complex to induce K63-linked ubiquitination and degradation of RPB1. This degradation shuts down super-enhancer-driven transcription, leading to tumor growth inhibition and reduced metastasis. The study highlights RPB1 as a potential biomarker for predicting response to M1 virotherapy and positions M1 as a promising candidate for treating osteosarcoma through epigenetic interference rather than traditional cytotoxicity.
Figures 3 to end are the strength of the paper; they are well-designed and easy to follow. However, the characterization of the M1 virus in Figures 1 and 2 is a major weakness.
While the mechanism identified in this study is interesting, its translational potential is unclear. In a clinical setting, only a small fraction of tumor cells would likely be infected, so durable efficacy would depend on inducing an immune response capable of eliminating uninfected cells. The use of immunocompetent mouse models would be critical to bridge this gap and clarify whether the M1-induced changes can trigger effective anti-tumor immunity. If there is any benefit observed can be reversed by targeting the key biomarkers found in this study? If any therapeutic benefit is observed, can it be reversed by targeting the key biomarkers identified in this study?
Additional comments:
• In Figure 1A, the replication kinetics of M1 are unclear. A virus growth curve across multiple MOIs is needed to demonstrate replication efficiency.
• In Figure 1E, it is unclear why certain osteosarcoma lines are resistant. Is this due to impaired viral entry (e.g., lack of receptor), reduced replication, or failure to induce cytotoxicity despite replication? These questions could be addressed using M1-GFP tracking and viral titration over time. The mechanism of viral entry should also be discussed in the introduction, including any known receptors. How does infectivity compare between human and mouse cell lines? It is known that VSV, VV, and HSV exhibit reduced infectivity in murine models.
• In Figure 2I, immunohistochemistry (IHC) for viral proteins in tumor tissue should be included to confirm virus delivery. This would also serve as a positive control for IHC sensitivity.
• The xenograft model used in Figure 2 is not clearly described. More information should be provided in the main text or methods.
• The use of iRFP for in vivo imaging is suboptimal due to its lower sensitivity compared to luciferase. For biodistribution, viral titration remains the gold standard, although qPCR is an acceptable complementary method.
• Details of virus production and titration methods are missing from the Materials and Methods and should be completed.
• The virus used in this study should be sequenced, and the full genome sequence should be included in the supplementary materials.

Version 1:

Reviewer comments:

Reviewer #1

(Remarks to the Author)
The revised manuscript presents an interesting and well developed mechanistic story in which alphavirus M1, via NSP2, recruits a CUL2, RBX1, and ELOC complex to induce RPB1 degradation and thereby disrupt super enhancer driven oncogenic transcription in osteosarcoma. The multi omics and cell based data are generally strong and internally consistent, and the additional experiments, including IHC for RPB1 and Ki 67 in vivo, comparison with other oncolytic viruses, and characterization of infection and receptor expression, clearly strengthen the mechanistic aspects of the work.

However, despite these improvements, I remain unconvinced that the in vivo and translational data fully support the strength of the claims made in the manuscript. My main concerns are outlined below.

In vivo antitumor efficacy remains modest and model limited

The core efficacy data still come from a single human osteosarcoma xenograft model in nude mice and a single murine osteosarcoma model in immunocompetent mice, both treated with very intensive daily intravenous dosing over 14 days. Tumor growth is delayed during treatment, but tumors regrow promptly after cessation in the xenograft setting, and the overall magnitude of benefit does not justify the repeated description of "remarkable" or "potent" in vivo efficacy. The new immunocompetent model data are valuable, but remain limited in scope, involving one cell line, one orthotopic site, and relatively short follow up. As a result, questions regarding robustness, durability of response, and generalizability remain

insufficiently addressed.

Dosing regimen and translational relevance are not resolved

The authors argue that daily dosing is intended to maintain therapeutic viral levels prior to the development of neutralizing antibodies. While this is biologically plausible, it remains largely speculative, as no data are presented to define intratumoral viral kinetics under different schedules, nor is there any attempt to explore a less intensive but still effective regimen. At the same time, the manuscript repeatedly emphasizes strong translational potential, despite acknowledging that the dosing schedule used here would not be clinically feasible. This disconnect between the regimen actually tested and the language used to describe therapeutic potential remains an important weakness.

Scope of the in vivo mechanism and immune contribution

The additional IHC demonstrating reduced RPB1 and Ki 67 in E1 positive regions provides useful confirmation that the proposed mechanism operates in vivo, and the comparison with HSV 1 and VSV supports some degree of specificity. However, the study still offers only a limited exploration of how this mechanism interfaces with antitumor immunity in osteosarcoma. Given that clinical benefit from oncolytic virotherapy typically depends in part on immune mediated clearance of uninfected tumor cells, the current data do not convincingly bridge the gap between an elegant cell intrinsic mechanism and a realistic therapeutic effect in an immunocompetent setting.

Translational claims appear overstated relative to the data

Throughout the manuscript, the authors use strong language about “remarkable anti tumor efficacy,” “potent in vivo and in vitro anti osteosarcoma effect,” “favorable safety,” and direct clinical translatability, including positioning M1 as a new strategy for super enhancer targeted therapy. In my view, the preclinical evidence presented, two models, one very intensive treatment schedule, modest and non durable tumor control, limited immune characterization, and acknowledged resistance in some cell lines, does not fully support this level of extrapolation. A more cautious framing, for example presenting the work primarily as mechanistic and hypothesis generating, and as a basis for future optimization of dosing or combination strategies, would feel more appropriate.

Taken together, I think the study contains interesting and potentially important mechanistic insights. However, the in vivo and translational aspects remain limited and somewhat over interpreted.

Reviewer #3

(Remarks to the Author)
I dont have any further comments. The authors have addressed my concerns well.

Open Access This Peer Review File is licensed under a Creative Commons Attribution 4.0 International License, which permits use, sharing, adaptation, distribution and reproduction in any medium or format, as long as you give appropriate credit to the original author(s) and the source, provide a link to the Creative Commons license, and indicate if changes were made.

In cases where reviewers are anonymous, credit should be given to 'Anonymous Referee' and the source.
The images or other third party material in this Peer Review File are included in the article's Creative Commons license, unless indicated otherwise in a credit line to the material. If material is not included in the article's Creative Commons license and your intended use is not permitted by statutory regulation or exceeds the permitted use, you will need to obtain permission directly from the copyright holder.
To view a copy of this license, visit https://creativecommons.org/licenses/by/4.0/

Revised Response to Reviewers

**Manuscript #**:	NCOMMS-25-43655
**Title**:	Alphavirus M1 disrupts super-enhancer-driven oncogenic transcription via non-structural protein NSP2 in osteosarcoma

Responds to Reviewer #1:

Dear reviewer:

We appreciate very much for your positive and constructive comments on our manuscript entitled 'Alphavirus M1 disrupts super-enhancer-driven oncogenic transcription via non-structural protein NSP2 in osteosarcoma'. We have studied these comments carefully and have made revision which marked in the tracked-changes version. We have tried our best to revise our manuscript according to the comments, and hope that the correction will meet with approval.

Major Concerns:
1. In Vivo Anti-Tumor Efficacy:
My main concern is the modest in vivo anti-tumor effect shown in Figure 2, despite 14 consecutive intravenous administrations of the virus. Moreover, there is an inconsistency in the mouse model: the text describes the animals as nude mice, while the Materials and Methods section and the figure legend refer to Balb/C mice. Regardless of this discrepancy, the current data are not sufficient to support robust in vivo efficacy. I strongly recommend testing the therapeutic effect in an additional, well-characterized in vivo model.

Thank you for your insightful comments, which greatly help us improve the quality of our work. We address the concerns as follows:

First, during the 14-day period of continuous viral administration in both osteosarcoma cell–derived xenografts, we indeed observed notable tumor inhibition. However, during the 7-day observation period after treatment cessation, tumor regrowth became evident. This phenomenon may be attributed to innate and humoral immune components still present in nude mice, which can mediate viral clearance. Previous studies have also reported the kinetics of viral persistence and clearance in mice[1].

Second, we used Balb/c-background nude mice to ensure a stable genetic background while leveraging the immunodeficient phenotype to facilitate tumor growth monitoring and live imaging. This model is also among the most commonly used in osteosarcoma research[2-4]. Any unclear wording in the manuscript has now been corrected.

Finally, during revision, we have initiated additional experiments using immunocompetent mouse models to evaluate the therapeutic efficacy of M1 virus under intact immune conditions. These data has been provided as Extended Data Fig. 3. Notably, M1 virus exhibited significant inhibition of tumor growth and lung metastasis (Extended Data Fig. 3e-k), as well as tumor-selective

enrichment (Extended Data Fig. 3c-d), in these immunocompetent models, consistent with our observations in xenografts.

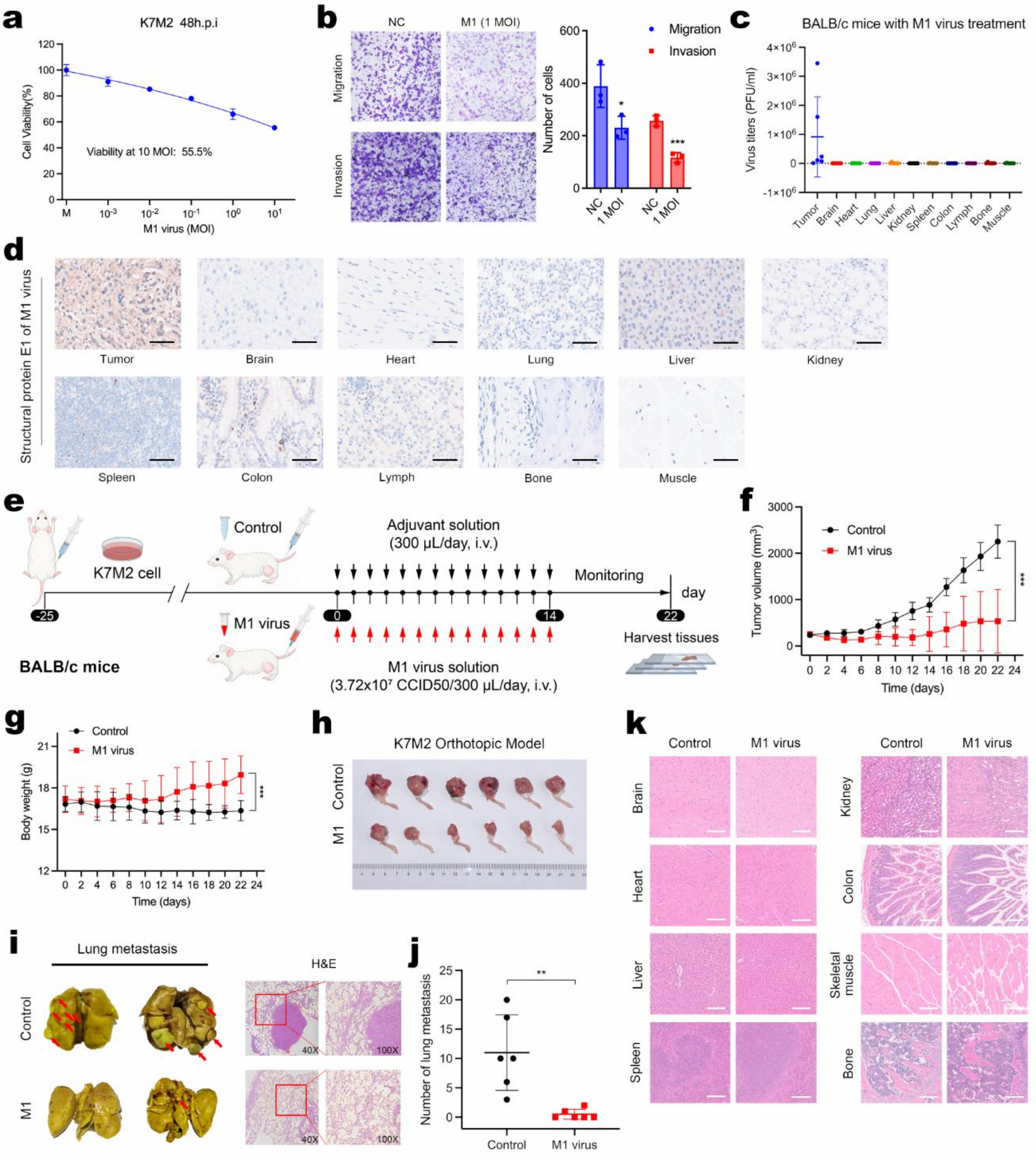

Extended Data Fig. 3| Antitumor activity and biodistribution of M1 virus in an immunocompetent murine osteosarcoma model

a, Viability of murine cell line K7M2 after infection with M1 virus at indicated MOIs for 48 hours. **b**, Representative images of K7M2 cell migration and invasion assay after treatment with M1 virus (1 MOI) or NC (negative control). **c**, Viral titers (plaque-forming units, PFU) in indicated tissues harvested from BALB/c mice on day 4 post-administration of M1 virus. Data are presented as PFU per mL of tissue homogenate. n = 6. **d**, Representative immunohistochemistry (IHC) images showing the expression of the M1 virus E1 protein in various tissues from treated mice. Scale bar, 50 μm. **e**, Schematic illustration of the experimental timeline for the in vivo therapeutic study. K7M2 cells were implanted into the tibia of BALB/c mice to establish an orthotopic model, followed by intravenous

administration of M1 virus or vehicle control. **f**,**g**, Tumor volume (**f**) and body weight (**g**) changes of tumor-bearing mice during the treatment period. Data are presented as mean ± SD (n=6 mice per group). **h**, Images of orthotopic tibial tumors from the control and M1 virus-treated groups at the experimental endpoint. **i**, Representative lung tissue images showing metastatic nodules (indicated by red arrows) (**i**, left). H&E images were taken at magnification of 40× and 100× (**i**, right). **j**, Quantification of lung metastatic foci from (**i**). n = 6. **k**, Representative H&E images revealed no tissue damage in the brain, heart, liver, spleen, kidneys, colon, skeletal muscle, or bone in M1 treated mice. These data are presented as the means ± SD and were analyzed by two-way ANOVA (**f, g**), and unpaired Student's t test (**b, j**). *, P < 0.05; **, P < 0.01; ***, P < 0.001.

Reference:

[1] Zhu W, Liang J, Tan J, Guo L, Cai J, Hu J, Yan G, Liu Y, Zhang J, Song D, Dan J, Wong CW, Su X, Qiu P, Lin Y. Real-Time Visualization and Quantification of Oncolytic M1 Virus In Vitro and In Vivo. Hum Gene Ther. 2021 Feb;32(3-4):158-165. doi: 10.1089/hum.2020.273.

[2] Zou Q, Zhou X, Lai J, Zhou H, Su J, Zhang Z, Zhuang X, Liu L, Yuan R, Li S, Yang S, Qu X, Feng J, Liu Y, Li Z, Huang S, Shi Z, Yan Y, Zheng Z, Ye W, Qi Q. Targeting p62 by sulforaphane promotes autolysosomal degradation of SLC7A11, inducing ferroptosis for osteosarcoma treatment. Redox Biol. 2025 Feb;79:103460. doi: 10.1016/j.redox.2024.103460. Epub 2024 Dec 5.

[3] Zeng J, Yan Z, Wang D, He T, Tong Z, Miao J, Li J, Tan W, Chen S, Deng Y. Mitochondria-targeted MXene@MnO2-TPP nanoheterostructures for synergistic enhancement of sonodynamic therapy and immunotherapy in osteosarcoma. Bioact Mater. 2025 Aug 28;54:450-465. doi: 10.1016/j.bioactmat.2025.08.029.

[4] Zheng D, Liu W, Xie W, Huang G, Jiang Q, Yang Y, Huang J, Xing Z, Yuan M, Wei M, Li Y, Yin J, Shen J, Shi Z. AHA1 upregulates IDH1 and metabolic activity to promote growth and metastasis and predicts prognosis in osteosarcoma. Signal Transduct Target Ther. 2021 Jan 20;6(1):25. doi: 10.1038/s41392-020-00387-1.

2. Virus Dosing Strategy:
In the IVIS study, viral persistence in tumors is demonstrated 4 days post-injection. Given this, it is unclear why the therapeutic experiment required 14 daily virus administrations. Is such frequent dosing necessary to achieve efficacy? Additional explanation or justification for this intensive regimen would strengthen the study.

We appreciate this important question. Although IVIS imaging shows that viral signals can remain detectable for up to four days, persistence of fluorescence does not directly reflect whether the replicating viral load remains above a therapeutic threshold throughout the treatment period.

In mice, strong neutralizing antibody responses typically arise within 5–7 days after first exposure. Once neutralizing antibodies are generated, subsequent systemic dosing may become ineffective.

Prior work has shown that M1 virus reaches peak intratumoral titers around day 2 following intravenous administration, after which levels decline[1].

Therefore, our daily–for–14-days regimen was designed based on maintaining a therapeutic time-window and ensuring sustained infection before neutralizing antibodies develop. We agree that from a clinical translation perspective, daily intravenous administration is not feasible. The primary aim of this study is to serve as a proof of concept, exploring the maximum therapeutic potential of this virus in an aggressive model. We have now clearly demonstrated the significant efficacy of the virus under this intensive dosing regimen, providing robust support for its effectiveness. Our future work will focus on optimizing the dosing strategy. Thank you once again for your insightful comments.

Reference:

[1] Zhu W, Liang J, Tan J, Guo L, Cai J, Hu J, Yan G, Liu Y, Zhang J, Song D, Dan J, Wong CW, Su X, Qiu P, Lin Y. Real-Time Visualization and Quantification of Oncolytic M1 Virus In Vitro and In Vivo. Hum Gene Ther. 2021 Feb;32(3-4):158-165. doi: 10.1089/hum.2020.273.

3. ER-Stress Evaluation:
While the microscopy data related to ER stress are visually informative, they should be supported by more objective molecular measurements. Assessing the expression of ER-stress sensor proteins or other relevant markers would provide stronger evidence for the conclusions drawn.

We supplemented the study with Western blot analyses of ER-stress markers BiP and CHOP in U2-OS and SJSA-1 cells after M1 infection over time. M1 infection did not significantly elevate these markers compared to the positive control (Brefeldin A)(Extended Data Fig. 4a). We also examined these markers across 11 OS cell lines and found no strong correlation with cytotoxicity (Extended Data Fig. 4b). These findings suggest that M1 virus infection induces ER perturbation in osteosarcoma cells but does not trigger a significant ER stress response or subsequent activation of apoptotic pathways.

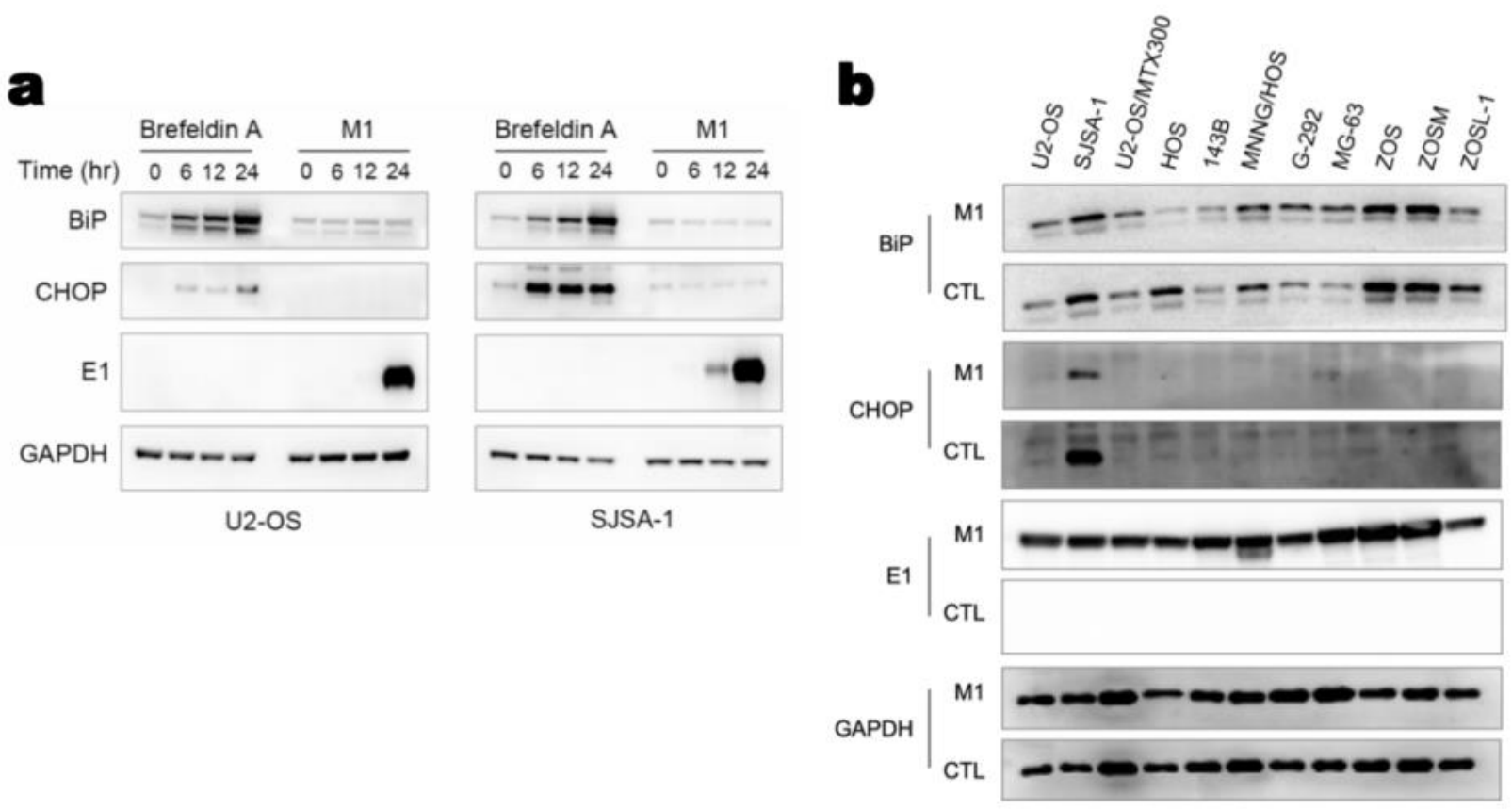

Extended Data Fig. 4| Limited induction of canonical ER stress by M1 in osteosarcoma cells, related to Fig. 3

a, U2-OS and SJSA-1 cells were infected with M1 for the indicated times. Brefeldin A (BFA) served as a positive control. Western blot analysis of ER-stress markers (BiP and CHOP) showed robust induction by BFA but no significant elevation following M1 infection (Viral E1 confirmed infection). **b**, ER-stress markers were further examined in 11 osteosarcoma cell lines. No consistent upregulation of BiP or CHOP was observed after M1 infection (Viral E1 confirmed infection).

Minor Comments:

• Please clarify which 70 epithelial tumor cell lines were used in Figure 1f and 1g. A table or supplementary list would be helpful.

We corrected the description of the 70 tumor cell lines in Figure 1f and 1g and have provided the full list as Table S1.

Responds to Reviewer #2:

Dear reviewer:

We appreciate very much for your positive and constructive comments on our manuscript entitled 'Alphavirus M1 disrupts super-enhancer-driven oncogenic transcription via non-structural protein NSP2 in osteosarcoma'. We have studied these comments carefully and have made revision which marked in the tracked-changes version. We have tried our best to revise our manuscript according to the comments, and hope that the correction will meet with approval.

1.) the activity of M1 against OS in vivo must be assessed using immunocompetent models. Too many OVs have fallen short because their activity was assessed solely in xenografts.

<u>Thank you for your insightful comments, which greatly help us improve the quality of our work. We established a tibial osteosarcoma model using K7M2 cells in immunocompetent Balb/c mice and are currently assessing the therapeutic effects and biodistribution of M1 virus in this setting. These data has been provided as Extended Data Fig. 3. Notably, M1 virus exhibited significant inhibition of tumor growth and lung metastasis (Extended Data Fig. 3e-k), as well as tumor-selective enrichment (Extended Data Fig. 3c-d), in these immunocompetent models, consistent with our observations in xenografts.</u>

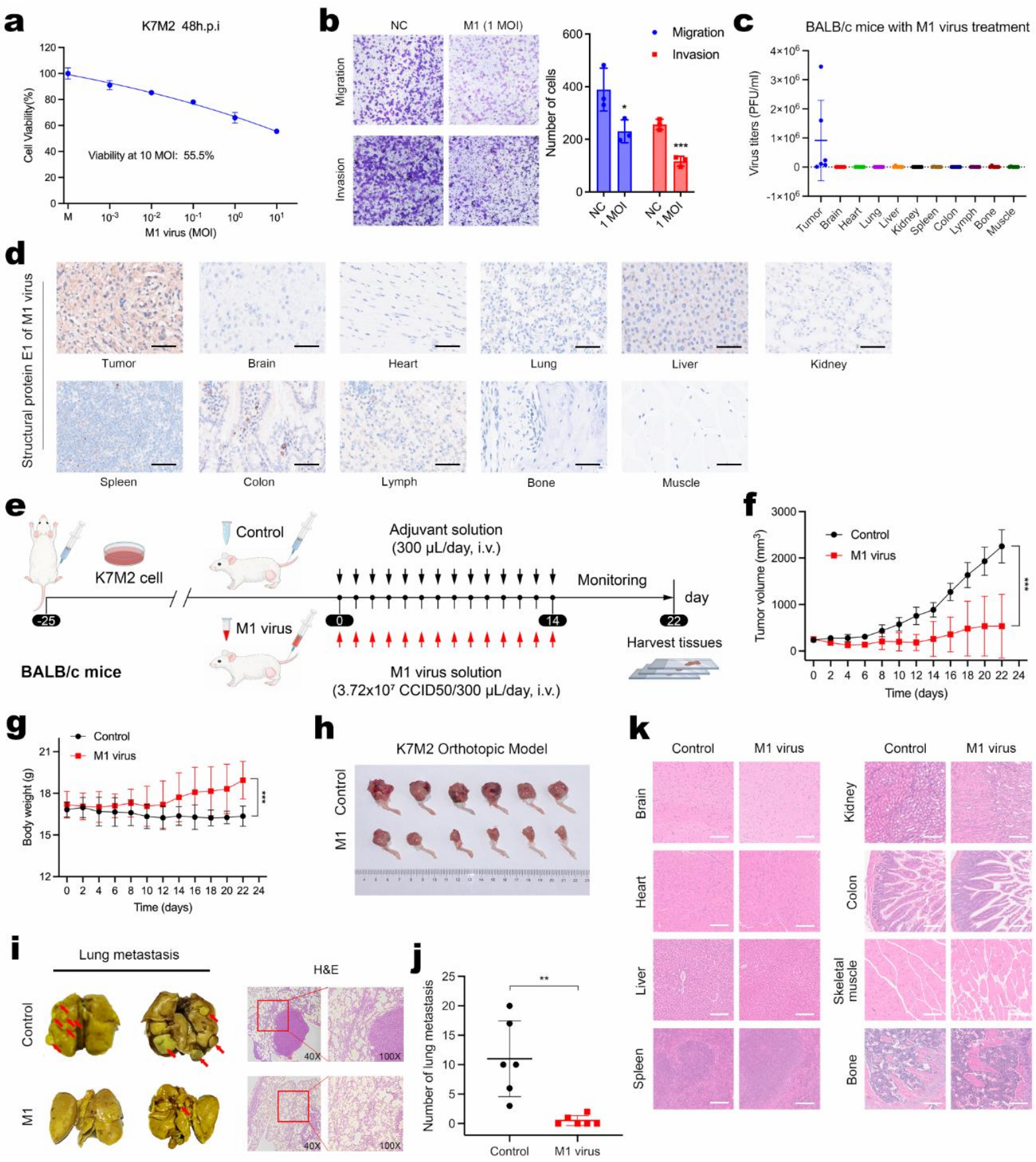

<u>Extended Data Fig. 3| Antitumor activity and biodistribution of M1 virus in an immunocompetent murine osteosarcoma model</u>

a, Viability of murine cell line K7M2 after infection with M1 virus at indicated MOIs for 48 hours. **b**, Representative images of K7M2 cell migration and invasion assay after treatment with M1 virus (1 MOI) or NC (negative control). **c**, Viral titers (plaque-forming units, PFU) in indicated tissues harvested from BALB/c mice on day 4 post-administration of M1 virus. Data are presented as PFU per mL of tissue homogenate. n = 6. **d**, Representative immunohistochemistry (IHC) images showing the expression of the M1 virus E1 protein in various tissues from treated mice. Scale bar, 50 μm. **e**, Schematic illustration of the experimental timeline for the in vivo therapeutic study. K7M2 cells were implanted into the tibia of BALB/c mice to establish an orthotopic model, followed by intravenous administration of M1 virus or vehicle control. **f**,**g**, Tumor volume (**f**) and body weight (**g**) changes of tumor-bearing mice during the treatment period. Data are presented as mean ± SD (n=6 mice per group). **h**, Images of orthotopic tibial tumors from the control and M1 virus-treated groups at the experimental endpoint. **i**, Representative lung tissue images showing metastatic nodules (indicated by red arrows) (**i**, left). H&E images were taken at magnification of 40× and 100× (**i**, right). **j**, Quantification of lung metastatic foci from (**i**). n = 6. **k**, Representative H&E images revealed no tissue damage in the brain, heart, liver, spleen, kidneys, colon, skeletal muscle, or bone in M1 treated mice. These data are presented as the means ± SD and were analyzed by two-way ANOVA (**f, g**), and unpaired Student's t test (**b, j**). *, $P < 0.05$; **, $P < 0.01$; ***, $P < 0.001$.

2.) the mechanism identified must be validated in vivo, using immunocompetent model systems. Does M1 productively infect OS cells in a complex TME and kill them using this mechanism?

Thank you for this insightful comment. In response to your suggestion, we performed further validation using tumor tissue collected from immunocompetent murine osteosarcoma models treated with the M1 virus. Specifically, we analyzed protein levels of RPB1 in these samples to assess whether the same antitumor mechanism operates within the complex tumor microenvironment (TME). These data has been provided as Extended Data Fig. 11. Immunohistochemical analysis of tumor specimens from both nude mice and immunocompetent mouse models treated with M1 virus revealed that within M1 virus-infected regions (E1-positive areas), both RPB1 and Ki-67 levels were markedly decreased.

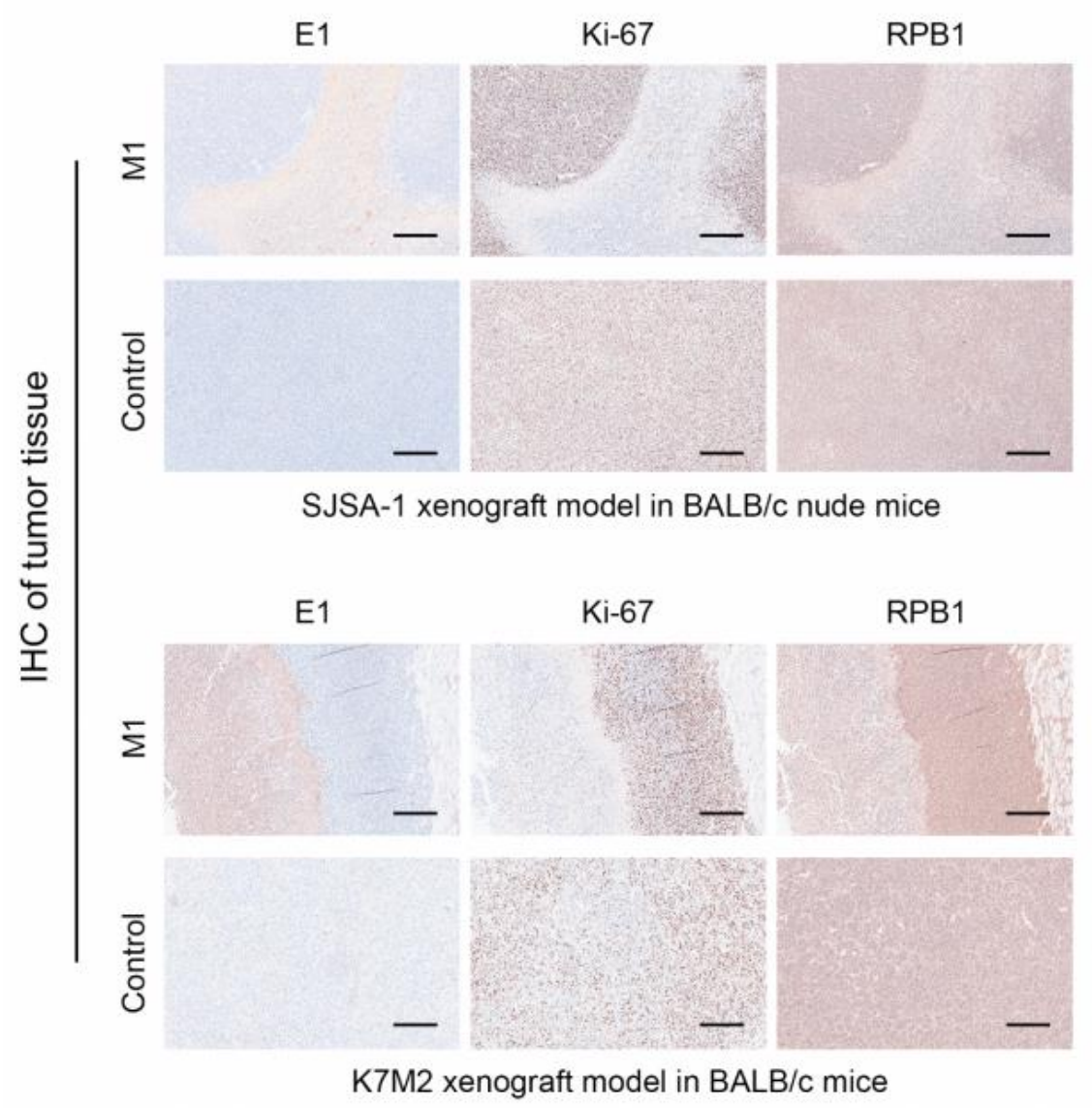

Extended Data Fig. 11 M1 virus downregulates RPB1 expression and suppresses proliferation in osteosarcoma xenografts, related to Fig. 5

Representative immunohistochemistry (IHC) images of tumor tissues from SJSA-1 orthotopic xenografts in BALB/c nude mice (**upper panel**) and K7M2 orthotopic xenografts in immunocompetent BALB/c mice (**lower panel**). Mice were treated with either control vehicle or M1 virus, and tumor tissues were harvested on day 4 post-administration. Sections were stained for the M1 viral protein E1 (infection marker), the proliferation marker Ki-67, and RPB1. Scale bar, 300 µm.

3.) what is the breadth of this mechanism? Is it unique to M1, or do other OVs possess it in OS cells?

Thank you for your insightful question regarding the breadth of the observed mechanism. This is a highly valuable point. We agree that investigating whether the RPB1-SE changes we observed are unique to M1 or represent a more general phenomenon is meaningful. To address it, we selected two oncolytic viruses (OVs) with genomic structures distinct from M1 virus (+ssRNA): HSV-1 (dsDNA) and VSV (-ssRNA), both of which are well-established in oncolytic virotherapy research. U2-OS cells were infected with these viruses (Figure a), and Western blot analysis showed that RPB1 protein levels remained unchanged following HSV-1 or VSV infection, in contrast to the marked downregulation observed with M1 virus (Figure b). These findings imply that the RPB1-mediated mechanism may be specific to M1 virus.

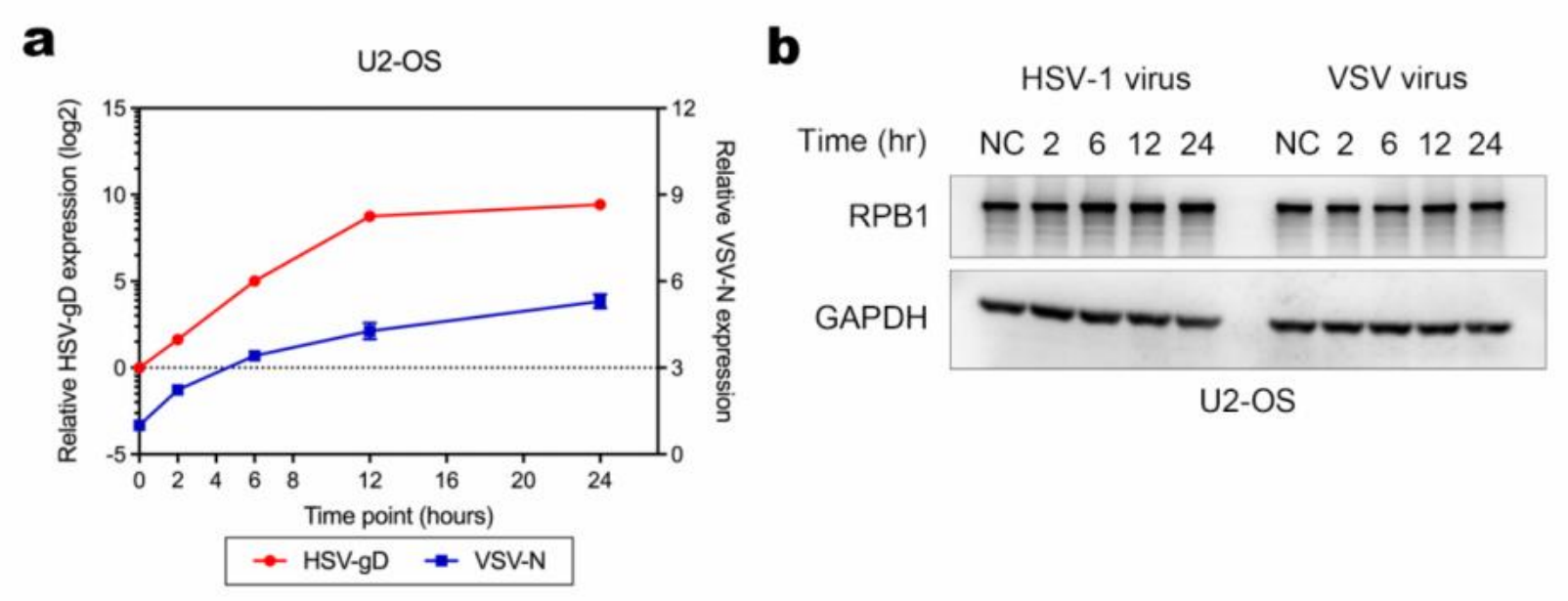

Responds to Reviewer #3:

Dear reviewer:

We appreciate very much for your positive and constructive comments on our manuscript entitled 'Alphavirus M1 disrupts super-enhancer-driven oncogenic transcription via non-structural protein NSP2 in osteosarcoma'. We have studied these comments carefully and have made revision which marked in the tracked-changes version. We have tried our best to revise our manuscript according to the comments, and hope that the correction will meet with approval.

Figures 3 to end are the strength of the paper; they are well-designed and easy to follow. However, the characterization of the M1 virus in Figures 1 and 2 is a major weakness.
While the mechanism identified in this study is interesting, its translational potential is unclear. In a clinical setting, only a small fraction of tumor cells would likely be infected, so durable efficacy would depend on inducing an immune response capable of eliminating uninfected cells. The use of immunocompetent mouse models would be critical to bridge this gap and clarify whether the M1-induced changes can trigger effective anti-tumor immunity. If there is any benefit observed can be reversed by targeting the key biomarkers found in this study? If any therapeutic benefit is observed, can it be reversed by targeting the key biomarkers identified in this study?

Thank you for your professional feedback, which is instrumental in helping us enhance the quality of our research. In this study, we used immunodeficient nude mice to establish a xenograft model of human osteosarcoma. This model helps, to some extent, to exclude the influence of an M1-induced immune response on anti-tumor efficacy, thereby allowing us to focus on the anti-osteosarcoma effects mediated by M1 through non-immune pathways. That said, we fully agree that to comprehensively evaluate the in vivo efficacy of oncolytic virus therapy, studies in immunocompetent animal models are essential.

In response to this important point, we established a tibial orthotopic tumor model using the murine osteosarcoma cell line K7M2 in immunocompetent Balb/C mice. These data has been provided as Extended Data Fig. 3. In these immunocompetent models, we similarly observed significant inhibitory effects of M1 virus on both primary osteosarcoma tumor growth and lung metastasis (Extended Data Fig. 3e-k). Furthermore, substantial tumor-selective enrichment of M1 virus was also detected within the tumor tissues (Extended Data Fig. 3c-d). Additional IHC analysis revealed that within M1 virus-infected regions of the tumor, both RPB1 protein levels and Ki-67 positivity were markedly decreased in tumor cells (Extended Data Fig. 11). These findings demonstrate that M1 virus exerts a potent in vivo anti-osteosarcoma effect through targeting RPB1.

Activation of the antitumor immune response of M1 virus has been extensively investigated in the context of intravenous injection. M1 induces immunogenic tumor cell death and subsequently resumes priming of antitumor T cells by dendritic cells. Moreover, intravenous injection of M1 virus establishes long-term antitumor immune memory in poorly immunogenic tumor models [1, 2]. In the present study, we have identified a novel direct tumor-killing mechanism by which M1 virus targets and inhibits tumor-associated super-enhancers through inducing RPB1 protein degradation in host cells. However, given the complexity and multiplicity of tumor-associated

super-enhancers aberrantly activated by RPB1, their interplay with tumor immunity remains intricate and not fully understood. Nevertheless, this is indeed a worthwhile avenue for further exploration, and our team is currently initiating preliminary investigations in this direction, with the hope of uncovering new and interesting findings to share in the future.

[1] Liu Y, Cai J, Liu W, Lin Y, Guo L, Liu X, Qin Z, Xu C, Zhang Y, Su X, Deng K, Yan G, Liang J. Intravenous injection of the oncolytic virus M1 awakens antitumor T cells and overcomes resistance to checkpoint blockade. Cell Death Dis. 2020 Dec 12;11(12):1062. doi: 10.1038/s41419-020-03285-0. PMID: 33311488; PMCID: PMC7733593.

[2] Cai J, Yan G. The Identification and Development of a Novel Oncolytic Virus: Alphavirus M1. Hum Gene Ther. 2021 Feb;32(3-4):138-149. doi: 10.1089/hum.2020.271. Epub 2021 Jan 22. PMID: 33261513.

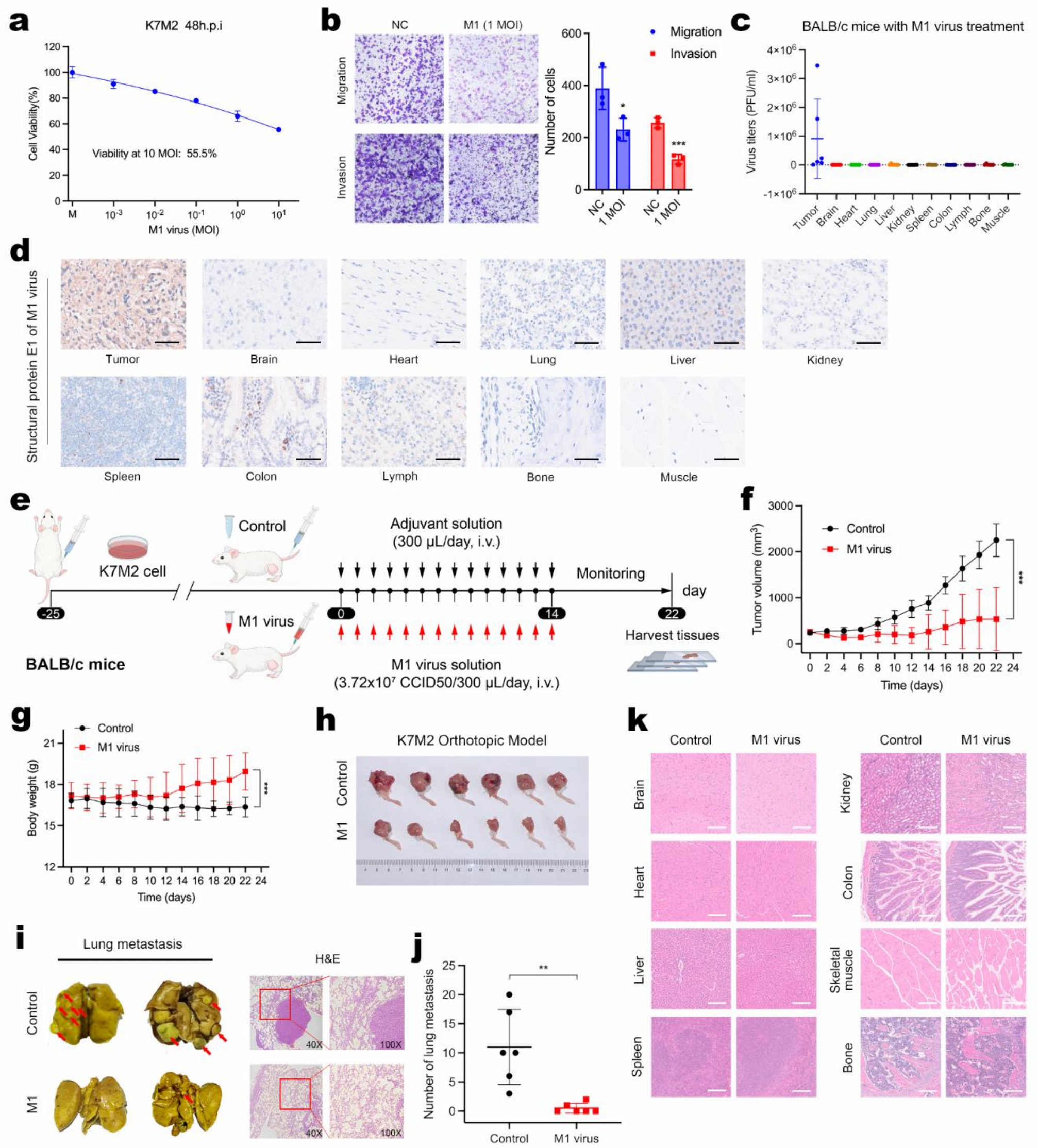

Extended Data Fig. 3 Antitumor activity and biodistribution of M1 virus in an immunocompetent murine osteosarcoma model

a, Viability of murine cell line K7M2 after infection with M1 virus at indicated MOIs for 48 hours. **b**,

Representative images of K7M2 cell migration and invasion assay after treatment with M1 virus (1 MOI) or NC (negative control). **c**, Viral titers (plaque-forming units, PFU) in indicated tissues harvested from BALB/c mice on day 4 post-administration of M1 virus. Data are presented as PFU per mL of tissue homogenate. n = 6. **d**, Representative immunohistochemistry (IHC) images showing the expression of the M1 virus E1 protein in various tissues from treated mice. Scale bar, 50 µm. **e**, Schematic illustration of the experimental timeline for the in vivo therapeutic study. K7M2 cells were implanted into the tibia of BALB/c mice to establish an orthotopic model, followed by intravenous administration of M1 virus or vehicle control. **f**,**g**, Tumor volume (**f**) and body weight (**g**) changes of tumor-bearing mice during the treatment period. Data are presented as mean ± SD (n=6 mice per group). **h**, Images of orthotopic tibial tumors from the control and M1 virus-treated groups at the experimental endpoint. **i**, Representative lung tissue images showing metastatic nodules (indicated by red arrows) (**i**, left). H&E images were taken at magnification of 40× and 100× (**i**, right). **j**, Quantification of lung metastatic foci from (**i**). n = 6. **k**, Representative H&E images revealed no tissue damage in the brain, heart, liver, spleen, kidneys, colon, skeletal muscle, or bone in M1 treated mice. These data are presented as the means ± SD and were analyzed by two-way ANOVA (**f, g**), and unpaired Student's t test (**b, j**). *, $P < 0.05$; **, $P < 0.01$; ***, $P < 0.001$.

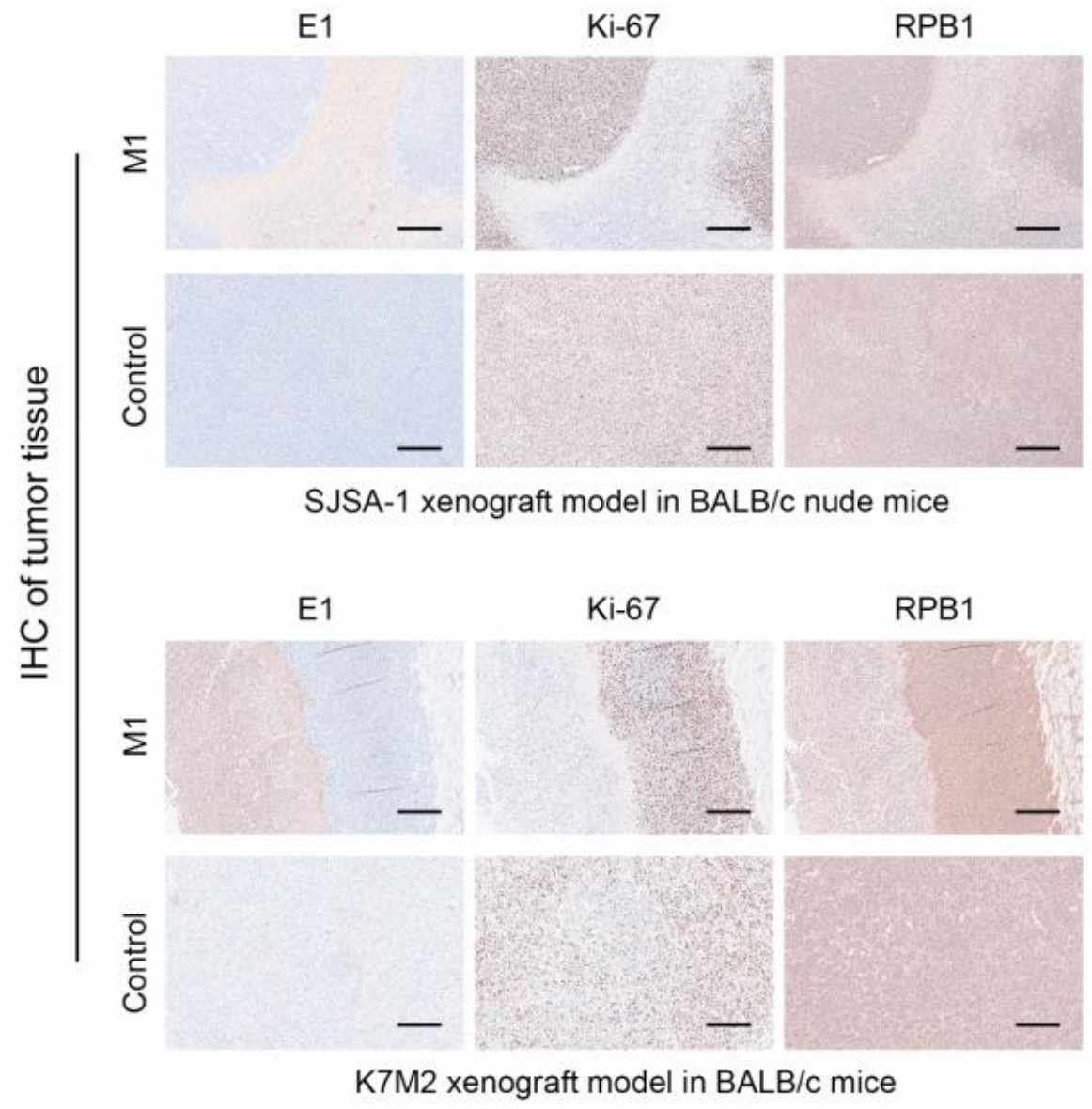

Extended Data Fig. 11 M1 virus downregulates RPB1 expression and suppresses proliferation in osteosarcoma xenografts, related to Fig. 5

Representative immunohistochemistry (IHC) images of tumor tissues from SJSA-1 orthotopic xenografts in BALB/c nude mice (**upper panel**) and K7M2 orthotopic xenografts in immunocompetent BALB/c mice (**lower panel**). Mice were treated with either control vehicle or M1 virus, and tumor tissues were harvested on day 4 post-administration. Sections were stained for the M1 viral protein E1 (infection marker), the proliferation marker Ki-67, and RPB1. Scale bar, 300 µm.

Additional comments:

• In Figure 1A, the replication kinetics of M1 are unclear. A virus growth curve across multiple MOIs is needed to demonstrate replication efficiency.

Thank you for your insightful comment regarding the replication kinetics of M1 in Figure 1A. In response, we have conducted a quantitative analysis based on GFP fluorescence intensity—which reflects viral replication—and have accordingly generated a viral growth curve. This new figure has now been included in the revised manuscript to clearly demonstrate the replication efficiency of M1 across multiple MOIs（Extended Data Fig. 1）.

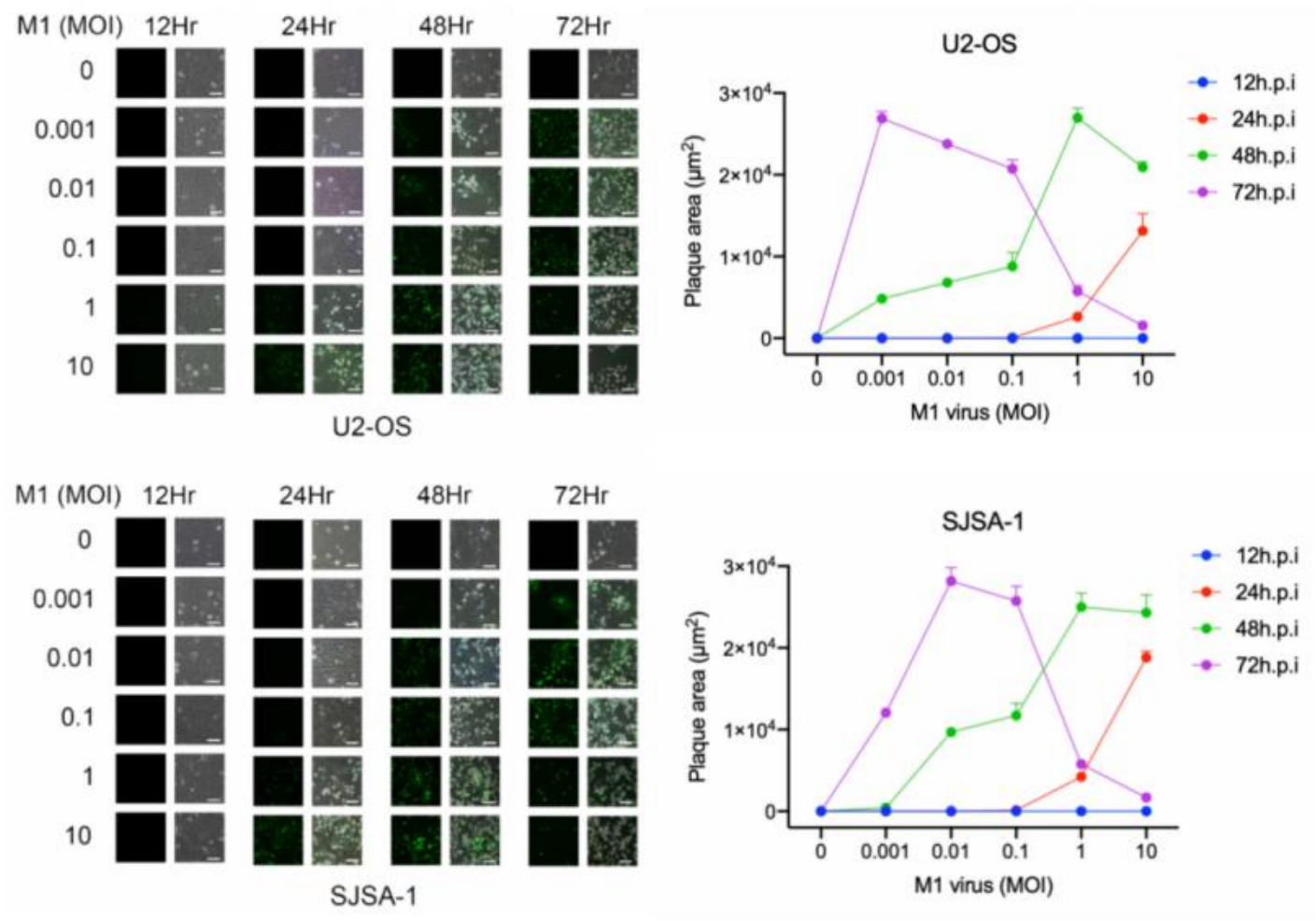

Extended Data Fig. 1| Viral Growth Quantification by Plaque Area, related to Fig. 1

• In Figure 1E, it is unclear why certain osteosarcoma lines are resistant. Is this due to impaired viral entry (e.g., lack of receptor), reduced replication, or failure to induce cytotoxicity despite replication? These questions could be addressed using M1-GFP tracking and viral titration over time. The mechanism of viral entry should also be discussed in the introduction, including any known receptors. How does infectivity compare between human and mouse cell lines? It is known that VSV, VV, and HSV exhibit reduced infectivity in murine models.

Thank you for your thoughtful comments regarding Figure 1E. To clarify the factors affecting the differential cytotoxicity of M1 virus across osteosarcoma cell lines, we performed M1-GFP tracking and viral titration to assess the infection efficiency in each cell line. The results indicate that M1 virus demonstrates favorable infection rates and viral titers in all tested osteosarcoma lines, including the murine osteosarcoma cell line K7M2. Furthermore, although variations in infection rates and viral replication were observed among these cell lines, these parameters did not strongly correlate with the anti-osteosarcoma efficacy of M1 virus (Extended Data Fig. 5a-c).

Additionally, as suggested, we have added a discussion of the reported viral entry receptors for M1 virus, namely LDLR and MXRA8, in the Introduction. We further examined the expression levels of these receptors in the osteosarcoma cell lines. Interestingly, while MXRA8 expression correlates with the observed oncolytic activity in osteosarcoma, the recently reported receptor LDLR—associated with GETV infection—shows an opposite trend in these cells. We speculate

this may be related to intrinsic characteristics of osteosarcoma as a tumor type. (Extended Data Fig. 5d)

Finally, we evaluated the infectivity and cytotoxic effect of M1 virus in the murine osteosarcoma cell line K7M2. The results show that K7M2 exhibits sensitivity to M1 virus comparable to that of human osteosarcoma cell lines(Extended Data Fig. 3a,b and Extended Data Fig. 5a). The oncolytic effect of M1 virus was also observed in immunocompetent mouse models (Extended Data Fig. 3e-k), and substantial tumor-selective enrichment of M1 virus was detected within tumor tissues in these models (Extended Data Fig. 3c-d). These findings suggest that M1 virus can efficiently infect both human and murine osteosarcoma cells, and that the observed intratumoral viral enrichment is attributable to the osteosarcoma tumor type rather than species differences between human and murine cells.

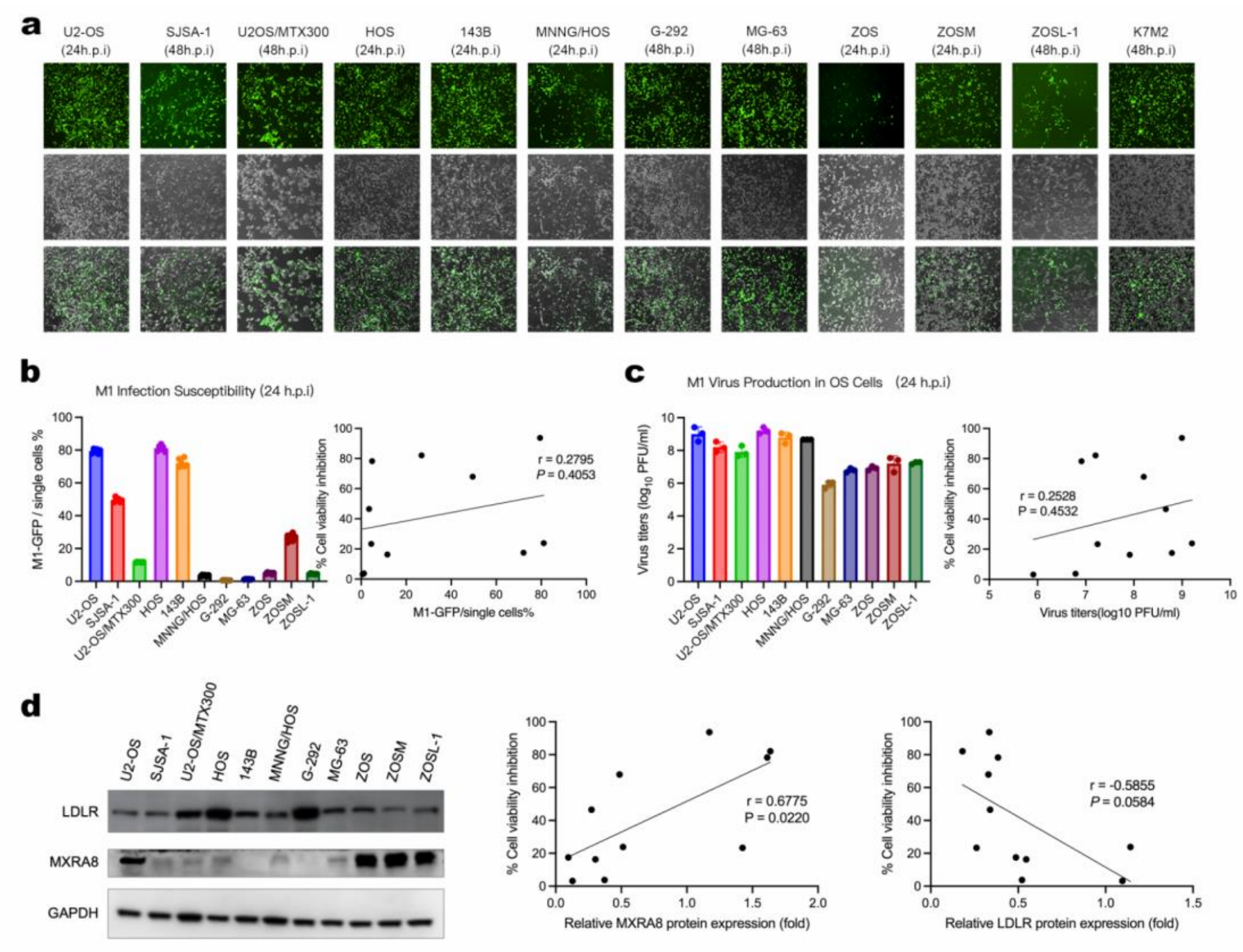

Extended Data Fig. 5：Characterization of M1 virus infection, replication, and receptor correlation in osteosarcoma cells, related to Fig. 3

a, Representative fluorescence images of 11 human osteosarcoma cell lines and the murine osteosarcoma cell line K7M2 infected with M1-GFP virus at the indicated time points (24 or 48 h.p.i). Scale bar, 100 μm. M1 virus demonstrated efficient infection and replication capabilities in all tested osteosarcoma cell lines. **b**, Left: Quantification of M1-GFP-positive cells by flow cytometry in 11 human osteosarcoma cell lines at 24 h.p.i. Right: Correlation analysis between M1-GFP positivity and M1 virus-induced cytotoxicity (cell viability at 24 h.p.i). No significant correlation was observed (r = 0.2795, P = 0.4053). **c**, Left: Viral titers in culture supernatants from 11 human osteosarcoma cell lines at 24 h.p.i, determined by TCID50 assay on BHK-21 cells and expressed as log_{10} PFU/mL. Right: Correlation analysis between viral titers and M1 virus-induced cytotoxicity. No significant correlation was detected (r = 0.2528, P = 0.4532). **d**, Left: Western blot analysis of previously reported alphavirus entry receptors (LDLR and MXRA8) in the indicated osteosarcoma cell lines. Middle: Correlation

analysis between MXRA8 expression levels and M1 virus-induced cytotoxicity, showing a significant positive correlation (r = 0.6775, P = 0.0220). Right: Correlation analysis between LDLR expression levels and M1 virus-induced cytotoxicity, showing no significant correlation (r = -0.5855, P = 0.0584). These data indicate that while M1 virus efficiently infects and replicates in osteosarcoma cells, neither infection rate nor viral production directly correlates with oncolytic efficacy, with only MXRA8 expression showing a positive correlation.

• In Figure 2I, immunohistochemistry (IHC) for viral proteins in tumor tissue should be included to confirm virus delivery. This would also serve as a positive control for IHC sensitivity.

Thank you for your valuable suggestion. In response to your comment regarding immunohistochemistry (IHC) for viral proteins in tumor tissue, we performed IHC staining using an antibody against the M1 viral E1 protein across multiple mouse tissues. The results demonstrated significantly stronger E1 staining intensity within tumor tissues compared to normal tissues, thereby confirming successful intratumoral virus delivery.(Extended Data Fig. 2b)

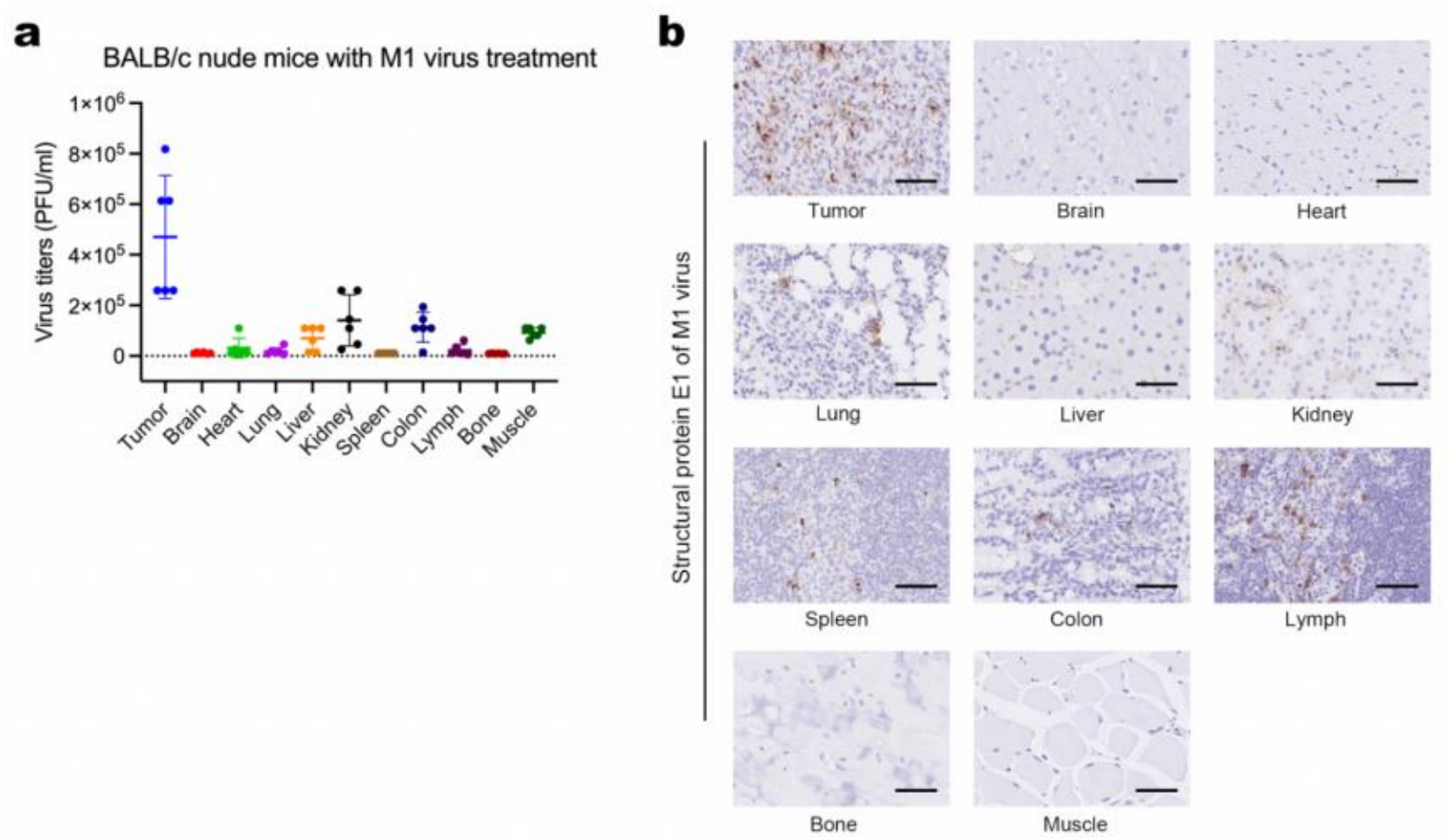

Extended Data Fig. 2 Distribution of M1 virus in mouse tissues, related to Fig. 2
a, Viral titers (plaque-forming units, PFU) in indicated tissues harvested from mice following systemic administration of M1 virus. Data are presented as PFU per mL of tissue homogenate. **b**, Representative immunohistochemistry (IHC) images showing the expression of the M1 virus structural protein E1 in corresponding tissue sections. Scale bar, 50 µm. These complementary assays were performed to accurately assess the biodistribution of M1 virus *in vivo*.

• The xenograft model used in Figure 2 is not clearly described. More information should be provided in the main text or methods.

Thank you for your valuable feedback regarding the description of the xenograft model used in Figure 2. We appreciate your comment and have revised the manuscript accordingly to provide a clearer description in the Methods section (Page 12, line 531th).

• The use of iRFP for in vivo imaging is suboptimal due to its lower sensitivity compared to luciferase. For biodistribution, viral titration remains the gold standard, although qPCR is an acceptable complementary method.

Thank you for your valuable suggestion. In response, we have now supplemented our data with viral titer measurements in tissue specimens from the mouse experiments (Extended Data Fig. 2a), along with immunohistochemistry (IHC) analysis for viral protein detection (Extended Data Fig. 2b). These additional results further enrich the understanding of M1 virus distribution in mice.

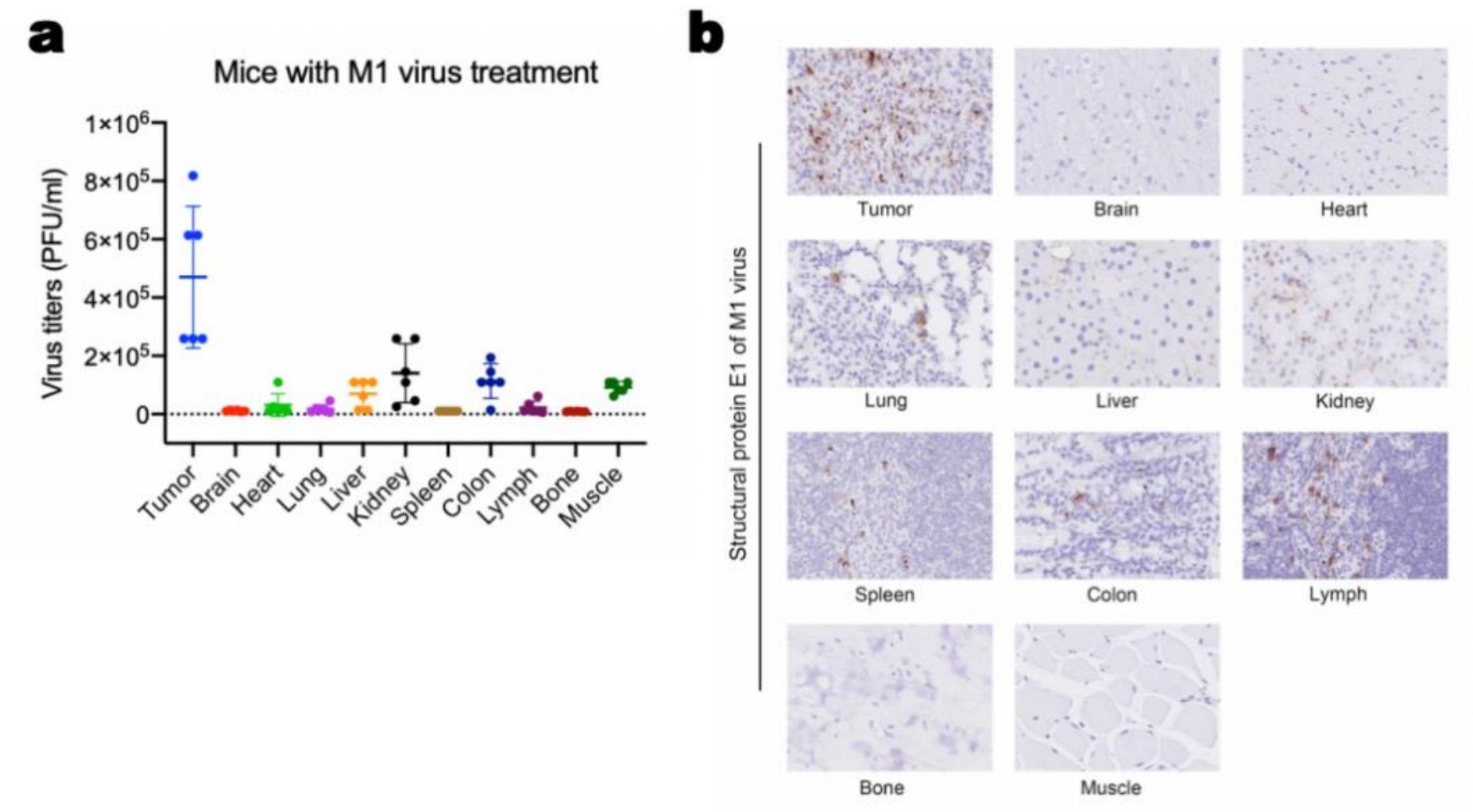

Extended Data Fig. 2 Distribution of M1 virus in mouse tissues, related to Fig. 2
a, Viral titers (plaque-forming units, PFU) in indicated tissues harvested from mice following systemic administration of M1 virus. Data are presented as PFU per mL of tissue homogenate. **b**, Representative immunohistochemistry (IHC) images showing the expression of the M1 virus structural protein E1 in corresponding tissue sections. Scale bar, 50 µm. These complementary assays were performed to accurately assess the biodistribution of M1 virus *in vivo*.

• Details of virus production and titration methods are missing from the Materials and Methods and should be completed.

In response to your valuable suggestion, we have supplemented the "Materials and Methods" section with a detailed description of the virus production and titration protocols (Page 11, line 518th).

• The virus used in this study should be sequenced, and the full genome sequence should be included in the supplementary materials.

As suggested, we have provided the complete genome sequence of the M1 virus as Table S2 in the revised version of the manuscript(Page 11, line 518th).

Revised Response to Reviewers

**Manuscript #**:	NCOMMS-25-43655A
**Title**:	Alphavirus M1 disrupts super-enhancer-driven oncogenic transcription via non-structural protein NSP2 in osteosarcoma

Responds to Reviewer #1:

Dear reviewer:

We sincerely thank you for the thoughtful and constructive assessment. We appreciate the reviewer's recognition that the revised manuscript presents an interesting and well-developed mechanistic story and that the additional experiments strengthened the mechanistic aspects of the work. We have carefully revised the manuscript to address the remaining concerns, particularly by moderating the in vivo and translational claims and by clarifying the scope and limitations of the present study.

Comment 1: In vivo antitumor efficacy remains modest and model limited

Reviewer comment: The core efficacy data still come from a single human osteosarcoma xenograft model in nude mice and a single murine osteosarcoma model in immunocompetent mice, both treated with very intensive daily intravenous dosing over 14 days. Tumor growth is delayed during treatment, but tumors regrow promptly after cessation in the xenograft setting, and the overall magnitude of benefit does not justify the repeated description of "remarkable" or "potent" in vivo efficacy.

Response:

We agree with the reviewer that the *in vivo* efficacy should not be overstated. In response, we have systematically removed or softened terms such as "remarkable," "potent," and similar efficacy-oriented wording across the manuscript. We now describe the *in vivo* findings more cautiously as tumor-suppressive activity or supportive preclinical observations in the tested models. We have also explicitly acknowledged in the Discussion that the *in vivo* evaluation was limited to one human xenograft model and one syngeneic murine model, which does not fully establish robustness or generalizability across osteosarcoma contexts.

These revisions were made in the Abstract (Page 1, line 19th, 26th), Introduction (Page 2-3, line 69th, 73th, 90th), Results (Page 3,6, line 108-109th, 120-122th, 126-127th, 252th), and Discussion (Page 6-8, line 271th, 286-290th, 344-357th).

Comment 2: Dosing regimen and translational relevance are not resolved

Reviewer comment: The daily dosing regimen remains difficult to reconcile with strong translational claims, particularly since alternative regimens and intratumoral viral kinetics were not assessed.

Response:

We appreciate this important point and agree that the prior framing of translational relevance was too strong relative to the current data. In the revised manuscript, we now explicitly state that the in vivo activity was observed under an intensive daily intravenous dosing schedule and that we did not systematically compare alternative regimens or define intratumoral viral kinetics under different schedules. We therefore no longer present the current study as establishing a clinically feasible or optimized therapeutic strategy. Instead, we now frame the work as providing a mechanistic foundation and supportive preclinical evidence for future studies aimed at dose/schedule optimization.

These clarifications have been added to the Discussion (Page 8, 344-357th), and efficacy/translational wording has been moderated throughout the manuscript.

Comment 3: Scope of the in vivo mechanism and immune contribution

Reviewer comment: Although the additional IHC data support the mechanism in vivo, the study still only limitedly explores how this mechanism interfaces with antitumor immunity in osteosarcoma.

Response:

We agree that the contribution of antiviral and antitumor immunity has not been dissected in depth in the current study. Our primary goal here was to define the tumor-intrinsic molecular mechanism by which M1/NSP2 targets RPB1 and suppresses super-enhancer-associated oncogenic transcription. To better reflect this scope, we have now explicitly stated in the Discussion that, although the syngeneic model provides initial support for activity in an immunocompetent setting, the contribution of antiviral and antitumor immunity remains to be clarified in future studies.

This point is now incorporated into the newly expanded limitations paragraph in the Discussion (Page 8, 344-357th).

Comment 4: Translational claims appear overstated relative to the data

Reviewer comment: The manuscript repeatedly uses strong language about “remarkable anti-tumor efficacy,” “potent *in vivo* and in vitro anti-osteosarcoma effect,” “favorable safety,” and direct clinical translatability, which are not fully supported by the current evidence.

Response:

We fully agree and thank the reviewer for this clear guidance. In response, we have carefully revised the manuscript to remove or soften these expressions throughout. Examples of changes include:

- removal of wording such as “potent” and “remarkable” when describing *in vivo* efficacy;
- revision of safety-related language from generalized claims of “favorable safety” to more data-bounded descriptions such as no overt toxicity or no overt histopathological abnormalities in the examined organs under the tested conditions;

- replacement of direct or strong translational claims with more cautious wording indicating supportive preclinical evidence, candidate biomarker, and further preclinical evaluation/optimization;
- addition of a limitations paragraph clarifying that the current study should be interpreted primarily as mechanistic, with supportive *in vivo* observations, rather than as establishing an optimized therapeutic strategy.

We believe these revisions have substantially improved the balance and accuracy of the manuscript.

Responds to Reviewer #3:

Comment:

I don't have any further comments. The authors have addressed my concerns well.

Response:

We sincerely thank Reviewer #3 for the positive evaluation of our revised manuscript and for recognizing that the previous concerns were addressed satisfactorily. We appreciate the reviewer's time and consideration.
